# A digital imagery-competing task intervention for stopping intrusive memories in trauma-exposed health-care staff during the COVID-19 pandemic in the UK: a Bayesian adaptive randomised clinical trial

**DOI:** 10.1016/S2215-0366(25)00397-9

**Published:** 2026-03

**Authors:** Amy C Beckenstrom, Michael B Bonsall, Alfred Markham, Owen Slade, Varsha Ramineni, Lalitha Iyadurai, Zunaid Islam, Julie Highfield, Thomas Jaki, Guy M Goodwin, Rebecca Dias, Rebecca Daniels, Asad Malik, Charlotte Summers, Jonathan Kingslake, Emily A Holmes

**Affiliations:** a P1vital Products, Wallingford, UK; b Mathematical Ecology Research Group, Department of Biology, University of Oxford, Oxford, UK; c St Peter's College, University of Oxford, Oxford, UK; d Intensive Care Society, London, UK; e University of Regensburg, Regensburg, Bavaria, Germany; f MRC Biostatistics Unit, University of Cambridge, Cambridge, UK; g Department of Psychiatry, University of Oxford, Oxford, UK; h Victor Phillip Dahdaleh Heart & Lung Research Institute, University of Cambridge, Cambridge, UK; i Department of Women's and Children's Health, Uppsala University, Uppsala, Uppsala County, Sweden; j School of Psychology, Faculty of Environmental and Life Sciences, University of Southampton, Southampton, UK

## Abstract

**Background:**

Psychological trauma, such as witnessing an untimely or gruesome death, commonly provokes intrusive memories that might persist for days to years with adverse effects on individual mental and physical health and functioning. Despite the global prevalence of trauma, scalable evidence-based interventions are absent. Reducing the impact of intrusive memories is crucial for people frequently exposed to trauma, such as health-care workers. This study aimed to determine whether a brief digital imagery-competing task intervention (ICTI) reduced intrusive memory frequency after 4 weeks. Harms were also assessed.

**Methods:**

The GAINS-02 decentralised digital, parallel-group Bayesian adaptive randomised controlled trial tested a brief ICTI against an active control and treatment as usual to determine the effect on reducing intrusive memory frequency. Health-care workers in facilities that admitted patients with COVID-19 during the pandemic, who had experienced one or more traumatic events and reported at least three intrusive memories in the week before screening were randomised 2:2:1 (ICTI to active control to treatment as usual) via block randomisation (web-based). ICTI and active control participants were masked to treatment allocation, and both had one guided session then optional self-use. ICTI involved image-based memory retrieval then Tetris computer gameplay with mental rotation. The active control involved a music-listening task. Study statisticians were masked to ICTI and active control group. The primary outcome was the number of intrusive memories in week 4 (controlling for baseline), which was evaluated on an intention-to-treat basis. Treatment effects for the intervention group versus the comparator groups were assessed using Bayes regression analyses. Harms were assessed through adverse event reporting and interim analyses on primary outcome. People with lived experience were involved from study conception and throughout the research and writing process. The trial was pre-registered at clinicaltrials.gov (NCT05616676) and is completed.

**Findings:**

Between Dec 8, 2022, and Sept 15, 2023, 176 participants were screened and 99 included (ICTI n=40, active control n=39, treatment as usual n=20) with mean age 41·2 years (SD 10·2; range 21–62). Of these 99 participants, 85 (86%) self-identified as women and 89 (90%) as White. Bayesian analyses gave robust evidence that ICTI reduced intrusive memories at week 4: ICTI participants reported fewer intrusive memories (median 0·5 [IQR 0·0–5·0]) compared with the active control (active control 5·0 [3·0–11·5]; Bayes factor [BF]_active control>ICTI vs active control=ICTI_ 114·1; β_active control>ICTI_ 1·29 [95% CrI 0·64–2·00]) and treatment as usual (median 5·0 [IQR 2·5–8·0]; BF_treatment as usual>ICTI vs treatment as usual=ICTI_=15·8; β_treatment as usual>ICTI_ 1·21 [95% CrI 0·49–1·98]) groups. No harms were detected for ICTI relative to the active control and treatment as usual. The most reported adverse event (n=7) was COVID-19. Two adverse events involved burden of diary recording. Serious adverse events were hospitalisations unrelated to study procedures (n=6).

**Interpretation:**

This study shows that ICTI reduces intrusive memory frequency and post-traumatic stress disorder symptoms among health-care workers exposed to trauma. As a brief, scalable digital intervention, ICTI shows promise for mitigating consequences of trauma on mental health, an important and unmet need for health-care personnel and systems worldwide.

**Funding:**

Wellcome Trust, Swedish Research Council, UK Medical Research Council, and National Institute for Health and Care Research.

## Introduction

Intrusive memories of traumatic events (eg, severe injury, death, dying, and sexual assault) are the hallmark symptom of post-traumatic stress disorder (PTSD) and acute stress disorder. Intrusive memories are involuntary and recurrent recollections that appear suddenly in the mind's eye, typically as visual images of a trauma. Intrusive memories are commonly referred to as flashbacks and influence other PTSD symptoms (eg, persistent avoidance, negative alterations in cognition or mood, and hyperarousal). A study with over 17 000 trauma-exposed adults in the USA found 13% had PTSD,^1^ and 95% of those with PTSD had intrusive memories of trauma. For a PTSD diagnosis, the sensitivity of these memories was 95·14% and specificity was 51·91%, with many individuals experiencing subclinical levels of intrusive memories after trauma exposure. Regardless of diagnostic status, experiencing intrusive memories after trauma can be distressing and debilitating. For example, health-care workers exposed to work-related trauma during the COVID-19 pandemic described vivid and unwanted intrusive memories such as “the eyes of a young patient dying while resuscitation fails”.^2^ Although trauma-focused psychological treatments are effective for PTSD,^3^ they are resource intensive, typically requiring 8–12 sessions with specialist therapists,^4^ which limits their scalability and accessibility, and they are not recommended for people facing ongoing trauma. Intrusive memories offer a focal treatment target for post-traumatic stress.^5^ Network models of PTSD show that intrusive memories are centrally connected to other PTSD symptoms,^6^ suggesting solely targeting intrusions should affect post-traumatic stress more broadly.

**Research in context**

**Evidence before this study**

Health-care systems worldwide face increasing strain from untreated trauma among health-care professionals. Intrusive memories of traumatic events, commonly called flashbacks, are the core symptom of post-traumatic stress disorder (PTSD)—distressing visual images that intrude involuntarily and repeatedly, and reflect re-experiencing of the trauma. If intrusive memories are reduced, there should be a beneficial effect on other PTSD symptoms. First-line PTSD treatments recommended by UK National Institute for Health and Care Excellence guidelines (trauma-focused cognitive behavioural therapy and eye-movement desensitisation and reprocessing) are effective, but they are resource-intensive (requiring multiple therapist-led sessions), often difficult to access due to availability, and not always well tolerated because they require the individual to talk about their trauma. A 2021 Cochrane review found digital PTSD treatments moderately better than the waitlist control but not superior to active comparators. Treatments are lacking for those with ongoing trauma exposure or those who prefer not to verbalise trauma.

We developed a neuroscience-informed, imagery-competing task intervention (ICTI) to reduce the frequency of intrusive memories. In an ICTI session, the individual briefly retrieves a visual intrusive memory followed by a visuospatial task (here, Tetris gameplay with mental rotation) to disrupt memory restorage and reduce intrusiveness. ICTI requires one guided session, no detailed trauma disclosure, and allows self-referral via presence of a single symptom (intrusive memory).

A search of PubMed, Web of Science, and APA PsycNet from database inception to April 8, 2025, using the search terms (“traum*” OR “PTSD”) AND (“intrusive memor*” OR “involuntary memor*” OR “intrusive imag*”) AND (“intervention” OR “treatment” OR “therapy”), identified two hospital-based trials delivering face-to-face ICTI on the day of trauma, which reduced intrusive memories at one week. ICTI delivered at longer post-trauma intervals (weeks to years) was reported in single-case and single-group face-to-face studies; one trial showed intrusive memory reduction at four weeks, and one cross-over trial had null results. One study with PTSD patients was independent from our research group. The first digital ICTI integrating within a secure platform both memory reminder and visuospatial task (Tetris gameplay with mental rotation) was evaluated in the optimisation randomised controlled trial (RCT) GAINS-01 with trauma-exposed health-care staff, finding intrusive memories and PTSD symptoms reduced at 4 weeks, with high acceptability and no intervention-related adverse events.

**Added value of this study**

The GAINS-02 RCT evaluates a brief digital ICTI for trauma-exposed individuals, which is doubly controlled (active control and treatment as usual) and delivered via a fully integrated web platform from the day of trauma to months later, with one initial navigator-guided session. ICTI is tested over a 6-month post-trauma window under conditions of ongoing trauma. Results show that intrusive memories are reduced at 4 weeks, benefits are sustained over 6 months, and most participants achieved symptom elimination. No harms were detected for individuals receiving ICTI relative to individuals in the control group. Advantages for scalability include brief human-assisted contact time (1 h) using trained digital-navigators without mental health qualifications, and remote intervention delivery on widely available devices via a secure web platform. Thus, GAINS-02 provides the first evidence that a single guided-session digital intervention can sustainably reduce intrusive memories and other PTSD symptoms, even for individuals with ongoing trauma exposure.

**Implications of all the available evidence**

ICTI addresses various barriers by being brief, flexible, and repeatable after one guided session, and available on-demand through a secure digital platform. Compared with current psychological therapies, digital ICTI is less time consuming, simpler to complete and low distress, includes a game associated with low stigma, does not require a mental health professional, and is precise by focusing on a single symptom. ICTI requires neither clinician assessment nor a PTSD diagnosis, enables self-referral, and does not require detailed trauma discussion. ICTI represents a promising, scalable digital intervention or adjunctive therapy in trauma care, which could expand access, reduce barriers, and help contribute towards trauma treatment globally.

Delivering health care, although rich in social meaning and satisfaction, can involve frequent exposure to death, disease, and distress. Frontline health-care workers are repeatedly exposed to psychological traumas, such as gruesome or unexpected patient deaths, and they report high rates of intrusive memories.^7^ For example, PTSD prevalence among National Health Service (NHS) staff in the UK increased from 13% before the COVID-19 pandemic^8^ to 25% during the pandemic,^9^ compared with a global lifetime prevalence of 4% in the general population before the pandemic.^10^ For health-care workers, shift-based work and strained health-care systems restrict access to conventional therapies. Scalable, flexibly accessible interventions after work-related trauma are needed to support health-care staff wellbeing and retention, and to sustain global health-care infrastructures. Preserving health-care staff wellbeing is a major international public concern. Effective and scalable interventions after trauma have been absent,^11^ and solutions that fit into working lives are required. Innovation is needed to improve the wellbeing of health-care workers.

We developed the imagery competing task intervention (ICTI), a novel neuroscience-informed approach including Tetris gameplay with mental rotation grounded in two decades of experimental research.^12^, ^13^ Delivering ICTI within hours of real-world trauma showed promise when delivered in-person by specialist psychologists.^14^, ^15^ In the GAINS-01 trial,^2^, ^16^ we remotely delivered a digital version of ICTI to intensive care unit (ICU) health-care workers who had experienced work-related trauma during the COVID-19 pandemic using the i-spero digital platform, and at longer time intervals post-trauma. After an initial guided session, ICTI was self-administered as required. Compared with a delayed-access control group (with usual care), ICTI substantially reduced intrusive memories from a baseline median of 14 per week to one per week at 4 weeks,^2^ with additional clinical and functional benefits.^16^ When the delayed control group later received ICTI, reductions were replicated.

The GAINS-01 trial^2^, ^16^ lacked an active control group,^17^ raising the possibility that improvements were due simply to researcher contact. The GAINS-02 trial addresses this limitation by comparing ICTI with an active control, matched for delivery via i-spero. Both interventions involve one researcher-guided session and, thereafter, self-guided repetition and symptom monitoring. The key difference is that Tetris is a visuospatial puzzle task, whereas the music-listening active control (Mozart) is auditory and was selected due to perceived cognitive benefits and therapeutic effects on stress.^18^

Another limitation of GAINS-01^2^, ^16^ was limited follow-up, leaving some uncertainty as to whether effects were durable. GAINS-02 addresses this limitation by adding evaluations at weeks 12 and 24, and by expanding recruitment to all NHS health-care staff (rather than ICU staff only).^19^ GAINS-02 uses the full 20-item version (rather than 4-item version) of the PTSD Checklist (PCL-5),^20^ allowing determination of whether reducing intrusive memories influenced broader PTSD symptoms.

We conducted a decentralised Bayesian adaptive randomised controlled trial (RCT) with ICTI, active control, and treatment as usual groups. The primary objective was to determine whether a brief digital ICTI reduced intrusive memory frequency at week 4, controlling for the number of intrusive memories in the baseline week. Benefits were assessed in sequential Bayesian interim analyses with pre-specified stopping rules for week 4 intrusive memories reduction. Harms were assessed by adverse event reporting throughout study duration, and sequential Bayesian interim analyses for week 4 intrusive memory increases.

## Methods

### Study design and participants

We conducted a three-group, parallel-group, decentralised digital Bayesian adaptive RCT to evaluate a brief, remotely delivered, digital ICTI in the UK. The trial was approved by the Wales Research Ethics Committee 4 (22/WA/0277), including one substantial amendment (adding feedback interviews at weeks 12–24) and seven non-substantial amendments. People with lived experience of work-related trauma were involved throughout study conception, research, and writing process. Lived experience within the core research team was fundamental to the study design, including the adaptive approach and choice of endpoints. A collaborative partnership with the Intensive Care Society included refining study materials and advice on recruitment strategies. Qualitative interviews with participants from the GAINS-01 trial^19^ led to modifications such as intervention instructions and how to schedule usage. Overall, continual lived experience involvement helped the study remained relevant, feasible, and grounded in the everyday working context for health-care staff as the COVID-19 pandemic progressed.

Participants were recruited through the Intensive Care Society membership networks, targeted social media advertisements, seminars, and posters at NHS sites. Recruitment materials provided a link to the study website, a participant information sheet, and a video explanation of intrusive memories. Interested individuals completed an online pre-screening eligibility questionnaire and scheduled a meeting with a researcher, during which eligibility criteria were verified and written informed consent was obtained.

Inclusion criteria were being age 18 years or older; being fluent in English; having worked in an NHS facility admitting patients with COVID-19 during the pandemic; having experienced one or more traumatic event related to this work, meeting DSM-5 PTSD Criterion A (eg, exposure to actual or threatened death); having experienced intrusive memories of the traumatic events; having experienced three or more intrusive memories in the week before screening; having internet access; and being willing to provide consent and able to complete study procedures and be contacted by the study team. Exclusion criteria were having fewer than three intrusive memories during the baseline week and participation in previous studies of this intervention.

Before being randomly assigned, participants completed a 7-day online diary to confirm having three or more intrusive memories in the baseline week.

### Randomisation and masking

Participants were randomly assigned (2:2:1) to ICTI, the active control, or treatment as usual via a remote, secure web-based system (P1vital ePRO) with block randomisation (random block sizes of multiples of 5, 85% allocation). Randomisation followed baseline questionnaires (appendix p 104).

Participants were masked to allocation to ICTI or active control group. The consent sheet described both as brief online interventions that include a 25 min cognitive task, and both were delivered via i-spero. The treatment as usual group was not masked. Participants entered outcome measures into the web-based system without the aid of a researcher and scoring was automated. Statisticians were masked to active control and ICTI treatment allocation but could potentially infer treatment as usual assignment due to the unequal allocation ratio. Researchers supporting guided sessions and those interacting with participants were not masked and did not contribute to analyses.

### Procedures

Participants in the ICTI and active control groups had an initial 1 h researcher-guided navigation session via Microsoft Teams to mitigate barriers such as low digital literacy and technical or practical issues, and to facilitate optimal use of the interventions and intrusive memory diary (appendix p 96).

In the ICTI group, the initial session included instructions, explanatory videos, and multiple-choice questions. Participants listed their intrusive memories, selected one, and briefly brought it to mind. After learning about mental rotation, participants played Tetris® (embedded in i-spero) for 20 min using mental rotation. Participants were instructed to monitor intrusive memories using the optional daily record in i-spero. Participants had access to ICTI throughout the 24 weeks, with optional researcher support and SMS, email, and phone reminders during the first 4 weeks. At each use, participants briefly recalled one intrusive memory's image, rated its vividness on a 1–5 scale (appendix p 101), and played Tetris for 20 min using mental rotation. Vividness, game-score, and play duration were recorded.

In the active control group, the initial session matched ICTI in duration and structure. The session included instructions, a British Broadcasting Company informational podcast about Mozart, multiple-choice questions, and listening to Mozart's String Duo No.1 K.423 for 20 min. Participants had 24 weeks of access, with optional support and reminders. At each use, participants received instructions to avoid distractions before listening for 20 min, with listening duration recorded.

Participants in the treatment as usual group were informed they could continue any treatment for their intrusive memories and would receive intervention access after the trial. Similarly, active control and ICTI participants were able to take part in any other treatment as usual and were not required to discontinue existing mental health treatments.

The researcher-guided session for the intervention group was delivered by a digital navigator^21^ team of graduate-level research assistants (AMar and ZI) and a clinical psychologist (LI). Researchers received training and competency assessments, supported by ongoing expert supervision (led by EAH; appendix p 96).

### Outcomes

The primary outcome was a comparison of the total number of intrusive memories of traumatic events recorded in week 4, controlling for the number at baseline. All participants completed a 7-day online diary during week 4 (days 22–28) to record intrusive memory counts.^2^, ^16^

Before randomisation, participants completed a baseline 7-day online diary recording numbers of intrusive memories of work-related trauma, prompted daily by email or SMS.^2^, ^16^ Instructions included an intrusive memory definition (“mental images from a traumatic event that pop suddenly into your mind when you don’t want them to”) and the question: “have you had any intrusive memories on [date]?” (yes or no). If yes, participants entered the number of intrusive memories. Those with three or more intrusive memories during the baseline week were eligible to complete baseline questionnaires, including trauma type and demographics (eg, self-reported gender and ethnicity). Expectancy of treatment effect was collected using the Credibility and Expectancy Questionnaire (CEQ)^22^ immediately before the researcher guided session for ICTI and active control participants, and the day after randomisation for treatment as usual participants (appendix p 105).

Secondary outcomes included intrusive memory counts during week 12 (days 78–84) and week 24 (days 162–168). Other secondary outcomes assessed at baseline, week 4, week 12, and week 24 included PTSD symptoms (PCL-5 20-item),^20^ insomnia (Sleep Condition Indicator 2-item), depression (Patient Health Questionnaire-2), anxiety (Generalised Anxiety Disorder-2), general functioning and quality of life (12-item WHO Disability Assessment Schedule [WHODAS] and EuroQol [EQ-5D-5L]), occupational measures (Scale of Work Engagement and Burnout [SWEBO]), sick days (in the previous 4 weeks), intention to leave job, and bespoke intrusive memory questionnaire (appendix p 103).

Exploratory outcome assessments included intervention usage and within-session ICTI ratings, collected in i-spero throughout study. Changes to health and work were assessed at week 4, week 12, and week 24 (including new traumatic events), and an acceptability feedback questionnaire was administered at week 4. The post-hoc analysis of a clinically meaningful difference used ≥5 points on the PCL-5, based on Ehlers and colleagues.^23^

The safety population included all consented participants*.* Participants reported adverse events, new treatments, and medical issues through open-ended questions at week 4, week 12, and week 24, or directly to researchers throughout the trial. Sequential Bayesian interim analyses tested for harmful effects (ie, an increase in intrusive memories at 4 weeks) with pre-specified stopping criteria.

All outcome assessments were completed remotely and self-reported using ePRO and i-spero with automated scoring. Due to login issues, primary outcome data for one treatment as usual participant were received via SMS, transcribed onto source documentation, reviewed by study monitors, and included in analyses.

### Statistical analysis

The trial was prospectively registered with clinicaltrials.gov (NCT05616676). Pre-specified analyses are described in the study statistical analysis plan, available on the Open Science Framework. The analysis population for primary and secondary analyses was intention-to-treat, including all randomly assigned participants. Analyses were completed in R (version 4.1.2) using the brms package.^24^

Target enrolment was 150 participants (ICTI [n=60], active control [n=60], and treatment as usual [n=30]) with pre-specified Bayesian interim analyses. The Data Monitoring Committee (DMC) could recommend stopping early should sufficient positive or negative evidence emerge (pre-defined Bayes Factor [BF]>20). For evidence of positive effect, we tested for fewer week 4 intrusive memories in ICTI compared with the active control. For negative effects, we tested for fewer week 4 intrusive memories in treatment as usual compared with ICTI. Simulations (90% power; 0·05 significance) based on previous studies estimated that the 150-participant sample size would be reasonable to detect appropriate effect sizes (ICTI *vs* waitlist control of *d*=0·85,^2^ and ICTI *vs* active controls of *d*=0·57^25^, ^26^ [philosophy podcast] and *d*=0·71^13^ [Tetris only, without memory reminder]). This sample size was then assumed sufficient to detect ICTI versus treatment as usual differences (with effect size *d*=0·75), and ICTI versus active control differences (with effect size *d*=0·6).

Sequential interim analyses using Bayesian regression assessed between-group difference in week 4 intrusive memories to determine if stopping criteria were met. The first analysis occurred after 20–25 participants completed primary outcome, with subsequent analyses every 4–10 additional participants. Evidence of the benefits of ICTI (fewer intrusive memories at week 4) compared with active control (BF>20) was reached after the eleventh interim analysis (BF=27·62; appendix p 5). On DMC recommendation, recruitment continued until this criterion was exceeded in two subsequent analyses. The fourteenth and final interim analysis occurred after 99 participants were randomly assigned (40 for ICTI, 39 for active control, and 20 for treatment as usual).

At no point were the stopping criteria met due to evidence for harm (more week 4 intrusive memories in ICTI *vs* treatment as usual; all BF<20; appendix p 5).

For the primary outcome analysis, Bayesian regression models were fitted in which the primary outcome (intrusive memory count over multiple timepoints) was modelled using a Poisson linear mixed model (observation level random effects Poisson). Baseline intrusive memory count and treatment assignment were fitted as fixed effects with a random intercept effect for participants. Bayesian hypothesis testing was performed using BFs with the main hypothesis that ICTI would yield fewer intrusive memories at week 4 than the active control and treatment as usual. Week 4 intrusive memory totals were modelled following the methods shown in the appendix (p 97). Participants with no week 4 intrusive memory data (all diary days missing) were defined as not assessable for the primary analysis. Missing values were imputed for participants with partial diary completion (at least one diary day completed; appendix p 98). Partly completed cases were excluded in sensitivity analyses.

Potential outliers were identified through residual and Cook's distance versus leverage plots from a fitted frequentist model (one treatment as usual datapoint was identified as a potential outlier). Outliers were included in the primary analysis and removed in sensitivity analyses.

Secondary outcomes were analysed using Bayesian regression with baseline scores, treatment, week, and the treatment-by-week interaction as fixed effects and random intercepts for participants. Details of the methods used for the secondary outcomes and other analyses (not pre-specified) are shown in the appendix (pp 99–100).

Guidelines for the interpretation of BFs in terms of strength of evidence are in the appendix (p 12). For example, a BF >100 is termed extreme evidence towards the alternative hypothesis (ie, in favour of ICTI); by comparison a BF in the range 1–3 is classified as anecdotal evidence.

### Role of the funding source

The funders of the study had no role in study design, data collection, data analysis, data interpretation, or writing of the report.

## Results

**Figure 1:**
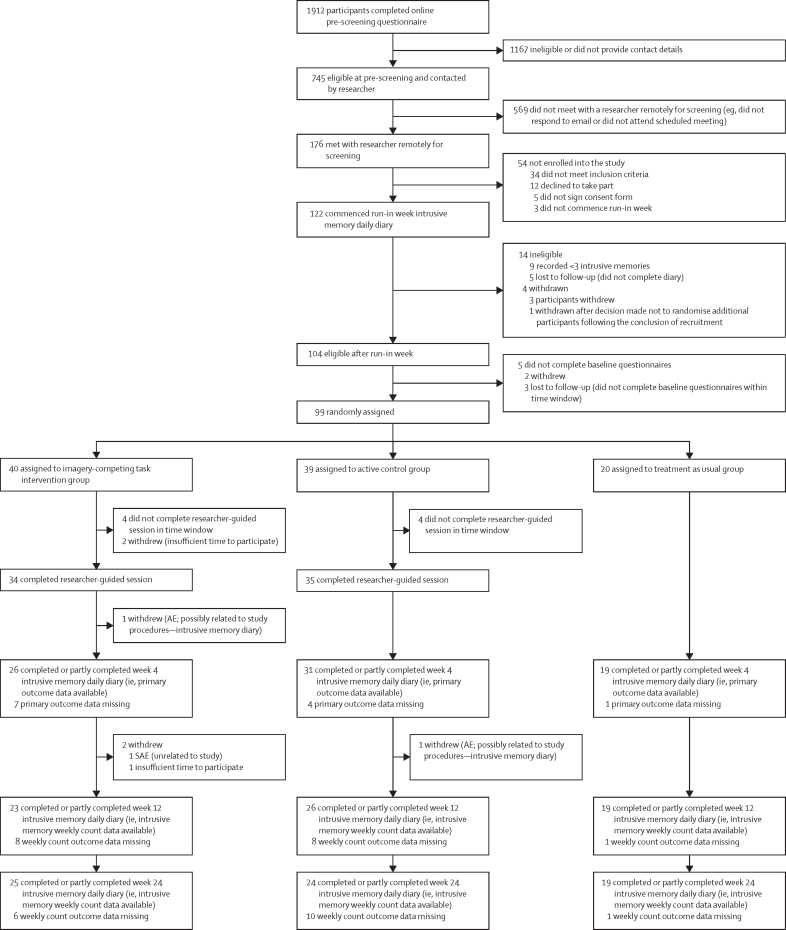
CONSORT diagram AE=adverse event. SAE=serious adverse event.

Between Dec 8, 2022, and Sept 15, 2023, 176 participants were screened; 122 were eligible and began the baseline intrusive memory diary (figure 1). After excluding nine participants for insufficient intrusive memories, eight lost to follow-up, and six withdrawals, 99 participants were randomised in a 2:2:1 ratio to ICTI (n=40), active control (n=39), and treatment as usual (n=20). At week 4, 76 participants provided complete or partial primary outcome data (ICTI=26, active control=31, treatment as usual=19; figure 1). Study completion was on March 19, 2024.

**Figure 2:**
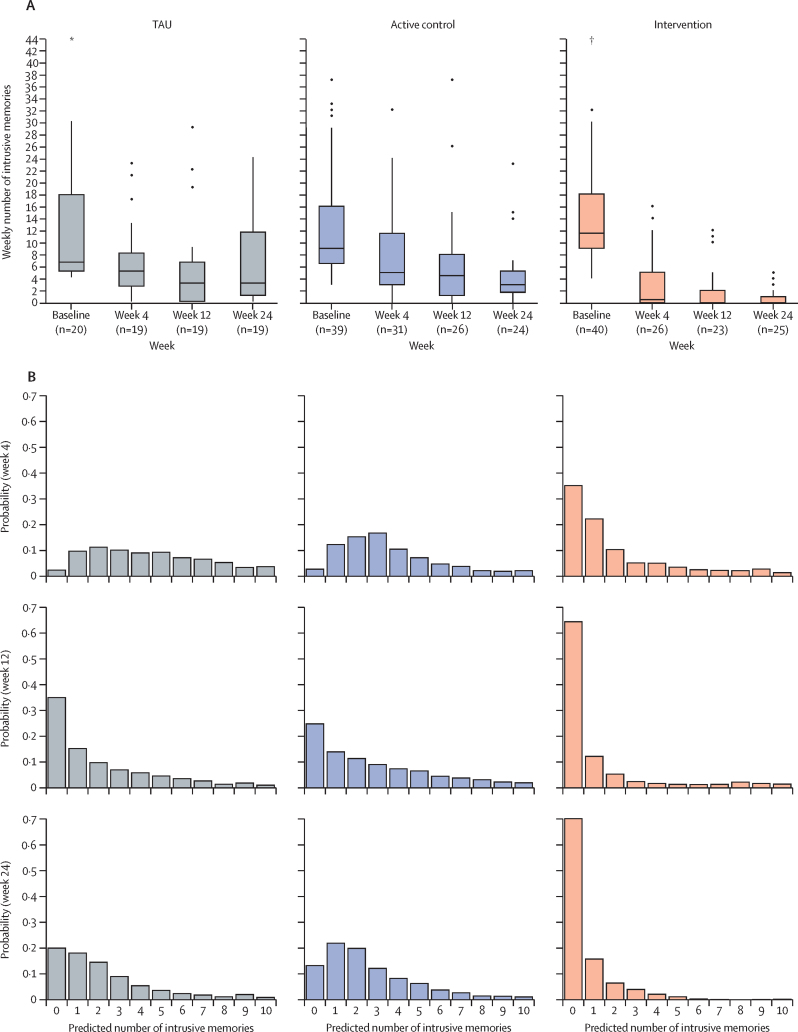
Number of intrusive memories over time per group (A) and their probability distribution (B) Boxplots for number of intrusive memories at baseline, week 4, week 12, and week 24 for each group, showing the median (midline), the third and first quartile (upper and lower limits of box), and whiskers (1·5 × IQR). Outliers are represented by dots (>1·5 times the IQR above third quartile or below first quartile). All outliers are included in these boxplots. Probability distributions of number of intrusive memories at week 4 (row 1), week 12 (row 2), and week 24 (row 3) per group (exploratory analysis). TAU=treatment as usual. *Outlier at 75. †Outlier at 84.

Participants mean age was 41·2 (SD 10·2) years, 85 (86%) self-identified as women, 12 (12%) self-identified as men, 89 (90%) self-identified as White, and 74 (75%) worked full-time (table). Roles included nursing (47 [47%]), allied health professionals (seven [7%]), and doctors (six [6%]). Baseline demographics, health characteristics, and previous trauma were similar across arms (table). Most participants (66 [67%]) had trauma more than 3 months before baseline, and 50 (51%) reported ongoing work-related trauma exposure. Post-hoc one-way ANOVA showed no significant between-groups baseline difference in number of work-related traumatic events during the COVID-19 pandemic (F[2,96]=2·16, p=0·12). For those with available data (n=68), 51% worked in intensive care. The baseline week median intrusive memory count was 10·0 (IQR 6·5–17·5), which was similar across groups (figure 2; appendix pp 6–8, 105).

**Table:** Summary table of baseline characteristics and experiences of ongoing trauma during the study for ITT population (n=99)

			**Overall (n=99)**	Active control*(n=39)	**ICTI (n=40)**	**TAU (n=20)**
Age, years			41·2 (10·2; 21–62)	43·3 (10·9; 21–61)	40·2 (10·4; 25–62)	39·1 (8·0; 24–60)
Gender						
	Woman		85 (85·9%)	33 (84·6%)	34 (85·0%)	18 (90·0%)
	Man		12 (12·1%)	6 (15·4%)	4 (10·0%)	2 (10·0%)
	Gender-variant or non-binary		1 (1·0%)	0	1 (2·5%)	0
	Prefer not to answer		1 (1·0%)	0	1 (2·5%)	0
Highest level of education						
	Secondary school (to age 16 years)		1 (1·0%)	1 (2·6%)	0	0
	Sixth form or equivalent (to age 18 years)		5 (5·1%)	3 (7·7%)	2 (5·0%)	0
	Bachelor's degree or equivalent		72 (72·7%)	31 (79·5%)	28 (70·0%)	13 (65·0%)
	Master's degree		21 (21·2%)	4 (10·3%)	10 (25·0%)	7 (35·0%)
Ethnicity						
	Asian		3 (3·0%)	2 (5·1%)	0	1 (5·0%)
	Chinese		1 (1·0%)	0	1 (2·5%)	0
	Mixed		3 (3·0%)	1 (2·6%)	2 (5·0%)	0
	White		89 (89·9%)	34 (87·2%)	36 (90·0%)	19 (95·0%)
	Other		3 (3·0%)	2 (5·1%)	1 (2·5%)	0
Marital status						
	Single		20 (20·2%)	7 (17·9%)	9 (22·5%)	4 (20·0%)
	Living apart from partner		7 (7·1%)	4 (10·3%)	3 (7·5%)	0
	Married or cohabiting		60 (60·6%)	21 (53·8%)	26 (65·0%)	13 (65·0%)
	Divorced or separated		11 (11·1%)	6 (15·4%)	2 (5·0%)	3 (15·0%)
	Widowed		1 (1·0%)	1 (2·6%)	0	0
	Other		0	0	0	0
Hours working per week			34·1 (13·0)	31·7 (14·6)	33·8 (13·5)	39·4 (6·2)
Time as health-care professional (years)			16·4 (10·7)	17·7 (12·4)	15·6 (9·4)	15·6 (10·0)
Employment status						
	Working full time		74 (74·7%)	27 (69·2%)	30 (75·0%)	17 (85·0%)
	Working part time		14 (14·1%)	6 (15·4%)	5 (12·5%)	3 (15·0%)
	Sick leave		3 (3·0%)	2 (5·1%)	1 (2·5%)	0
	Student		3 (3·0%)	3 (7·7%)	0	0
	Retired		1 (1·0%)	1 (2·6%)	0	0
	Other		4 (4·0%)	0	4 (10·0%)	0
NHS job role						
	Allied health professional		7 (7·1%)	3 (7·7%)	4 (10·0%)	0
	Doctor		6 (6·1%)	1 (2·6%)	2 (5·0%)	3 (15·0%)
	Health-care support worker		7 (7·1%)	4 (10·3%)	3 (7·5%)	0
	Nurse		47 (47·5%)	16 (41·0%)	18 (45·0%)	13 (65·0%)
	Other or unknown		1 (1·0%)	1 (2·6%)	0	0
	Missing		31 (31·3%)	14 (35·9%)	13 (32·5%)	4 (20·0%)
Previous health and trauma (yes or no, n=yes)						
	Treated for or diagnosed with mental health problems		70 (70·7%)	26 (66·7%)	28 (70·0%)	16 (80·0%)
	Current disorder?		43 (43·4%)	16 (41·0%)	18 (45·0%)	9 (45·0%)
	Past disorder?		41 (41·4%)	11 (28·2%)	19 (47·5%)	11 (55·0%)
	Are you receiving any current treatments or medications for these (eg, psychological treatment, counselling, medication, alternative therapies)		40 (40·4%)	15 (38·5%)	15 (37·5%)	10 (50·0%)
		Psychotherapies or counselling	23 (23·2%)	8 (20·5%)	9 (22·5%)	6 (30·0%)
		Psychotropic medication	27 (27·3%)	12 (30·8%)	8 (20·0%)	7 (35·0%)
Timeframe of work-related traumatic events experienced at baseline						
	Within the past 24 h		6 (6·1%)	1 (2·6%)	4 (10·0%)	1 (5·0%)
	Within the past month		2 (2·0%)	0	1 (2·5%)	1 (5·0%)
	Between 1 and 3 months ago		2 (2·0%)	0	1 (2·5%)	1 (5·0%)
	More than 3 months ago		66 (66·7%)	24 (61·5%)	26 (65·0%)	16 (80·0%)
	Ongoing exposure to traumatic events is part of my job during the COVID-19 pandemic		50 (50·5%)	25 (64·1%)	17 (42·5%)	8 (40·0%)
	Number of work-related traumatic events experienced/witnessed during the COVID-19 pandemic		29·9 (54·7)	19·9 (35·0)	29·3 (40·8)	50·8 (94·2)
	Number of non-work-related traumatic events experienced/witnessed during the COVID-19 pandemic		3·3 (5·7)	4·1 (7·0)	3·1 (4·6)	2·2 (4·8)
Which of the following categories best fit the work-related traumatic events that you have experienced or witnessed during COVID-19, of which you have intrusive memories? (yes or no, n=yes)						
	A traumatic or tragic death of a patient		87 (87·9%)	35 (89·7%)	34 (85·0%)	18 (90·0%)
	A severe or unsuccessful resuscitation		70 (70·7%)	25 (64·1%)	30 (75·0%)	15 (75·0%)
	Witnessing events surrounding colleague who has fallen ill or died of COVID-19		55 (55·6%)	23 (59·0%)	23 (57·5%)	9 (45·0%)
	Situation where the care of a patient failed or did not go as planned		72 (72·7%)	29 (74·4%)	29 (72·5%)	14 (70·0%)
	Threats or violence against health-care professionals		52 (52·5%)	19 (48·7%)	21 (52·5%)	12 (60·0%)
	Event involving sudden increased risk of COVID-19 infection		71 (71·7%)	26 (66·7%)	34 (85·0%)	11 (55·0%)
	A traumatic or tragic event where a patient reminded you of yourself, a family member or friend		64 (64·6%)	27 (69·2%)	26 (65·0%)	11 (55·0%)
	Event involving extremely distressed or grieving relatives of patients		72 (72·7%)	29 (74·4%)	28 (70·0%)	15 (75·0%)
	Being faced with suicide or suicide attempt		36 (36·4%)	15 (38·5%)	14 (35·0%)	7 (35·0%)
	Other		12 (12·1%)	7 (17·9%)	4 (10·0%)	1 (5·0%)
Experiences of ongoing trauma						
	Have you experienced or witnessed any new work-related traumatic events (yes or no, n=yes)					
		Week 4	21 (31·3%)	6 (23·1%)	6 (25·0%)	9 (52·9%)
		Week 12	19 (29·7%)	3 (12·5%)	8 (38·1%)	8 (42·1%)
		Week 24	17 (26·2%)	8 (33·3%)	5 (20·0%)	4 (25·0%)
	How many new work-related traumatic events have you experienced or witnessed					
		Week 4	0·9 (1·8)	0·6 (1·2)	0·8 (1·4)	1·5 (2·7)
		Week 12	1·0 (2·0)	0·5 (1·1)	1·5 (2·6)	1·1 (1·9)
		Week 24	0·9 (2·2)	1·0 (2·0)	1·0 (2·8)	0·8 (1·4)
	How many new traumatic events that were not work-related have you experienced or witnessed					
		Week 4	0·1 (0·4)	0·2 (0·5)	0·1 (0·3)	0·1 (0·2)
		Week 12	0·3 (0·7)	0·2 (0·5)	0·5 (0·9)	0·4 (0·8)
		Week 24	0·2 (0·5)	0·3 (0·8)	0·2 (0·4)	0·1 (0·3)
Weekly number of intrusive memories at baseline			10·00 (6·50–17·50)	9·00 (6·50–16·00)	11·50 (9·00–18·00)	6·50 (5·00–17·75)
Expectancy of intervention effect (CEQ)						
	At this point, how logical does the intervention offered to you seem?		6·0 (2·1)	6·8 (1·3)	5·9 (1·9)	4·4 (2·5)
	At this point, how successful do you think this intervention will be in reducing your intrusive memories?		5·0 (2·0)	5·8 (1·5)	5·4 (1·9)	2·9 (1·7)
	How confident would you be in recommending this intervention to a friend who experiences similar problems?		5·1 (2·5)	6·2 (2·0)	5·4 (2·2)	2·8 (2·3)
	By the end of the intervention period (4 weeks), how much improvement in your intrusive memories do you think will occur?		4·7 (2·2)	5·6 (1·6)	5·0 (2·0)	2·6 (2·1)
	At this point, how much do you really feel that the intervention will help you to reduce your intrusive memories?		4·9 (2·4)	5·9 (1·8)	5·4 (2·4)	2·3 (1·7)
	By the end of the intervention period (4 weeks), how much improvement in your intrusive memories do you really feel will occur?		4·7 (2·4)	5·7 (1·8)	5·0 (2·4)	2·2 (1·7)
	CEQ total score		30·4 (12·2)	35·9 (8·2)	32·1 (11·4)	17·2 (10·3)

Data shown are mean (SD), mean (SD; range), n (%), or median (IQR). Counts are presented with percentages calculated according to the number of participants (n) with available data in the ITT population. Expectancy of intervention effect was assessed using the CEQ, which was administered not at baseline but after randomisation. CEQ=Credibility and Expectancy Questionnaire. ICTI=imagery-competing task intervention. ITT=intention to treat. TAU=treatment as usual.

* Data on age were missing for one participant in active comparator.

For the primary outcome, the Bayesian regression analysis compared the number of intrusive memories in week 4 between groups, controlling for baseline on an intention-to-treat basis (n=76). The ICTI group showed strong evidence of reduced intrusive memories, with a median of 0·5 (IQR 0·0–5·0) compared with the active control (5·0 [3·0–11·5]) and treatment as usual groups (5·0 [2·5–8·0]; figure 2; appendix pp 6–8).

The ICTI group experienced a beneficial treatment effect compared with the active control (BF=114·1) and treatment as usual (BF=15·8) groups. ICTI was 114·1 times more likely to reduce intrusive memories than active control and 15·8 times more likely than treatment as usual (appendix pp 9–12). From Bayesian regression model predictions, there were 72% fewer intrusive memories in ICTI than the active control group and 70% fewer in ICTI than the treatment as usual group. The posterior means for differences between groups were 1·29 (95% credible interval [CrI] 0·64–2·00) for the active control group and 1·21 (0·49–1·98) for the treatment as usual group. The estimated posterior probabilities of fewer intrusive memories in ICTI than active control and treatment as usual groups at week 4 were almost 1.

Re-running regression analyses using different non-informative priors showed no considerable variation in posterior distributions of model parameters (appendix p 13). All models were checked for convergence and goodness of fit (appendix pp 9–12, 14–16). Sensitivity analyses showed consistent results when excluding imputed data, excluding outliers (appendix pp 17–19), excluding one active control participant with imputed intrusive memories, and imputing missing primary outcome data using grouped mean imputation (BF=733·89 *vs* active control; BF=37·30 *vs* treatment as usual). Frequentist sensitivity analysis showed significantly fewer intrusive memories at week 4 in ICTI compared with both other groups (appendix pp 20–21).

Secondary outcome analyses showed that the ICTI group were mostly free of intrusive memories at week 12 (median=0·0 [IQR 0·0–2·0]; n=23) and week 24 (0·0 [0·0–1·0]; n=25). At week 12, medians were 4·5 (IQR 1·25–8·0; n=26) for the active control group and 3·0 (0·0–6·5; n=19) for the treatment as usual group; at week 24, medians were 3·0 (1·75–5·25; n=24) for the active control group and 3·0 (1·0–11·5; n=19) for the treatment as usual group (appendix pp 6–8). See the appendix for more detail on all secondary outcomes (p 103).

Bayesian negative binomial mixed models supported a strongly beneficial treatment effect for ICTI compared with the active control group (week 12: BF=23 199; week 24: BF=infinity) and treatment as usual group (week 12: BF=541; week 24: BF=infinity; figure 2; appendix pp 22–25). The estimated posterior probabilities of fewer intrusive memories in ICTI than the active control and treatment as usual groups at weeks 12 and 24 were almost 1. All models were checked for convergence, goodness of fit, and insensitivity to varying priors (appendix pp 26–31).

**Figure 3:**
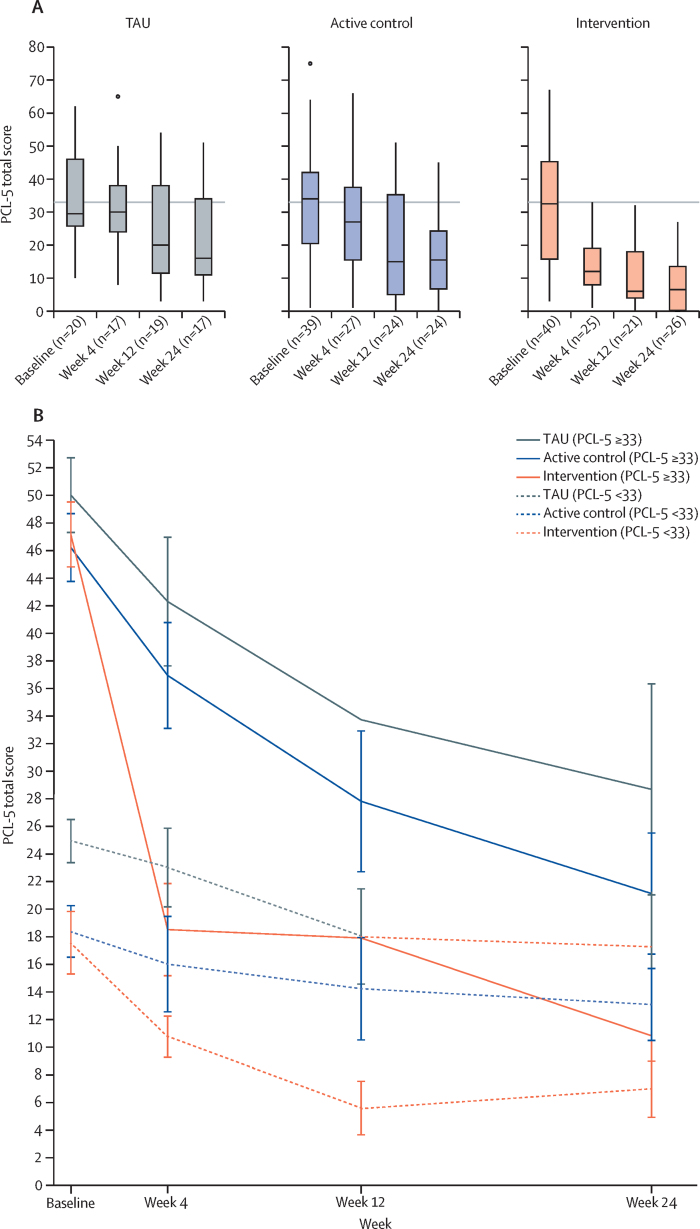
PTSD symptom severity (PCL-5 total) across timepoints per group (A) Boxplots showing PTSD symptom severity (PCL-5 total) at baseline, week 4, week 12, and week 24, by treatment group. The median is the midline, the box shows the third and first quartile, and whiskers extend 1·5 times the IQR. Outliers are dots (>1·5 times the IQR above the third quartile and below the first quartile). The grey horizontal line shows the cutoff point for probable PTSD. All outliers are shown. (B) Line graph of PCL-5 total scores for subgroups with and without probable PTSD at baseline. Solid lines represent participants with probable PTSD, and dashed lines represent those without. Mean (SE) scores are shown at each timepoint. PCL-5=PTSD Checklist for DSM-5 (20-item version). PTSD=post-traumatic stress disorder. TAU=treatment as usual.

PTSD symptoms (PCL-5) decreased more after ICTI than both the active control group (week 4: BF=1546; week 12: BF=117; week 24: BF=119) and treatment as usual group (week 4: BF=7732; week 12: BF=304; week 24: BF=176), indicating extreme evidence of a positive treatment effect (figure 3; appendix pp 12, 32–42). The estimated posterior probabilities of a clinically meaningful difference (≥5 points on the PCL-5) were 0·95 (week 4), 0·80 (week 12), and 0·79 (week 24) for ICTI versus the active control group, and 0·99 (week 4), 0·91 (week 12), and 0·87 (week 24) for ICTI versus the treatment as usual group.

Post-hoc analyses for participants with probable PTSD at baseline (on PCL-5, n=48 [49%]) showed reduced PTSD symptoms in ICTI compared with both comparator groups at all post-baseline timepoints (very strong-to-extreme evidence, BF>36·9), and for those with subthreshold PTSD, results indicated reduced PTSD symptoms with at least strong evidence at all post-baseline follow-ups (all BF>11; figure 3; appendix pp 32–40, 43–51).

ICTI reduced insomnia compared with the treatment as usual group (BF=23·4) and the active control group (BF=4·3) at week 4. This difference was maintained at week 12 and week 24 (appendix pp 32–40, 52). For anxiety and depression, ICTI reduced scores at week 4 compared with the active control group (anxiety: BF=13·4; depression: BF=80·1) and the treatment as usual group (anxiety: BF=53·4; depression: BF=77·6), but results were variable at week 12 and week 24 (appendix pp 32–40, 53–54).

WHODAS scores showed extreme evidence of a beneficial effect of ICTI, with BFs of 508 (week 4), 402 (week 12), and 2230 (week 24) versus the treatment as usual group. Compared with the active control group, BFs favouring ICTI were 138·1 (week 4), 2·8 (week 12), and 21·0 (week 24; appendix pp 32–40, 55). EQ-5D-5L scores showed benefits of ICTI versus both comparators at week 4 for anxiety/depression, but not other items. The effects were null or mixed at follow-ups (appendix pp 32–40, 56–61).

There was lower burnout (active control: BF=125; treatment as usual: BF=1364) and higher work engagement (SWEBO; active control: BF=28·4; treatment as usual: BF=143) at week 4 after ICTI. At weeks 12 and 24, BFs were mostly near 1, and 95% CrIs for group differences were narrow and centred close to zero, suggesting any remaining differences were probably small (appendix pp 62–63). There was strong evidence for lower intention to leave job after ICTI compared with the active control group (BF=15·1) and the treatment as usual group (BF=10·5) at week 4, but anecdotal evidence (BF<3) at weeks 12 and 24 (appendix p 64). Impact on sick day number was inconsistent (appendix pp 32–40, 65). Relative to both comparators, ICTI participants rated less distress, interference, and functional impact from intrusive memories, especially at week 24 (appendix pp 66–72).

Some of the estimated parameters for secondary outcomes showed moderate sensitivity to the choice of priors (eg, appendix pp 42, 45–76). However, in all such cases, estimates derived using less restrictive priors (ie, normal distributions with mean=0 and SD=10 or 20) remained consistent.

ICTI's acceptability was higher (mean 7·92 [SD 2·33]) than that of the active control group (6·50 [2·79]; (0=“not at all”, 10=“very”; appendix pp 73–74). Compared with active control participants, ICTI participants recorded greater willingness to use their intervention in the future, recommend it, and endorse NHS use (appendix pp 75–76). Guided-session adherence was 97% in ICTI (33 of 34) and 97% in the active control group (34 of 35), and subsequent self-use frequency was comparable (ICTI mean 6·91 [SD 7·40]; active control 5·57 [9·55]; appendix p 77).

New work-related traumas were reported by 21 (31%) participants at week 4, 19 (30%) at week 12, and 17 (26%) at week 24; further details can be found in the appendix (pp 78–81).

For intervention credibility, CEQ mean total scores were 32·14 (SD 11·42) for ICTI and 35·90 (8·23) for the active control group (table), with greater credibility in active control than ICTI (BF 20·1; appendix pp 82–84).

At baseline, ten (50%) participants were receiving mental health treatments in the treatment as usual group, 15 (38%) in the ICTI group, and 15 (38%) in the active control group (table). Of these participants, six (treatment as usual), eight (active control), and nine (ICTI) received psychotherapy, and seven (treatment as usual), 12 (active control), and eight (ICTI) received psychotropic medication. For treatments started during the study, four (treatment as usual), one (active control), and none (ICTI) received psychotherapy, and three (treatment as usual), none (active control), and one (ICTI) received psychotropic medication (appendix pp 85–89).

To explore the likelihood of eliminating intrusive memories, post-hoc Bayesian mixed model regressions and BF analysis showed the probability of a participant achieving zero intrusive memories when using ICTI increased over time (week 4: 0·35; week 12: 0·65; week 24: 0·70; figure 2). ICTI participants had a 70% chance of zero intrusive memories by week 24, compared with 13% for active control participants and 20% for treatment as usual participants.

**Figure 4:**
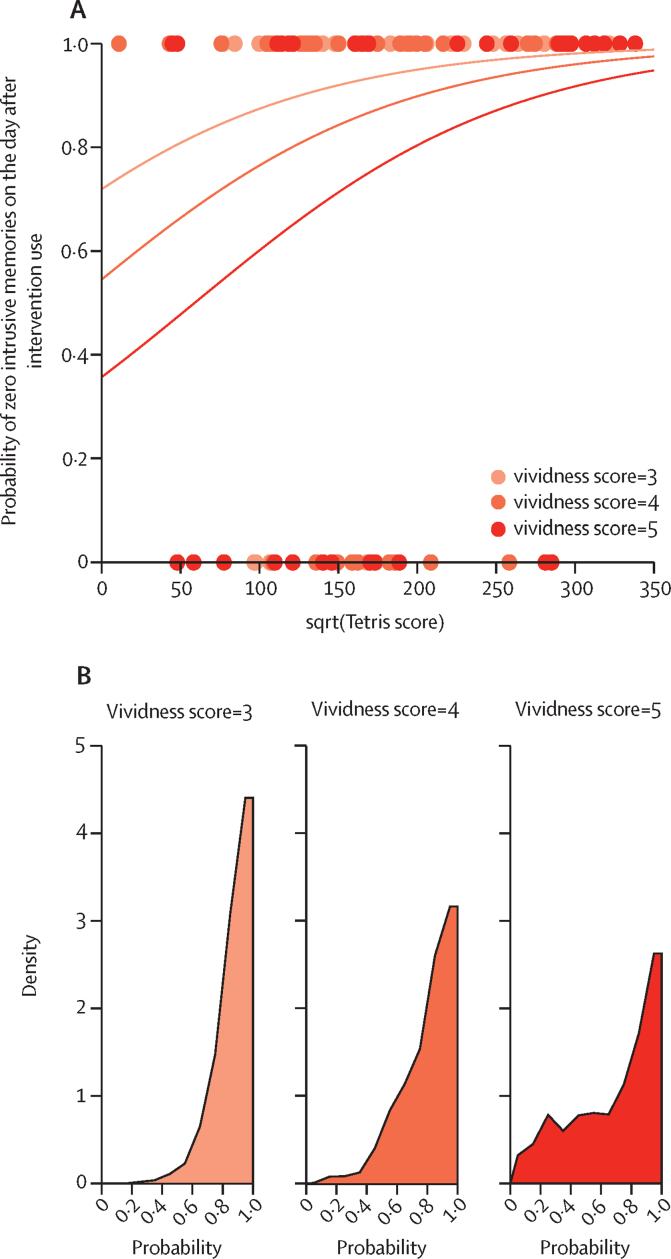
Mechanistic predictions of effects of memory recall and competing task on ICTI success using Bayesian mixed model regression analysis (A) Probability of zero intrusive memories (reported in daily intrusive memory record in i-spero the day after an ICTI usage) as a function of vividness ratings (memory recall intensity) and Tetris scores (competing task) during that ICTI session. Higher vividness ratings (darker orange is higher) decrease the probability of zero intrusive memories as Tetris scores increase. Dots show individual datapoints; lines show fits from logistic regression analysis. (B) Distribution of expected outcomes from posterior predictive distributions for vividness levels. Lower vividness (pale orange) results in more successful outcomes than higher vividness levels (dark orange), suggesting that weaker reactivated memories lead to a greater chance of intrusive memory elimination. ICTI=imagery-competing task intervention.

To explore mechanisms explaining intrusive memory elimination, the probability of a targeted intrusive memory reducing to zero the day after using ICTI was analysed in a post-hoc Bayesian mixed model regression, incorporating within-session vividness ratings and Tetris gameplay scores. Successful elimination of intrusive memories (zero) was associated with lower memory recall vividness and higher Tetris gameplay scores (figure 4). BF analysis confirmed that lower vividness scores were stronger predictors of zero intrusive memories than higher vividness scores. Sampling expected values from the posterior predictive distributions highlighted the variance in intrusive memory reduction between vividness categories (figure 4). Successful reduction in intrusive memories was expected to be high for all vividness categories. However, the variance was lower for memory recalls associated with lower vividness levels than those memories recalled with higher vividness levels.

51 adverse events occurred in 37 (37%) participants, with 23 adverse events recorded in 15 ICTI participants, 18 adverse events in 13 active control participants, and ten adverse events in nine treatment as usual participants. The most common adverse event (n=7) was SARS-CoV-2 infection. Two adverse events involved burden of using the daily diary to record intrusive memories (in the active control group, it was related to study procedures; in the ICTI group, it was related to study procedures, potentially the intervention, or both). Both these participants withdrew. Six serious adverse events (SAEs; three hospitalisations in the ICTI group and three hospitalisations in the active control group) were unrelated to study procedures. No participants reported multiple SAEs (appendix pp 85–89).

At no point during the 14 interim Bayesian analyses were stopping criteria met due to harm (appendix p 5). Final analyses showed clear evidence of no harm with BFs <0·0001 (ICTI *vs* active control) and 0·0006 (ICTI *vs* treatment as usual), indicating safety.

To explore participant retention, post-hoc Bayesian contingency analyses showed no evidence for difference in retention between ICTI and comparator groups, with anecdotal evidence favouring no difference between ICTI and active control (BF=0·67) or active control and treatment as usual (BF=0·73). There was moderate evidence for poorer retention in ICTI compared with treatment as usual (BF=8·12). Bayesian logistic regression analysis showed baseline variables did not predict outcome data missingness (BF→0; appendix pp 90–93).

## Discussion

Our study showed that a brief, remotely delivered digital ICTI effectively reduced intrusive memories in health-care workers exposed to work-related trauma, outperforming both the active control and treatment as usual. The ICTI group had 72% fewer intrusive memories than the active control group, and 70% fewer than the treatment as usual group. Benefits on the primary outcome were sustained across 24 weeks, with posterior probabilities of fewer intrusive memories in ICTI compared with the comparator groups >99%. Most ICTI participants reported zero intrusive memories at weeks 12 and 24, with 28 (70%) of participants symptom-free by week 24. ICTI effects occurred after a single guided session without requirement for a qualified trauma therapist.

The ICTI was effective in improving the full range of PTSD symptoms, with improvements sustained over 24 weeks. Targeting a single symptom (intrusive memories) appeared to have a pluralistic effect across other symptoms. At week 4, ICTI was associated with at least a 95% posterior probability of clinically greater reductions in PTSD symptoms compared with control groups, sustained at ≥79% at week 24. Reductions in PTSD symptoms occurred regardless of baseline PTSD status, with no ICTI participants meeting criteria for probable PTSD by week 12 or week 24. Anxiety, depression, and general functioning improved at week 4. Results highlight the potential of digital ICTI to provide sustained intrusive memory relief and PTSD symptom reduction.

The lower efficiency of using an active control task (listening to music) compared with the ICTI (visuospatial task) suggests that visual imagery disruption is a key mechanism driving effects. Since the active control was well matched to ICTI regarding delivery platform, researcher contact, and ongoing access to i-spero, and was perceived as more credible than ICTI, placebo effects are unlikely to explain the results.

Both active groups involved one guided session led by a digital navigator,^21^ followed by self-guided use. Navigators received training and supervision but were not required to have a mental health qualification. Participants could self-refer, and adherence and acceptability were high. These features support the scalability of ICTI.

The persistence of intrusive, overly strong maladaptive memories underlies PTSD. Based on memory-related neuroscience, we developed a retrieval-dependent behavioural ICTI (Tetris gameplay with mental rotation)^27^ to reduce intrusiveness by competing with mental imagery^29^ during memory updating.^13^ Theory predicts that if a given intrusive memory is successfully targeted, it should be rendered non-intrusive, reducing its recurrence to zero.^29^ Post-hoc mechanistic analyses showed that elimination of specific intrusive memories (measured the day after each ICTI use) was more likely when gameplay scores were higher and intrusive memory-recall vividness was lower, consistent with theory.^29^ Although exploratory, our findings align with retrieval-dependent interference models^27^ and highlight the value of further investigation of neurobiological and behavioural mechanisms of intrusive memories.

The Bayesian adaptive trial design enabled efficient evaluation with early stopping when convincing evidence was reached (n=99).^2^ Results replicate the findings of the optimisation trial GAINS-01 at week 4,^2^ and extend the findings of effectiveness to 24 weeks. GAINS-02 improves earlier work by adding a well matched active control group, since treatment as usual-only comparators often produce nocebo effects in psychotherapy trials.^17^ ICTI remained beneficial under recurrent trauma exposure and could be used again after experiencing new trauma. GAINS-01 focused on ICU staff, whereas GAINS-02 extended recruitment across NHS health-care staff, demonstrating generalisation. Overall, the GAINS trials show successful translation of this neuroscience-driven, intrusive memory-focused intervention^5^ from laboratory^30^ into real-world trauma settings (appendix p 101).

Our study has various limitations. One limitation is self-referral, which might introduce bias. However, self-referral could benefit accessibility in real-world settings by bypassing requirements for clinician-referral and reducing potential for stigma, as raised by health-care staff with lived experience of work-related trauma. The absence of diagnostic interviews limits determination of specific mental disorders, although this can help to avoid introduction of diagnostic barriers. Subgroup analyses for PTSD status were exploratory and intended to be hypothesis-generating; we explored this because of the substantial proportion of health-care staff working during the COVID-19 pandemic with high levels of post-traumatic stress and an interest in whether ICTI could work for both those with and without probable PTSD at baseline.^31^ Direct PTSD and non-PTSD comparisons are needed in future studies. Participants were mostly women. One further limitation is that the GAINS-02 population had a high proportion of White participants. Future work using larger samples should seek effectiveness and equity across diverse groups representative of the wider NHS workforce.

Both control groups probably benefited from using the daily intrusive memory diary, since symptom-tracking alone can improve outcomes. Two participants reported the diary burdensome. Although our exploratory analysis indicated no differential dropout between ICTI and active control groups, treatment as usual showed better retention, presumably reflecting lower demands, although ethical constraints prevented follow-up of withdrawn participants. Our post-hoc mechanistic findings align with previous predictions,^29^ but future studies should prespecify analyses and incorporate neurophysiological assessments.^32^, ^33^, ^34^ Testing additional populations who have experienced trauma (eg, hospital patients), exploring how self-referral pathways could increase treatment accessibility (eg, after sexual assault or cardiac arrest), and removing the guided sessions could also be tested in future work with the aim of aiding broader scalability. Although beneficial effects persisted at 6 months, even longer follow-ups should be studied. A larger scale clinical investigation testing how ICTI works in the real world and for diverse groups within the general population is warranted. We administered credibility ratings immediately before participants used the task in line with good practice.^22^ Future work could add later measurements, although this would probably be confounded with experience since intervention effects appeared from the first day of use.

Comparisons across PTSD digital therapy trials are challenging due to differing assessment methods, which can be difficult to align fairly. A 2021 Cochrane review of digital PTSD therapies found moderate benefits versus waitlist (*d*=0·61) but no superiority over other interventions.^35^ Subsequently, the digital STOP-PTSD study^23^ showed a 12-week therapist-assisted online trauma-focused cognitive behavioural therapy^4^ was superior to stress management (*d*=0·38 [95% CI 0·07–0·69]) at the primary endpoint (PCL-5 at 13 weeks after randomisation). In GAINS-02, the effect size for ICTI versus the active control was *d*=0·76 (0·21–1·32) for intrusive memories at the primary endpoint, and *d*=0·85 (0·36–1·33) in GAINS-01 (ICTI *vs* waitlist).^2^ For PCL-5 in GAINS-02, the week 4 effect size for ICTI versus the active control was *d*=0·94 (0·36–1·53) for the full sample and *d*=1·41 (0·45–2·37) for the small subgroup with probable PTSD at baseline. ICTI effects followed a single guided session without requirement for a qualified trauma therapist.

This trial shows that a brief neuroscience-derived, remotely delivered digital intervention (ICTI) can markedly reduce intrusive memories in health-care staff, despite ongoing trauma exposure. The beneficial effects were sustained over 24 weeks, and PTSD symptoms and general functioning also improved. ICTI was superior to a well matched active control, supporting specificity. Harnessing a popular computer game and removing the need for trauma-based disclosure might help to overcome barriers to seeking care post-trauma and increase compliance. The ICTI approach has been designed to target intrusive memories and is predicted to have a cascading effect on related PTSD symptoms. Since this hallmark symptom (imagery-based intrusive memories from a traumatic event) occurs across populations globally and arises from numerous types of trauma, the current results hold broad relevance.

### Contributors

### Data sharing

All data, code, and materials used in the analysis (excluding those containing demographic information to preserve participant anonymity) along with the study protocol, statistical analysis plan, and participant information sheet are available on the Open Science Framework (https://osf.io/cs6hn/).

## Declaration of interests

JK is a shareholder and director of P1vital Products, which is the study sponsor and manufacturer of i-spero and P1vital ePRO systems. ACB, AMar, OS, RDi, AMal, and RDa are employed by P1vital Products, and VR, LI, and ZI are previous employees of this company. RDi is a director and holds share options in P1vital Products. ACB and AMal hold share options in P1vital Products. JH is Co-Chair of Psychologists in Intensive Care UK and Chief Psychological Professions Officer at Gloucestershire Hospitals NHS Foundation Trust. CS's salary is partly funded by the National Institute for Health Research (NIHR133788) and Medical Research Council (MR/S035753/1 and MR/X005070/1). CS is Chair of the Experimental Medicine Panel (UK Research and Innovation–Medical Research Council) and gave evidence as an expert witness to the UK's national COVID inquiry. Her evidence included a summary of the published work on the impact of COVID 19 on the wellbeing of staff working in intensive care units during the pandemic. CS is a Trustee and non-executive member of the Board of Directors of the Science Media Centre UK (unpaid). TJ is supported by a grant from UK Medical Research Council (MC_UU_00002/14). EAH has received funding from the Wellcome Trust (223016/Z/21/Z), the Swedish Research Council (2020–00873), the Oak Foundation (OCAY-18–442), and Wellcome Leap. EAH is on the Board of Trustees of the MQ Foundation (unpaid). EAH developed the imagery-competing task intervention for intrusive memories and founded Afterimagery AB, a company registered in Sweden (holding ANEMONE™). EAH receives book royalties from Guildford Press and Oxford University Press and receives occasional honoraria for conference keynotes and clinical workshops. GMG is Chief Medical Officer at Compass pathways; holds shares and share options in Compass pathways PLC; and has served as consultant, advisor, or CME speaker in the past 5 years for Beckley Psytech, Boeringer Ingelheim, Clerkenwell Health, Compass pathways, Eli Lily, Evapharma, Janssen, Medscape, Novartis, Ocean Neuroscience, Servier, Signant Health, Sun pharma, and Takeda. All other authors declare no competing interests. The views expressed are those of the authors and not necessarily those of the National Health Service, the National Institute for Health and Care Research, or the Department of Health and Social Care.
