# Supplementary material for: A digital imagery-competing task intervention for stopping intrusive memories in trauma-exposed health-care staff during the COVID-19 pandemic in the UK: a Bayesian adaptive randomised clinical trial
Source: Lancet Psychiatry. 2026 Mar;13(3):233–47. doi: 10.1016/S2215-0366(25)00397-9 (PMC12916470; doi:10.1016/S2215-0366(25)00397-9)
Supplement: Supplementary appendix [file mmc1.pdf]

THE LANCET
Psychiatry

Supplementary appendix

This appendix formed part of the original submission and has been peer reviewed. We post it as supplied by the authors.

Supplement to: Beckenstrom AC, Bonsall MB, Markham A, et al. A digital imagery-competing task intervention for stopping intrusive memories in trauma-exposed health-care staff during the COVID-19 pandemic in the UK: a Bayesian adaptive randomised clinical trial. *Lancet Psychiatry* 2026; **13:** 233–47.

Supplementary Material

A brief, digital, imagery-competing task intervention for stopping intrusive memories in trauma-exposed health-care staff during the COVID-19 pandemic in the UK: a Bayesian adaptive randomised clinical trial.

Table of Contents:

Figure S1. Bayes factor progression at interim analyses 5

Table S1A. Weekly number of IMs at baseline, and weeks 4 (primary outcome), 12 and 24. 6

Table S1B. Weekly number of IMs at baseline, and weeks 4 (primary outcome), 12 and 24 by gender. 7

Table S2. Primary outcome: Bayesian model for reduction of IMs (at week 4). 9

Table S2A. Primary outcome: Bayesian model for reduction of IMs at week 4. Bayesian results of between group comparisons. 9

Table S2B. Primary outcome: Bayesian model for reduction of IMs (at week 4). Summary of the fitted Bayesian model. 11

Table S2C. Interpretation of Bayes factors 12

Figure S2. Primary outcome: Reduction in IMs. Convergence and goodness of fit, posterior density plots with varying priors, for (A) the Active Control parameter, and (B) the TaU parameter 13

Figure S3. Primary outcome: Reduction in IMs. Posterior trace and density plots. 14

Figure S4. Primary outcome: reduction in IMs. Posterior predictive checks (A) a density overlay plot comparing the observed data (dark line) with simulated datasets drawn from the posterior predictive distribution (light lines). (B) the empirical cumulative distribution function (ECDF) overlay, comparing the ECDF of the observed data (dark line) to those from posterior predictive simulations (light lines) 15

Table S3. Primary outcome: Sensitivity analysis, Bayesian model for reduction of IMs (at week 4) 17

Table S3A. Primary outcome: Sensitivity analysis, Bayesian model for reduction of IMs (at week 4) with complete case population/excluding imputed data 17

Table S3B. Primary outcome: Sensitivity analysis, Bayesian model for reduction of IMs at week 4 with outliers excluded 18

Figure S5. Primary outcome: Reduction in IMs. Sensitivity analyses, posterior density plots with and without outliers, for (A) the Active Control parameter, and (B) the TAU parameter 19

Table S4. Primary outcome: Sensitivity analyses, frequentist analysis of primary outcome 20

Table S4A. Frequentist analysis of primary outcome with incident rate ratios 20

Table S4B. Frequentist analysis of primary outcome with estimated Cohen's d effect size. 21

Table S5. Secondary outcomes: Intervention efficacy over time. Bayesian model for weekly number of IMs. 22

Table S5A. Secondary outcomes: Intervention efficacy over time. Bayesian model for weekly number of IMs. Summary of the fitted Bayesian model. 22

Table S5B. Secondary outcomes: Intervention efficacy over time. Bayesian model for weekly number of IMs. Bayesian results of between groups comparisons conducted at weeks 4, 12 and 24. 23

Table S5C. Post-hoc analysis. Benefits of ICTI on reduction of IMs from baseline estimated Cohen's d effect size. 24

Table S5D. Post-hoc analysis. Benefits of ICTI on IMs estimated Cohen's d effect size 25

Figure S6. Secondary outcome: Intervention efficacy on IMs sustained over time. Posterior trace and density plots. 26

Figure S7. Secondary outcome: Intervention efficacy on IMs sustained over time. Posterior predictive checks. (A) a density overlay plot comparing the observed data (dark line) with simulated datasets drawn from the posterior predictive distribution (light lines). (B) the empirical cumulative distribution function (ECDF) overlay, comparing the ECDF of the observed data (dark line) to those from posterior predictive simulations (light lines) 29

Figure S8. Secondary outcomes: Intervention efficacy on IMs sustained over time, Posterior density plots with varying priors 31

Table S6. Descriptive statistics for secondary outcomes 32

Table S7. Secondary outcomes: PTSD symptom severity at all time-points. Bayesian results of between groups comparisons 41

Figure S9. Secondary outcomes: PTSD symptom severity at all time-points. Sensitivity analysis with varying priors. 42

Figure S10A. Post-hoc analysis: PCL-5 subgroup sensitivity analysis with priors 43

Figure S10B. Post-hoc analysis: PTSD Symptom Severity (PCL-5 total) Across Time-Points per Arm and subgroup (with [baseline PCL-5 ≥33] or without [baseline PCL-5 <33] probable PTSD). 44

Table S8. Post-hoc analysis. Benefits of ICTI on PTSD symptom severity for those meeting criteria for PTSD. 45

Table S8A. Benefits of ICTI on PTSD symptom severity for subgroup without probable PTSD at baseline (<33 PCL-5). Bayesian results of between groups comparisons. 45

Table S8B. Benefits of ICTI on PTSD symptom severity for subgroup with probable PTSD at baseline (≥33 PCL-5). Bayesian results of between groups comparisons. 46

Table S8C. Post-hoc analysis. Benefits of ICTI on PTSD symptom severity overall and within PTSD subgroups and arms with estimated Cohen's d effect size. 47

Table S9. Post-hoc analysis. Benefits of ICTI on PTSD symptom severity for symptom clusters of PTSD. Bayesian results of between groups comparisons. 48

Table S9A. Benefits of ICTI on PTSD symptom severity for symptom clusters of PTSD: *Avoidance.* 48

Table S9B. Benefits of ICTI on PTSD symptom severity for symptom clusters of PTSD: *Re-experiencing.* 49

Table S9C. Benefits of ICTI on PTSD symptom severity for symptom clusters of PTSD: *Hyper-arousal* 50

Table S9D. Benefits of ICTI on PTSD symptom severity for symptom clusters of PTSD: *Negative alterations* 51

Table S10. Secondary outcomes: Other clinical outcomes, insomnia (SCI-2). Bayesian results of between groups comparisons. 52

Table S11. Secondary outcomes: Other clinical outcomes, anxiety (GAD-2). Bayesian results of between groups comparisons. 53

Table S12. Secondary outcomes: Other clinical outcomes, depression (PHQ-2). Bayesian results of between groups comparisons. 54

Table S13. Secondary outcomes: General functioning (WHODAS 12-item total score). Bayesian results of between groups comparisons 55

Table S14. Secondary outcomes: Quality of Life (EQ-5D-5L). Bayesian results of between groups comparisons 56

Table S14A. Secondary outcomes: Quality of Life (EQ-5D-5L), Mobility item. 56

Table S14B. Secondary outcomes: Quality of Life (EQ-5D-5L), Self-care item. 57

Table S14C. Secondary outcomes: Quality of Life (EQ-5D-5L), Usual activities item. 58

Table S14D. Secondary outcomes: Quality of Life (EQ-5D-5L), Pain-discomfort item. 59

Table S14E. Secondary Outcomes: Quality of Life (EQ-5D-5L), Anxiety-Depression Item. 60

Table S14F. Secondary outcomes: Quality of Life (EQ-5D-5L), Overall health (visual analogue scale) item. 61

Table S15. Secondary outcomes: Occupational outcomes, work engagement (SWEBO Engagement Subscale). Bayesian results of between groups comparisons 62

Table S15A. Secondary outcomes: Occupational outcomes, SWEBO Work Engagement subscale. 62

Table S15B. Secondary outcomes: Occupational outcomes, SWEBO work burnout subscale. 63

Table S16. Secondary outcomes: Occupational outcomes, Intention to leave job (ITL). Bayesian results of between groups comparisons 64

Table S17. Secondary outcomes: Occupational outcomes, Sickness absence. Bayesian results of between groups comparisons 65

Table S18. Secondary outcomes: Intrusive Memory Ratings (IMR). Bayesian results of between groups comparisons 66

Table S18A. IMR Q1. Approximately how often did IMs of the traumatic event pop into your mind? 66

Table S18B. IMR Q2. How distressing were your IMs? 67

Table S18C. IMR Q3. How much did they disrupt your concentration? 68

Table S18D. IMR Q4. How much did they interfere with what you were doing? 69

Table S18E. IMR Q4a. And for how long each time (approximately)? 70

Table S18F. IMR Q5. How much did your IMs affect your work functioning? 71

Table S18G. IMR Q7. How much did your IMs affect your functioning in other areas of your life (e.g. relationships with other people, parenting, social life, study, housework, voluntary work etc.) 72

Table S19. Other outcomes. Intervention feedback questionnaire (IFQ). Descriptive summary statistics. 73

Table S20: Other outcome measures. Feedback questionnaire. Bayesian results of between groups comparisons 75

Table S20A. How easy did you find it to use the brief cognitive task? 75

Table S20B. How helpful did you find the brief cognitive task? 75

Table S20C. How burdensome did you find the brief cognitive task? 75

Table S20D. How distressing did you find the brief cognitive task? 75

Table S20E. Overall, how acceptable did you find the brief cognitive task? 76

Table S20F. If you were having intrusive memories in the future, how willing would you be to use the brief cognitive task if it was offered to you as something that would help? 76

Table S20G. If a colleague or friend was having intrusive memories, how confident would you be in recommending the brief cognitive task to them? 76

Table S20H. How much do you feel that this brief cognitive task could be used within NHS Trusts/healthcare organisations to support staff who have experienced work-related traumatic events? 76

Table S21: Other outcomes. ICTI/AC Self-guided usage. Descriptive summary statistics. 77

Table S22. Other outcomes. Changes to health and work. Descriptive summary statistics 78

Table S23A. Other outcomes. Credibility and expectancy of the intervention. Bayesian results of between groups comparisons of individual items 82

Table S23A.i. CEQ Item 1. At this point, how logical does the intervention offered to you seem? 82

Table S23A.ii. CEQ Item 2. At this point, how successful do you think this intervention will be in reducing your IMs? 82

Table S23A.iii. CEQ Item 3. How confident would you be in recommending this intervention to a friend who experiences similar problems? 82

Table S23A.iv. CEQ Item 4. By the end of the intervention period (four weeks), how much improvement in your IMs do you think will occur? 83

Table S23A.v. CEQ Item 5. At this point, how much do you really feel that the intervention will help you to reduce your IMs?........ 83

Table S23A.vi. CEQ Item 6. By the end of the intervention period (four weeks), how much improvement in your IMs do you really feel will occur? 83

Table S23B. Post-hoc Analysis. Total Score of Credibility and Expectancy (CEQ). Bayesian results of between groups comparisons........ 84

Table S24. Adverse Events, Serious Adverse Events and Concomitant Treatments Started During the In-Study Period........ 85

Table S24A. Table of Adverse Events 85

Table S24B. Table of serious adverse events........ 88

Table S24C. Table of Concomitant Treatments Started During the In-Study Period........ 89

Table S25A. Post-hoc analysis: Participant retention at primary outcome per arm. Bayesian logistic regression 90

Table S25B. Post-hoc analysis: Participant retention at primary outcome. Baseline characteristics for intention to treat (ITT) population split by data missingness at primary outcome, between groups. 91

Table S25C. Post-hoc analysis: Participant retention at primary outcome. Baseline characteristics for intention to treat (ITT) population split by data missingness at primary outcome, across groups. 93

Table S26. Imputed IM daily diary missing data (weeks 4, 12 and 24) 94

Figure S11. Primary outcome. Primary outcome histograms. 95

Supplementary Methods 1. Task Adherence for Imagery-Competing Task Intervention and Music-Listening Active Control Task 96

Supplementary Methods 2. Training of Digital Navigators to Conduct the Guided Session 96

Supplementary Methods 3. Modelling the total number of IMs recorded in week-4........ 97

Supplementary Methods 4. Imputation, Missing Data and Outliers........ 98

Supplementary Methods 5. Analysis of secondary, exploratory (not pre-specified) and mechanistic (not pre-specified) outcomes........ 99

Supplementary Methods 6. Further considerations in choosing intrusive memories as the treatment target 101

Supplementary Methods 7. Ethnicity data collection and analysis 102

References for Secondary Outcome Measures........ 103

Figure S12. Study Schematic and Follow-up Time Points. 104

Figure S13. Number of intrusive memories over time per group. 105

References........ 106

Figure S1. Bayes factor progression at interim analyses

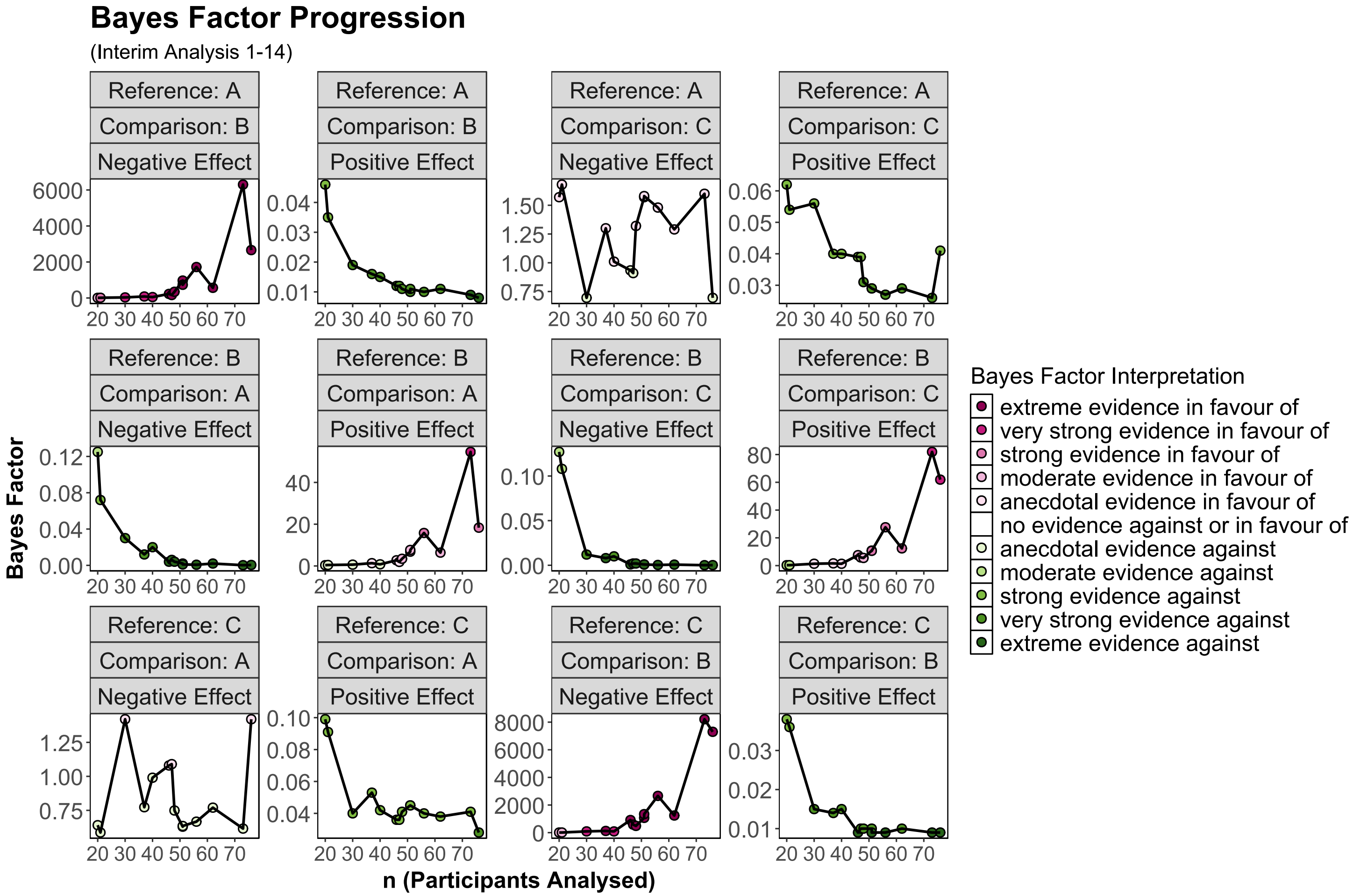

Note. A = TAU; B = ICTI; C = AC. In response to a helpful reviewer comment, we note the Bayes Factor progression shows a drop in evidence at the final analysis point. This likely reflects the variability in the additional participants, and we note that the overall evidence remained strong. If recruitment had continued, additional posterior updates would likely average out the variability keeping the Bayes Factor in a range of strong evidence.

Table S1A. Weekly number of IMs at baseline, and weeks 4 (primary outcome), 12 and 24.

	Baseline				Week4				Week12				Week24			
**Variable**	**All**	**TAU**	**AC**	**ICTI**	**All**	**TAU**	**AC**	**ICTI**	**All**	**TAU**	**AC**	**ICTI**	**All**	**TAU**	**AC**	**ICTI**
**N**	99	20	39	40	76	19	31	26	68	19	26	23	68	19	24	25
Mean (SD)	14.31 (12.74)	14.05 (16.52)	13.03 (9.58)	15.70 (13.48)	6.09 (6.87)	7.32 (6.61)	7.87 (7.84)	3.08 (4.66)	4.82 (7.38)	5.84 (8.39)	6.54 (8.47)	2.04 (3.77)	3.94 (5.84)	6.79 (8.07)	4.79 (5.38)	0.96 (1.51)
Median (IQR)	10.00 (6.50 - 17.50)	6.50 (5.00 - 17.75)	9.00 (6.50 - 16.00)	11.50 (9.00 - 18.00)	4.00 (1.00 - 7.25)	5.00 (2.50 - 8.00)	5.00 (3.00 - 11.50)	0.50 (0.00 - 5.00)	2.00 (0.00 - 6.25)	3.00 (0.00 - 6.50)	4.50 (1.25 - 8.00)	0.00 (0.00 - 2.00)	2.00 (0.00 - 4.00)	3.00 (1.00 - 11.50)	3.00 (1.75 - 5.25)	0.00 (0.00 - 1.00)
Min - Max	3 - 84	4 - 75	3 - 37	4 - 84	0 - 32	0 - 23	0 - 32	0 - 16	0 - 37	0 - 29	0 - 37	0 - 12	0 - 24	0 - 24	0 - 23	0 - 5

Note. The number of IMs recorded in the weekly IM diary at baseline, and weeks, 4, 12 and 24 is presented including imputed data. Means (SD), medians (IQR) and ranges are presented for weekly total counts of intrusive memories at each time-point.

Abbreviations. SD = standard deviation; IQR = interquartile range; ICTI = Imagery-Competing Task Intervention; IM = intrusive memory; AC = active control; TAU = treatment-as-usual.

Table S1B. Weekly number of IMs at baseline, and weeks 4 (primary outcome), 12 and 24 by gender.

	Baseline				Week4				Week12				Week24			
**Variable**	**All**	**TAU**	**AC**	**ICTI**	**All**	**TAU**	**AC**	**ICTI**	**All**	**TAU**	**AC**	**ICTI**	**All**	**TAU**	**AC**	**ICTI**
Gender = Woman																
**N**	85	18	33	34	64	17	26	21	59	17	23	19	59	17	22	20
Mean (SD)	15.00 (13.40)	15.00 (17.19)	13.79 (10.15)	16.18 (14.21)	6.50 (7.24)	7.76 (6.81)	8.62 (8.24)	2.86 (4.69)	5.00 (7.79)	6.29 (8.78)	6.70 (8.97)	1.79 (3.52)	3.90 (5.67)	6.06 (7.44)	4.91 (5.61)	0.95 (1.50)
Median (IQR)	11.00 (7.00 – 18.00)	7.50 (5.00 - 19.25)	9.00 (7.00 - 18.00)	12.00 (9.00 - 17.75)	4.00 (1.75 - 8.00)	5.00 (3.00 - 8.00)	5.00 (3.00 - 13.00)	0.00 (0.00 - 5.00)	2.00 (0.00 - 6.50)	3.00 (0.00 - 7.00)	5.00 (0.50 - 8.00)	0.00 (0.00 - 1.50)	2.00 (0.00 - 4.00)	3.00 (0.00 - 9.00)	3.00 (1.25 - 5.75)	0.00 (0.00 - 1.25)
Min - Max	3 - 84	4 - 75	3 - 37	4 - 84	0 - 32	0 - 23	0 - 32	0 - 16	0 - 37	0 - 29	0 - 37	0 - 12	0 - 24	0 - 24	0 - 23	0 - 5
Gender = Man																
**N**	12	2	6	4	10	2	5	3	7	2	3	2	7	2	2	3
Mean (SD)	9.08 (4.03)	5.50 (0.71)	8.83 (3.60)	11.25 (4.79)	3.50 (3.10)	3.50 (3.54)	4.00 (3.81)	2.67 (2.52)	2.86 (3.13)	2.00 (1.41)	5.33 (3.21)	0.00 (0.00)	4.86 (8.15)	13.00 (14.14)	3.50 (0.71)	0.33 (0.58)
Median (IQR)	8.50 (6.00 - 11.00)	5.50 (5.25 - 5.75)	9.00 (6.50 - 10.75)	10.00 (8.50 - 12.75)	3.00 (1.25 - 5.00)	3.50 (2.25 - 4.75)	3.00 (2.00 - 5.00)	3.00 (1.50 - 4.00)	3.00 (0.50 - 3.50)	2.00 (1.50 - 2.50)	4.00 (3.50 - 6.50)	0.00 (0.00 - 0.00)	3.00 (0.50 - 3.50)	13.00 (8.00 - 18.00)	3.50 (3.25 - 3.75)	0.00 (0.00 - 0.50)

Min - Max	4 - 18	5 - 6	4 - 14	7 - 18	0 - 10	1 - 6	0 - 10	0 - 5	0 - 9	1 - 3	3 - 9	0 - 0	0 - 23	3 – 23	3 - 4	0 - 1
Gender = Non-binary or prefer not to say[a]																
**N**	2				2				2				2			
Mean	16.5				6				6.5				2			
Median	16.5				6				6.5				2			

Note. The number of IMs recorded in the weekly IM diary by participants recording their gender identity as ‘woman’, ‘man’ or ‘non-binary or prefer not to say’ is presented including imputed data at baseline, and weeks, 4, 12 and 24. Means (SD), medians (IQR) and ranges are presented for weekly total counts of intrusive memories at each time-point. [a]Since n=2, individual arms and further summary statistics are not presented to avoid potential identification of participants through the derivation of individual data points.

Abbreviations. SD = standard deviation; IQR = interquartile range; ICTI = Imagery-Competing Task Intervention; IM = intrusive memory; AC = active control; TAU = treatment-as-usual.

Table S2. Primary outcome: Bayesian model for reduction of IMs (at week 4).

The primary endpoint – number of IMs of traumatic events recorded in the IM diary at Week 4 controlling for baseline – was modelled using a Poisson linear mixed model with OLRE. In the model, the baseline number of IMs and treatment arms were fitted as fixed effects with a random intercept effect for Participant. The model consists of two categorical treatment assignment parameters providing a comparison to the AC and TAU arms with the ICTI arm taken as the reference group.

- Table S2A presents the Bayesian results of between groups comparisons conducted at week 4 testing for differences between the number of IMs recorded by the ICTI group relative to the AC and TAU groups.
- Table S2B presents the summary of the fitted Bayesian model for reduction of IMs at week 4
- Table S2C presents an interpretation of Bayes factors for Bayes factor boundaries of evidence towards hypotheses.

All analyses were completed in R version 4.1.2[1] on an intention-to-treat basis.

Table S2A. Primary outcome: Bayesian model for reduction of IMs at week 4. Bayesian results of between group comparisons.

Timepoint	Ref. Group	Ref. N	Comp. Group	Comp. N	Ref. Mean (SD)	Comp. Mean (SD)	Comp. vs Ref. Estimate	Lower 95% CrI	Upper 95% CrI	BF +ve treatment effect	BF -ve treatment effect	*Pr* (ICTI < Comp.)
Baseline	ICTI	26	AC	31	13.692 (7.989)	12.452 (10.019)						
Baseline	ICTI	26	TAU	19	13.692 (7.989)	14.474 (16.860)						
Week 4	ICTI	26	AC	31	3.077 (4.664)	7.871 (7.839)	1.293	0.639	1.997	114.1006	<0.0001	>0.99
Week 4	ICTI	26	TAU	19	3.077 (4.664)	7.316 (6.609)	1.215	0.488	1.982	15.8033	0.0006	>0.99

Note. Bayes factors (BF) quantifying the evidence for an alternative hypothesis (H_1) over the null hypothesis (H_0) are presented to illustrate evidence of positive and negative treatment effects of the ICTI. The ICTI arm is treated as the reference group with the AC and TAU arms as comparators. The estimate is the difference in the log expected count of the outcome between the comparator and reference arms at week 4. Means (SD) for the end analysis population are presented for each group at both time-points (baseline and week 4) such that only data used in the Bayesian model (subjects with data present for both baseline and week 4, including imputed data) are included in the descriptive summaries presented in the table. *Pr*(ICTI<Comp.) is the estimated posterior probability of fewer IMs in ICTI than the Comp Group (*post-hoc* analysis).

The posterior means [95% credible interval] for the primary comparisons between ICTI and AC and TAU arms respectively were 1.29 [0.64, 2.00] and 1.21 [0.49, 1.98]. This represents the change compared to ICTI on a log-scale such that, controlling for baseline number of IMs, the expected log number of IMs would be 1.293 and 1.215 fewer in ICTI than in the AC and TAU arms respectively. After back-transforming from the log-scale, this is equivalent to 0.28 (from 1/e^1.29) and 0.30 (from 1/e^1.21) times as many IMs in ICTI than in the AC and TAU arms, respectively.

Abbreviations: ICTI = Imagery-Competing Task Intervention; AC = active control; TAU = treatment-as-usual; IM = intrusive memory; Ref. = reference [group]; Comp. = comparator [group]; SD = standard deviation; CrI = credible interval; BF = Bayes factor; +/-ve = positive/negative.

Table S2B. Primary outcome: Bayesian model for reduction of IMs (at week 4). Summary of the fitted Bayesian model.

Parameter	Estimate	Est.Error	l-95% CrI	u-95% CrI	Rhat	Bulk_ESS	Tail_ESS
sd(Intercept)	1.04	0.14	0.8	1.33	1	30,048	52,554
Intercept	-0.11	0.33	-0.8	0.51	1	39,332	57,261
PO_imp_Baseline	0.03	0.01	0.01	0.06	1	37,163	60,128
ARMActiveControl	1.29	0.35	0.64	2	1	33,035	51,356
ARMTaU	1.21	0.38	0.49	1.98	1	34,123	52,383

Note. Each row corresponds to a parameter, an estimated value (estimate) and the standard error of this estimated value. The 95% credible interval (CrI) shows the lower and upper bounds of the range containing the true value of each parameter. Rhat values of 1 show successful convergence of the model. Finally, the estimated sample sizes (ESS), Bulk ESS and Tail ESS, demonstrate the reliability of estimates and credible intervals and that sufficient chains were used in model generation.

Abbreviations. sd(Intercept) = standard deviation of random intercept; PO_imp = Primary Outcome, Imputed; Est.Error = standard error of estimate; L-95% CI = lower bound of 95% credible interval; U-95% CI = upper bound of 95% credible interval; Rhat = convergence diagnostic; ESS = effective sample size; IM = intrusive memory; TAU = treatment-as-usual

Table S2C. Interpretation of Bayes factors

A heuristic classification scheme for Bayes factors.

BF	**Interpretation**
>100	Extreme evidence towards H_1
30-100	Very strong evidence towards H_1
10-30	Strong evidence towards H_1
3-10	Moderate evidence towards H_1
1-3	Anecdotal evidence towards H_1
1	No evidence
1-1/3	Anecdotal evidence towards H_0
1/3 – 1/10	Moderate evidence towards H_0
1/10 – 1/30	Strong evidence towards H_0
1/30 – 1/100	Very strong evidence towards H_0
< 1/100	Extreme evidence towards H_0

Note. Guidelines by Lee & Wagenmakers[2] for Bayes factor boundaries of evidence towards hypotheses. These boundaries are guidelines only and cut-offs do not reflect significance of evidence. For primary analysis, H_1 refers to the posterior of the model (i.e., the hypothesis that the ICTI is associated with a positive or negative treatment effect) and H_0 is the prior of the model (i.e., that there is no difference between the groups) with BFs representing the ratios of the proportions of the posterior distribution in the direction of the hypothesis. However, for secondary analyses, hypotheses were constructed such that H_1 is the alternative hypothesis indicating that one group (ref or comp) is greater than the other group (ref or comp).

Figure S2. Primary outcome: Reduction in IMs. Convergence and goodness of fit, posterior density plots with varying priors, for (A) the Active Control parameter, and (B) the TaU parameter

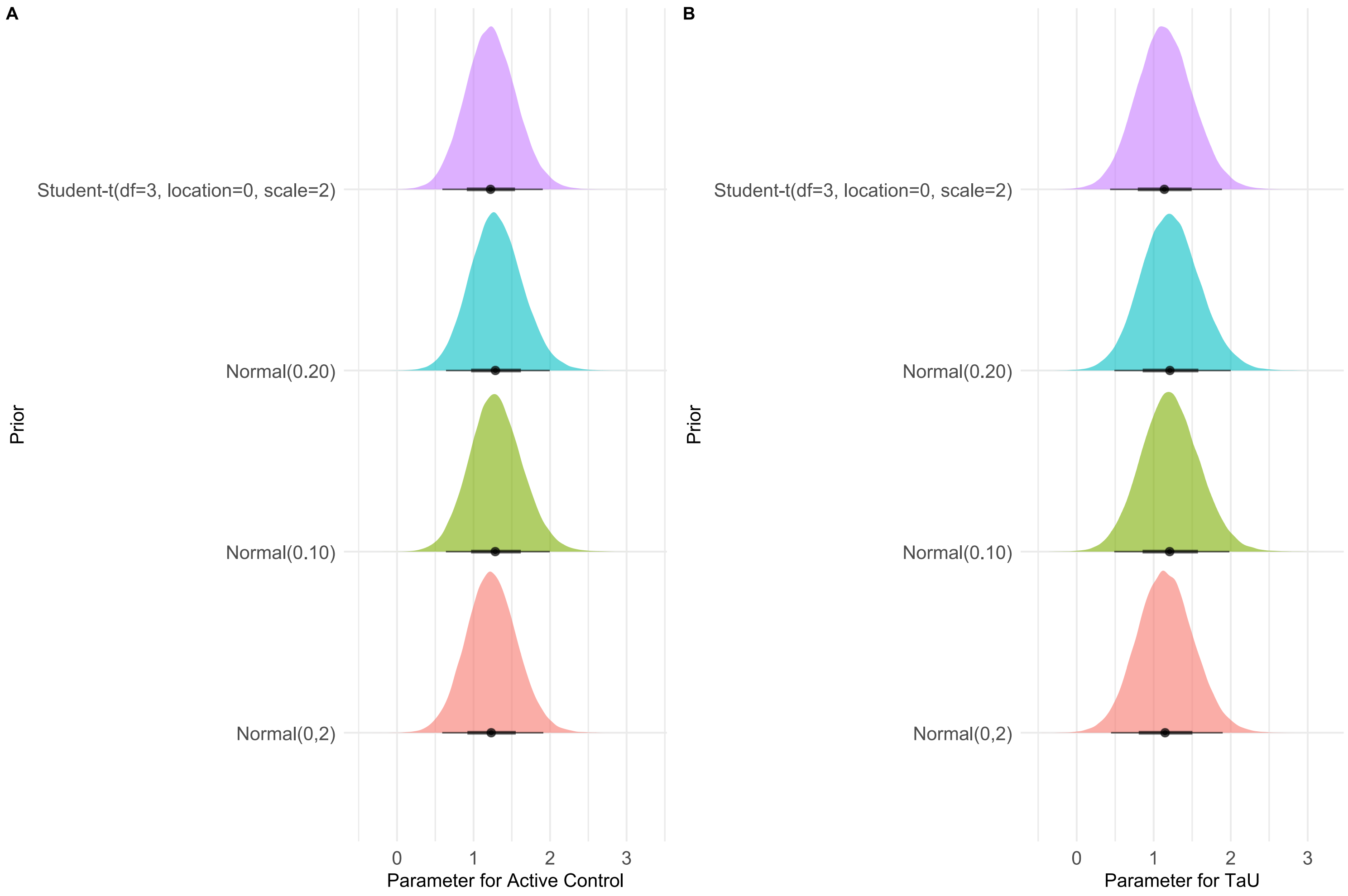

Note. For all model checks see OSF: P1V-GAINS-IN02_Final-Exploratory-Modelling-PrimaryOutcome_OSF.html

Figure S3. Primary outcome: Reduction in IMs. Posterior trace and density plots.

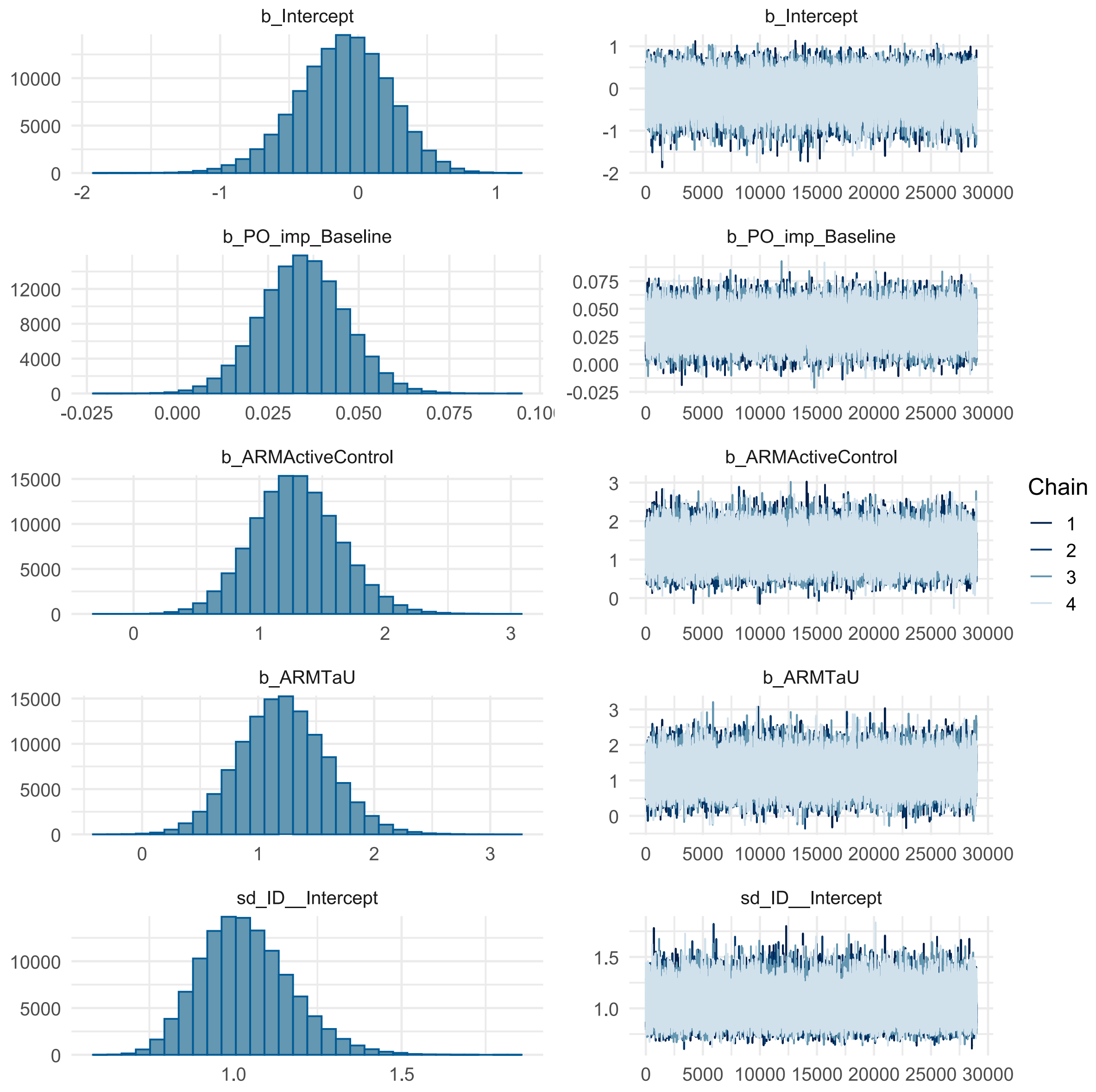

Figure S4. Primary outcome: reduction in IMs. Posterior predictive checks (A) a density overlay plot comparing the observed data (dark line) with simulated datasets drawn from the posterior predictive distribution (light lines). (B) the empirical cumulative distribution function (ECDF) overlay, comparing the ECDF of the observed data (dark line) to those from posterior predictive simulations (light lines)

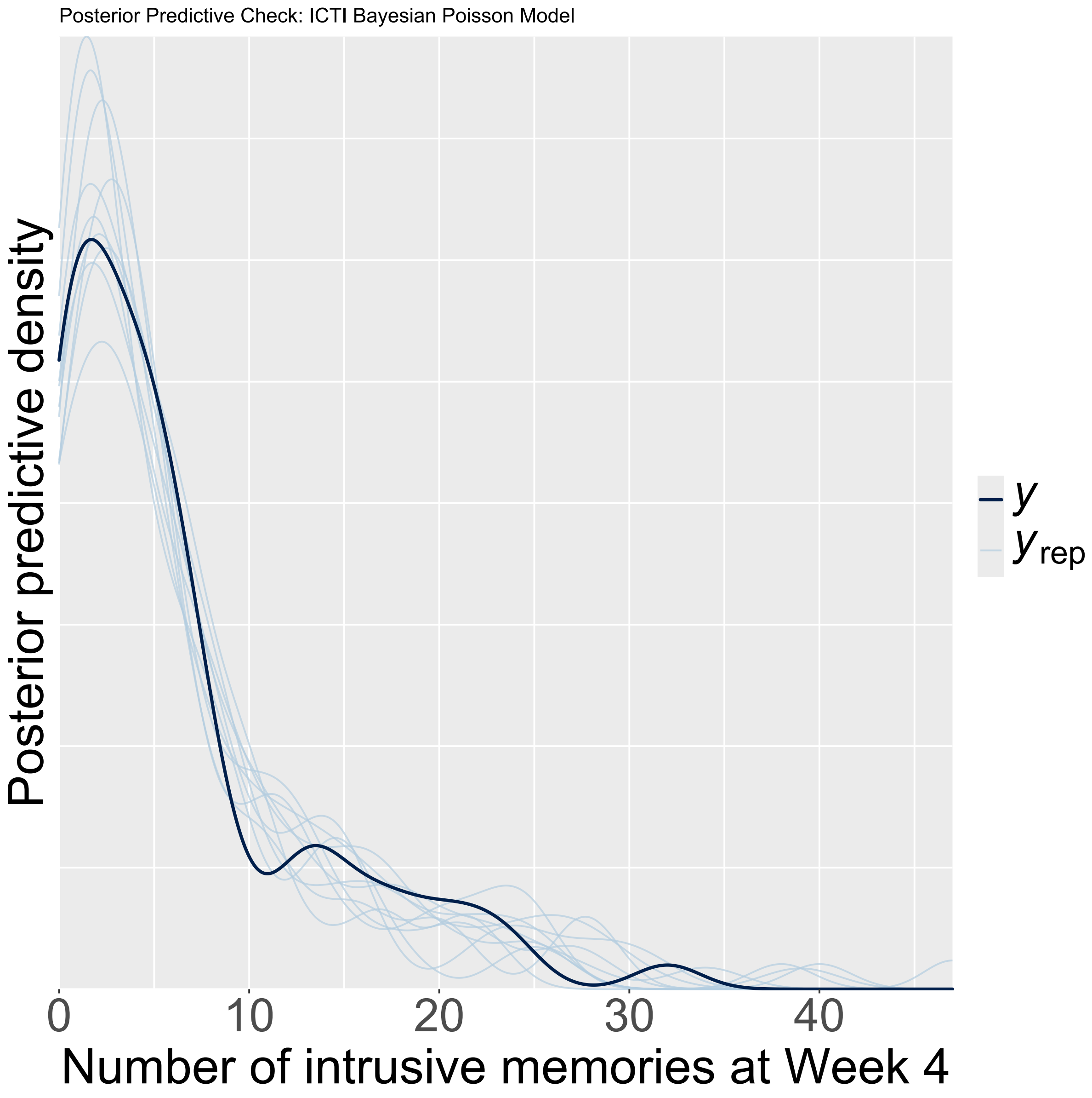

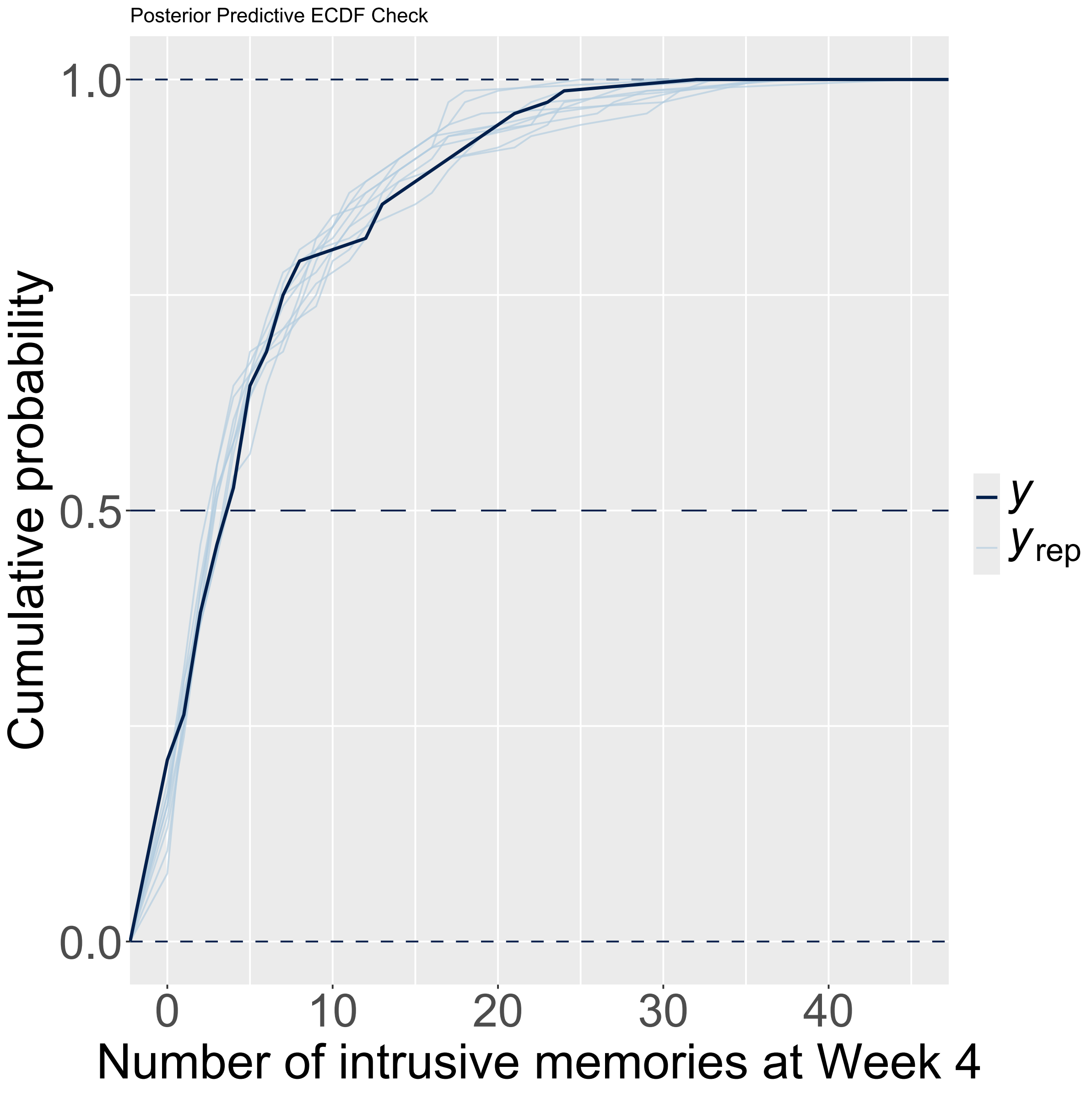
Posterior Predictive ECDF Check
Cumulative probability
1.0
0.5
0.0
0
10
20
30
40
Number of intrusive memories at Week 4
y
y rep

Table S3. Primary outcome: Sensitivity analysis, Bayesian model for reduction of IMs (at week 4)

Table S3A. Primary outcome: Sensitivity analysis, Bayesian model for reduction of IMs (at week 4) with complete case population/excluding imputed data

Timepoint	Ref. Group	Ref. N	Comp. Group	Comp. N	Ref. Mean (SD)	Comp. Mean (SD)	Comp. vs Ref. Estimate	Lower 95% CrI	Upper 95% CrI	BF +ve treatment effect	BF -ve treatment effect
Baseline	ICTI	26	AC	30	13.692 (7.989)	12.667 (10.118)					
Baseline	ICTI	26	TAU	19	13.692 (7.989)	14.474 (16.860)					
Week 4	ICTI	26	AC	30	3.077 (4.664)	8.067 (7.896)	1.322	0.665	2.024	112.7084	<0.0001
Week 4	ICTI	26	TAU	19	3.077 (4.664)	7.316 (6.609)	1.221	0.488	1.999	14.5245	0.0006

Note. Bayes factors (BF) quantifying the evidence for an alternative hypothesis (H_1) over the null hypothesis (H_0) are presented to illustrate evidence in favour of positive and negative treatment effects of the ICTI. The model is the same as was used for the primary analysis but here is conducted on the end analysis population with n=1 participant from the AC arm removed [imputed data]. The ICTI arm is treated as the reference group with the AC and TAU arms treated as the comparators. The estimate is the difference in the log expected count of the outcome between the comparator and reference arms at week 4.

Abbreviations: ICTI = Imagery-Competing Task Intervention; AC = active control; TAU = treatment-as-usual; Ref. = reference [group]; Comp. = comparator [group]; SD = standard deviation; CrI = credible interval; BF = Bayes factor; +/-ve = positive/negative.

Table S3B. Primary outcome: Sensitivity analysis, Bayesian model for reduction of IMs at week 4 with outliers excluded

Timepoint	Ref. Group	Ref. N	Comp. Group	Comp. N	Ref. Mean (SD)	Comp. Mean (SD)	Comp. vs Ref. Estimate	Lower 95% CrI	Upper 95% CrI	BF +ve treatment effect	BF -ve treatment effect
Baseline	ICTI	26	AC	31	13.692 (7.989)	12.452 (10.019)					
Baseline	ICTI	26	TAU	18	13.692 (7.989)	11.111 (8.574)					
Week 4	ICTI	26	AC	31	3.077 (4.664)	7.871 (7.839)	1.279	0.665	1.942	172.4663	<0.0001
Week 4	ICTI	26	TAU	18	3.077 (4.664)	7.444 (6.776)	1.372	0.679	2.114	126.2355	<0.0001

Note. Bayes factors (BF) quantifying the evidence for an alternative hypothesis (H_1) over the null hypothesis (H_0) are presented to illustrate evidence in favour of positive and negative treatment effects of the ICTI. The model is the same as was used for the primary analysis but here is conducted on the end analysis population with n=1 participant from the TAU arm removed after being identified as an outlier through inspection of residual plots and Cook's distance vs leverage plots of an appropriate fitted frequentist model. The ICTI arm is treated as the reference group with the AC and TAU arms treated as the comparators. The estimate is the difference in the log expected count of the outcome between the comparator and reference arms at week 4.

Abbreviations: ICTI = Imagery-Competing Task Intervention; AC = active control; TAU = treatment-as-usual; IM = intrusive memory; Ref. = reference [group]; Comp. = comparator [group]; SD = standard deviation; CrI = credible interval; BF = Bayes factor; +/-ve = positive/negative.

Figure S5. Primary outcome: Reduction in IMs. Sensitivity analyses, posterior density plots with and without outliers, for (A) the Active Control parameter, and (B) the TAU parameter

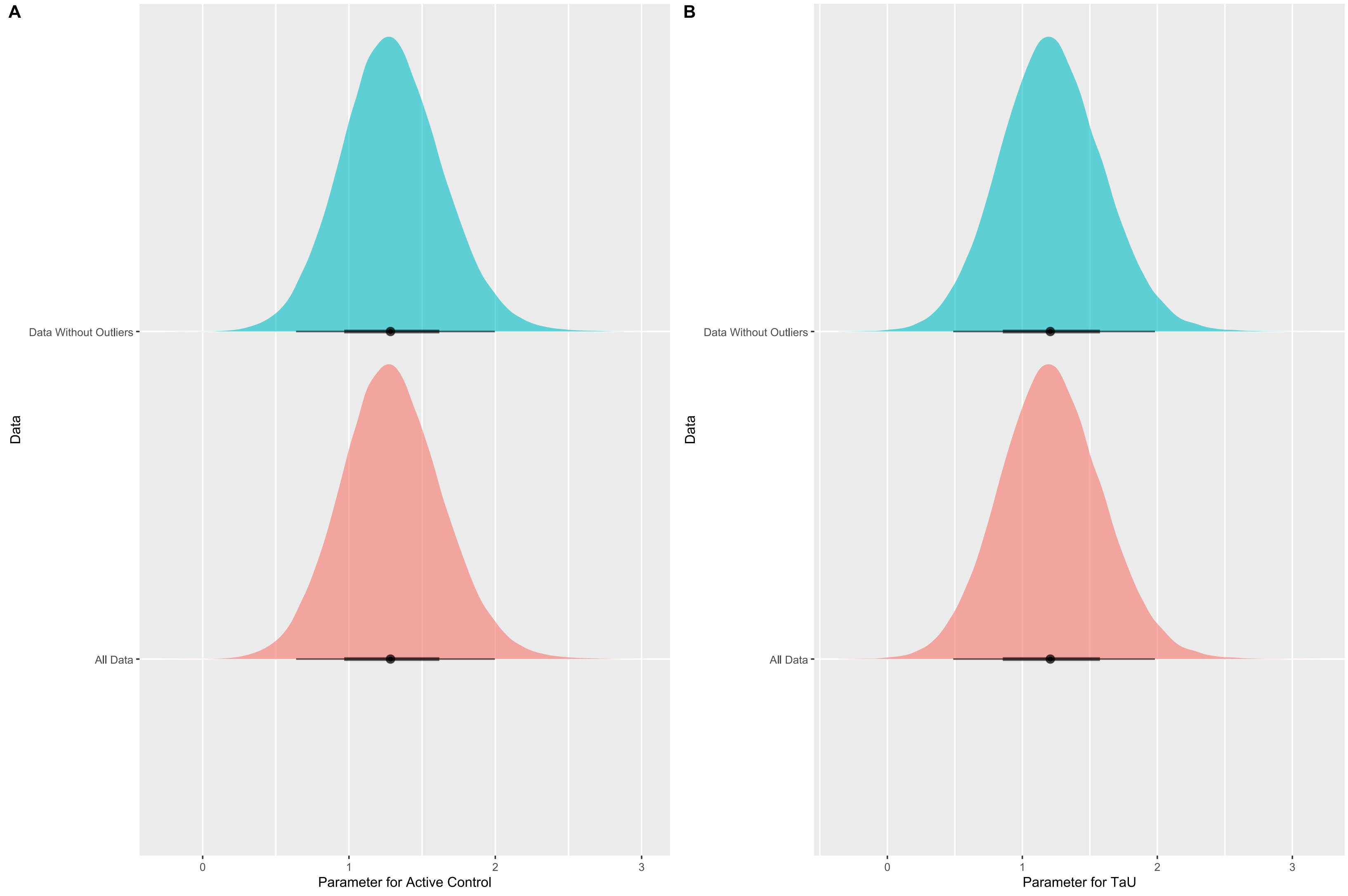

Table S4. Primary outcome: Sensitivity analyses, frequentist analysis of primary outcome

Table S4A. Frequentist analysis of primary outcome with incident rate ratios

Parameter	IRR	p-value	Lower 95% CI IRR	Upper 95% CI IRR
**ARM-AC**	3.48	<0.0001	1.86	6.49
**ARM-TAU**	3.22	0.001	1.62	6.43

Notes. For the primary outcome, frequentist statistical testing was performed as a sensitivity analysis. The model utilised for the frequentist analysis of the primary outcome is the same as described for the Bayesian primary analysis with baseline intrusive memories and treatment arm as fixed effects, and subject as a random effect. The same primary outcome data was used for this modelling.

Additionally, Incidence Rate Ratios (IRRs), and their 95% confidence intervals, are reported for (i) the intervention group vs. active control group comparison and (ii), the intervention group vs. TaU group comparison. The IRR represents the relative rate of occurrence of IMs compared to the reference level (i.e., the ICTI arm) or the comparator (e.g., AC or TAU arms).

Abbreviations. AC = active control arm; TAU = treatment-as-usual; IRR = incident rate ratio; CI = confidence interval.

Table S4B. Frequentist analysis of primary outcome with estimated Cohen's d effect size.

Comparison	Cohen's d	Lower 95% CI	Upper 95% CI
**ICTI vs AC**	0.76	0.21	1.32
**ICTI vs TAU (outlier included)**	0.27	-0.34	0.88
**ICTI vs TAU (outlier removed)**	0.85	0.20	1.49

Note. For compatibility with prior research, we calculated the estimated Cohen's d effect size of reduction in number of intrusive memories primary endpoint as a post-hoc analysis. For the ICTI group compared to the ACs the effect was moderate-large, 0.76 (95% CI 0.21-1.32), but the estimated effect size was smaller relative to TAU (Cohen's d=0.27; 95% CI -0.34-0.88). On inspection, an outlier in the TAU arm was identified with high residual, leverage, and influence values and was excluded. Excluding this data point, the effect of the ICTI relative to TAU was large (estimated Cohen's d=0.85; 95% CI 0.20-1.49).

Abbreviations. ICTI = Imagery-Competing Task Intervention arm; AC = active control arm; TAU = treatment-as-usual; IM = intrusive memory; CI = confidence interval.

Table S5. Secondary outcomes: Intervention efficacy over time. Bayesian model for weekly number of IMs.

Secondary analysis of the number of intrusive memories recorded in the intrusive memory diary at weeks 12 and 24 was modelled using a negative binomial model with a log link function. However, this model includes all follow-up time points (i.e., weeks 4, 12 and 24) in addition to baseline.

Table S5A. Secondary outcomes: Intervention efficacy over time. Bayesian model for weekly number of IMs. Summary of the fitted Bayesian model.

Parameter	Estimate	Est.Error	l-95% CrI	u-95% CrI	Rhat	Bulk_ESS	Tail_ESS
Intercept	0.07	0.35	-0.63	0.75	1.00	56,902	76,182
Baseline	0.03	0.01	0.00	0.05	1.00	64,946	81,022
VISITWeek12	-0.56	0.33	-1.20	0.08	1.00	78,173	87,709
VISITWeek24	-1.17	0.35	-1.86	-0.49	1.00	75,425	85,919
ARMActiveControl	1.34	0.39	0.60	2.12	1.00	56,334	72,793
ARMTaU	1.43	0.43	0.60	2.30	1.00	53,352	72,706
VISITWeek12:ARMActiveControl	0.31	0.40	-0.48	1.11	1.00	84,086	86,765
VISITWeek24:ARMActiveControl	0.72	0.43	-0.12	1.57	1.00	81,169	90,145
VISITWeek12:ARMTaU	-0.11	0.44	-0.98	0.75	1.00	85,187	91,227
VISITWeek24:ARMTaU	0.75	0.46	-0.14	1.65	1.00	81,379	87,8814

Note. Each row corresponds to a parameter, an estimated value (estimate) and the standard error of this estimated value. The 95% CI shows the lower and upper bounds of the range containing the true value of each parameter. Rhat values of 1 show successful convergence of the model. Finally, the estimated sample sizes (ESS), Bulk_ESS and Tail_ESS, demonstrate the reliability of estimates and credible intervals and that sufficient chains were used in model generation.

Abbreviations. SD = standard deviation of random intercept; Est.Error = standard error of estimate; L-95% CI = lower bound of 95% credible interval; U-95% CI = upper bound of 95% credible interval; Rhat = convergence diagnostic; ESS = effective sample size; IM = intrusive memory; TAU = treatment-as-usual

Table S5B. Secondary outcomes: Intervention efficacy over time. Bayesian model for weekly number of IMs. Bayesian results of between groups comparisons conducted at weeks 4, 12 and 24.

Timepoint	Ref. Group	Ref. N	Comp. Group	Comp. N	Ref. Mean (SD)	Comp. Mean (SD)	Comp. vs Ref. Estimate	Lower 95% CrI	Upper 95% CrI	BF (ICTI < Comp)	BF (ICTI > Comp)	*Pr* (ICTI < Comp.)
Baseline	ICTI	27	AC	33	13.815 (7.860)	13.030 (10.126)						
Baseline	ICTI	27	TAU	20	13.815 (7.860)	14.050 (16.519)						
Week 4	ICTI	26	AC	31	3.077 (4.664)	7.871 (7.839)	1.343	0.602	2.120	5042.4783	0.0002	> 0.99
Week 4	ICTI	26	TAU	19	3.077 (4.664)	7.316 (6.609)	1.434	0.604	2.299	3740.9355	0.0003	> 0.99
Week 12	ICTI	23	AC	26	2.043 (3.772)	6.538 (8.472)	1.656	0.834	2.509	23199.0000	<0.0001	> 0.99
Week 12	ICTI	23	TAU	19	2.043 (3.772)	5.842 (8.395)	1.321	0.429	2.239	541.0561	0.0018	> 0.99
Week 24	ICTI	25	AC	24	0.960 (1.513)	4.792 (5.381)	2.068	1.198	2.974	Inf	<0.0001	> 0.99
Week 24	ICTI	25	TAU	19	0.960 (1.513)	6.789 (8.066)	2.187	1.265	3.140	Inf	<0.0001	> 0.99

Note. Bayes factors quantify the evidence for an alternative hypothesis (H_1) over the null hypothesis (H_0) and are presented to illustrate evidence in favour of the ICTI group having greater or fewer IMs than the comparator arm, in contrast to the primary outcome model which illustrates evidence of the model having an effect relative to the prior. This model includes fixed effects for week (week 4, 12, 24) and for interactions between treatment arm and week. Bayes factors for between-groups analysis at week 4 will differ to the primary analysis (which included only week 4 as a fixed parameter). Means (SD) for the end analysis population are presented for each group at all time-points such that only data used in the Bayesian model (including imputed data) is included in the descriptive summaries presented in the table. Pr(ICTI<Comp.) is the estimated posterior probability of fewer IMs in ICTI than the Comp Group (*post-hoc* analysis).

Abbreviations: ICTI = Imagery-Competing Task Intervention; AC = active control; TAU = treatment-as-usual; IM = intrusive memory; Ref. = reference [group]; Comp. = comparator [group]; SD = standard deviation; CrI = credible interval; BF = Bayes factor.

Table S5C. Post-hoc analysis. Benefits of ICTI on reduction of IMs from baseline estimated Cohen's d effect size.

Comparison	Week	Cohen's d	Lower 95% CI	Upper 95% CI
**Outlier excluded**				
ICTI vs AC	Week 4	0.76	0.21	1.32
ICTI vs TAU	Week 4	0.85	0.20	1.49
ICTI vs AC	Week 12	0.53	-0.06	1.12
ICTI vs TAU	Week 12	0.86	0.19	1.52
ICTI vs AC	Week 24	0.38	-0.20	0.96
ICTI vs TAU	Week 24	1.09	0.42	1.76
**Outlier included**				
ICTI vs AC	Week 4	0.76	0.21	1.32
ICTI vs TAU	Week 4	0.27	-0.34	0.88
ICTI vs AC	Week 12	0.53	-0.06	1.12
ICTI vs TAU	Week 12	0.23	-0.40	0.86
ICTI vs AC	Week 24	0.38	-0.20	0.96
ICTI vs TAU	Week 24	0.38	-0.24	1.00

Note. Cohen's d estimated difference in the reduction of IMs from baseline between study arms (comparator arm minus ICTI) at Weeks 4, 12 and 24. On inspection, an outlier in the TAU arm was identified with high residual, leverage, and influence values and was excluded. (For direct comparisons without baseline, see Table S5D).

Abbreviations. ICTI = Imagery-Competing Task Intervention arm; AC = active control arm; TAU = treatment-as-usual; CI = confidence interval.

Table S5D. Post-hoc analysis. Benefits of ICTI on IMs estimated Cohen's d effect size

Comparison	Week	Cohen's d	Lower 95% CI	Upper 95% CI
**Outlier excluded**				
ICTI vs AC	Week 4	0.73	0.18	1.28
ICTI vs TAU	Week 4	0.78	0.14	1.42
ICTI vs AC	Week 12	0.67	0.08	1.26
ICTI vs TAU	Week 12	0.65	0.00	1.31
ICTI vs AC	Week 24	0.98	0.37	1.59
ICTI vs TAU	Week 24	1.16	0.48	1.83
**Outlier included**				
ICTI vs AC	Week 4	0.73	0.18	1.28
ICTI vs TAU	Week 4	0.76	0.13	1.39
ICTI vs AC	Week 12	0.67	0.08	1.26
ICTI vs TAU	Week 12	0.60	-0.04	1.24
ICTI vs AC	Week 24	0.98	0.37	1.59
ICTI vs TAU	Week 24	1.08	0.42	1.74

Note. Cohen's d estimated difference in IMs between study arms (comparator minus ICTI) at Weeks 4, 12 and 24. On inspection, an outlier in the TAU arm was identified with high residual, leverage, and influence values and was excluded. (For change from baseline comparisons, see Table S5C).

Abbreviations. ICTI = Imagery-Competing Task Intervention arm; AC = active control arm; TAU = treatment-as-usual; CI = confidence interval.

Figure S6. Secondary outcome: Intervention efficacy on IMs sustained over time. Posterior trace and density plots.

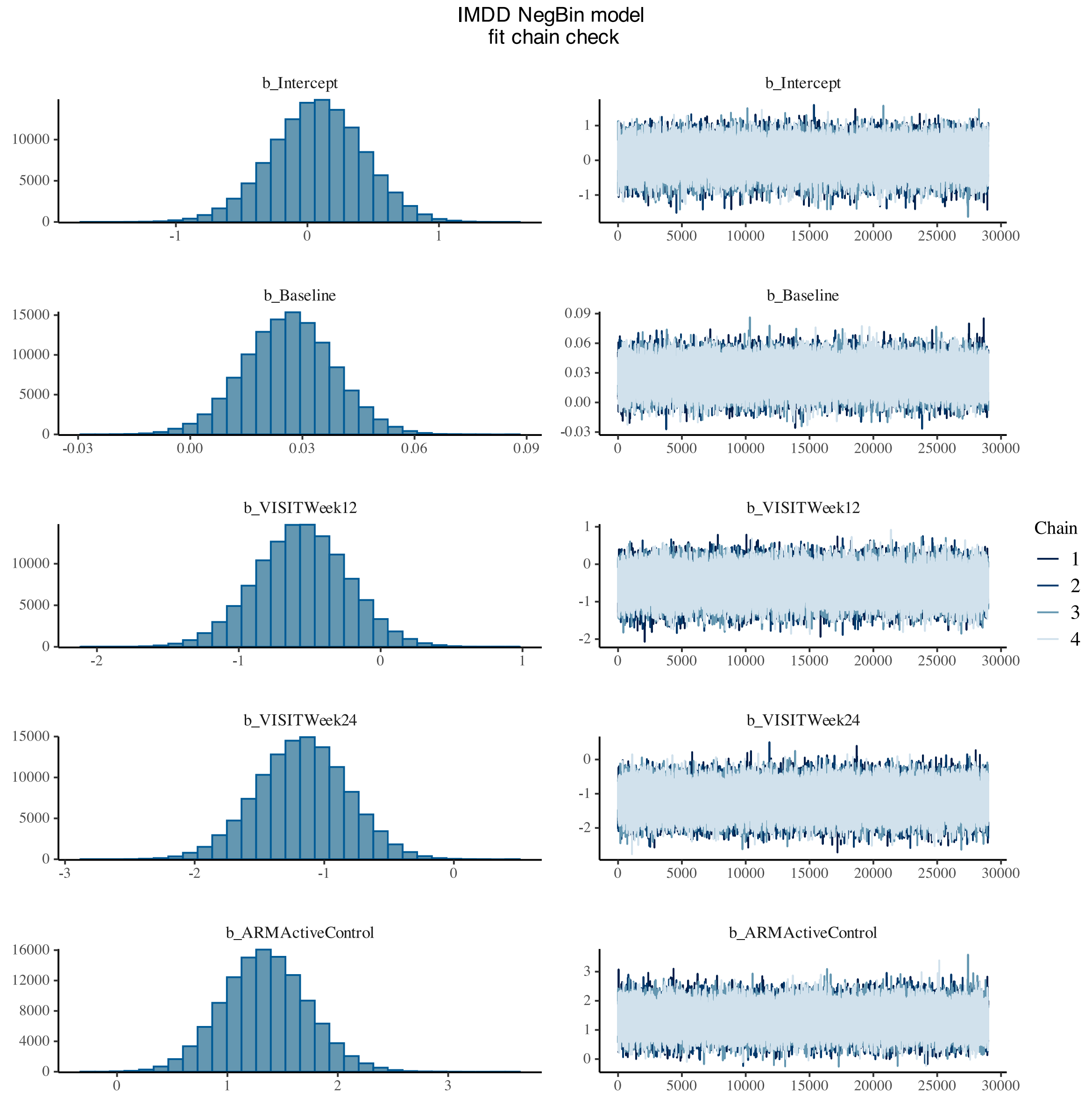

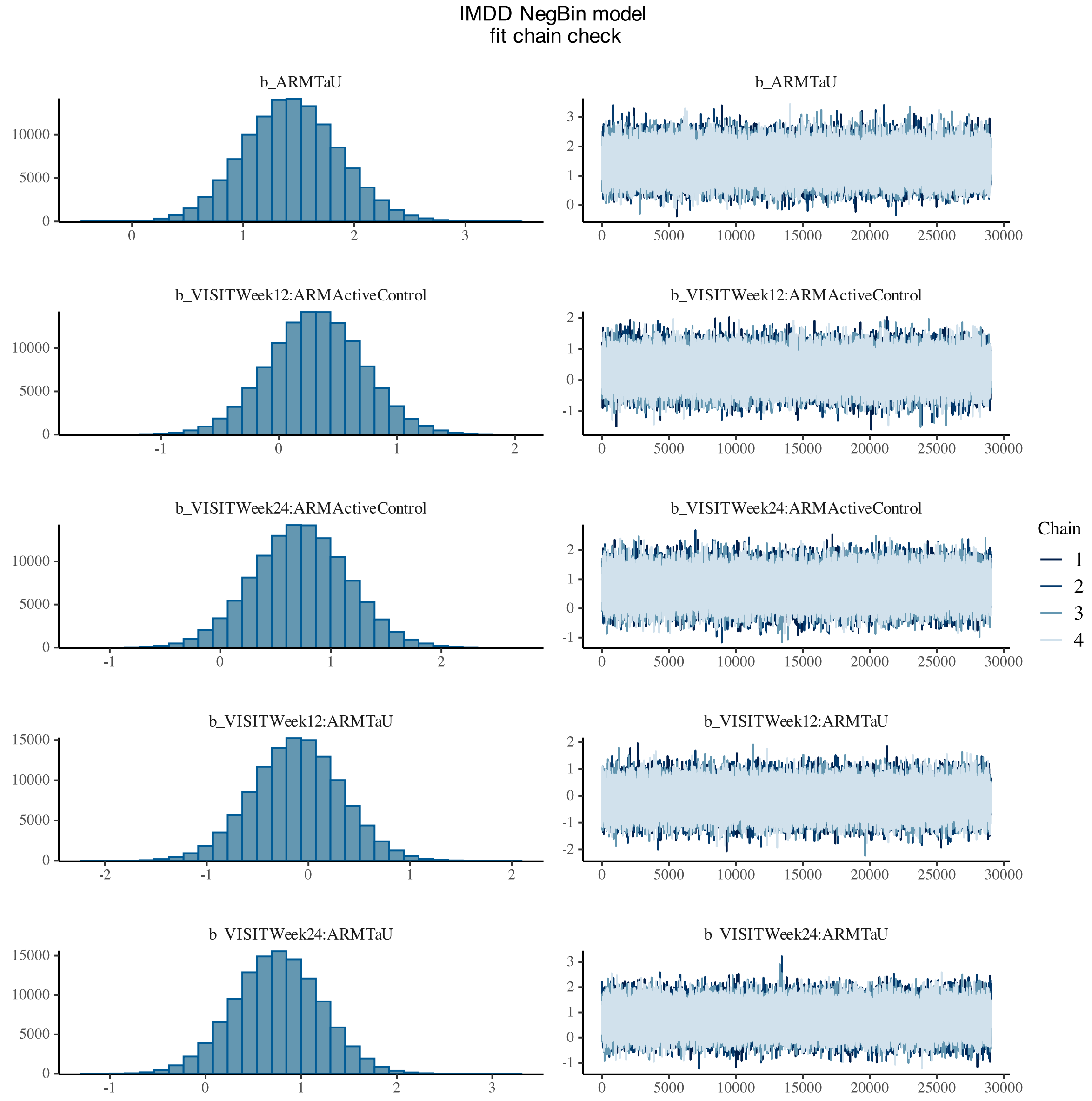
IMDD NegBin model
fit chain check
b_ARMTaU
b_VISITWeek12:ARMActiveControl
b_VISITWeek24:ARMActiveControl
b_VISITWeek12:ARMTaU
b_VISITWeek24:ARMTaU
Chain
1
2
3
4

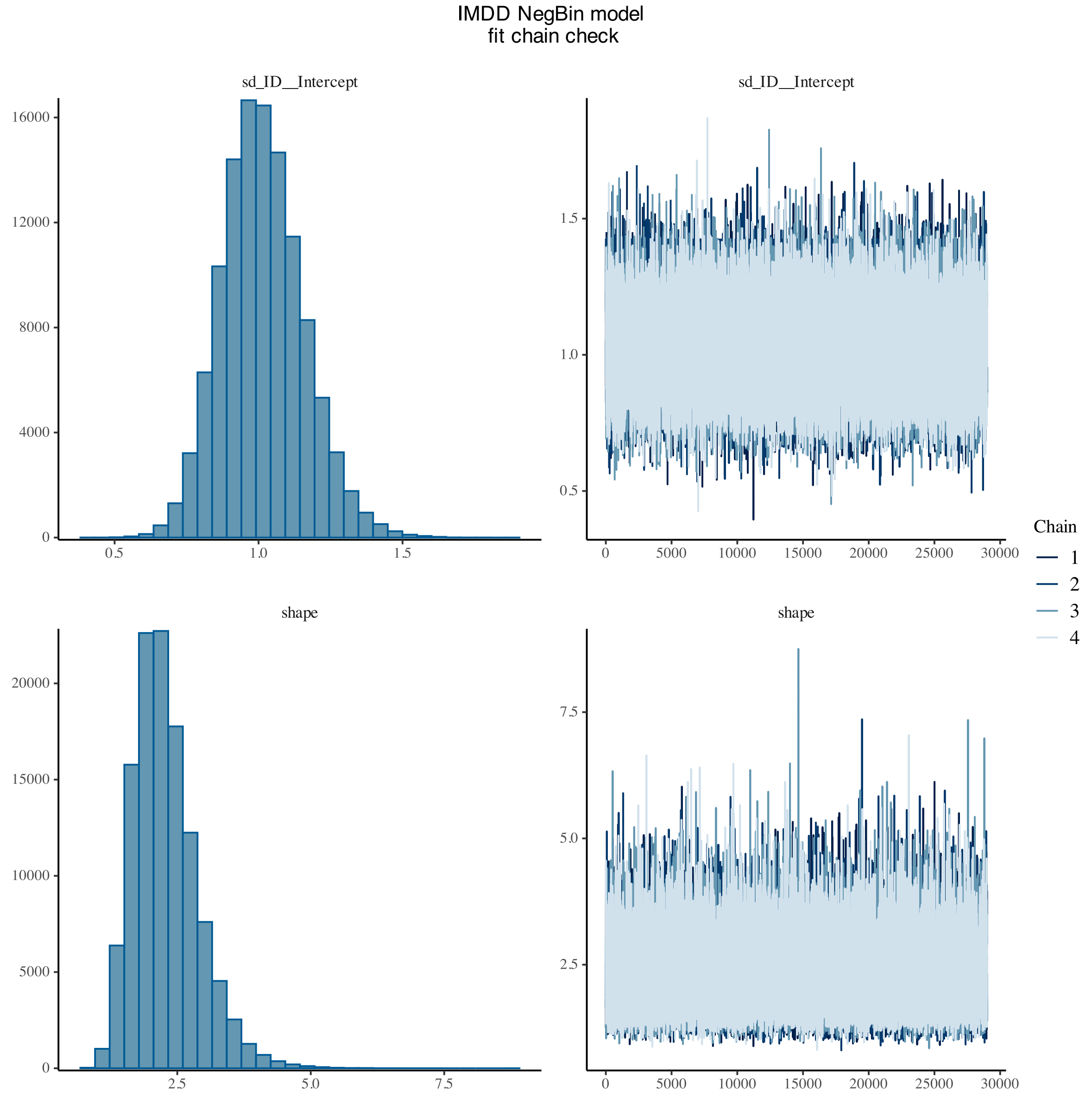
IMDD NegBin model
fit chain check
sd_ID__Intercept
16000
12000
8000
4000
0
0.5
1.0
1.5
sd_ID__Intercept
1.5
1.0
0.5
0
5000
10000
15000
20000
25000
30000
Chain
1
2
3
4
shape
20000
15000
10000
5000
0
2.5
5.0
7.5
shape
7.5
5.0
2.5
0
5000
10000
15000
20000
25000
30000

Figure S7. Secondary outcome: Intervention efficacy on IMs sustained over time. Posterior predictive checks. (A) a density overlay plot comparing the observed data (dark line) with simulated datasets drawn from the posterior predictive distribution (light lines). (B) the empirical cumulative distribution function (ECDF) overlay, comparing the ECDF of the observed data (dark line) to those from posterior predictive simulations (light lines)

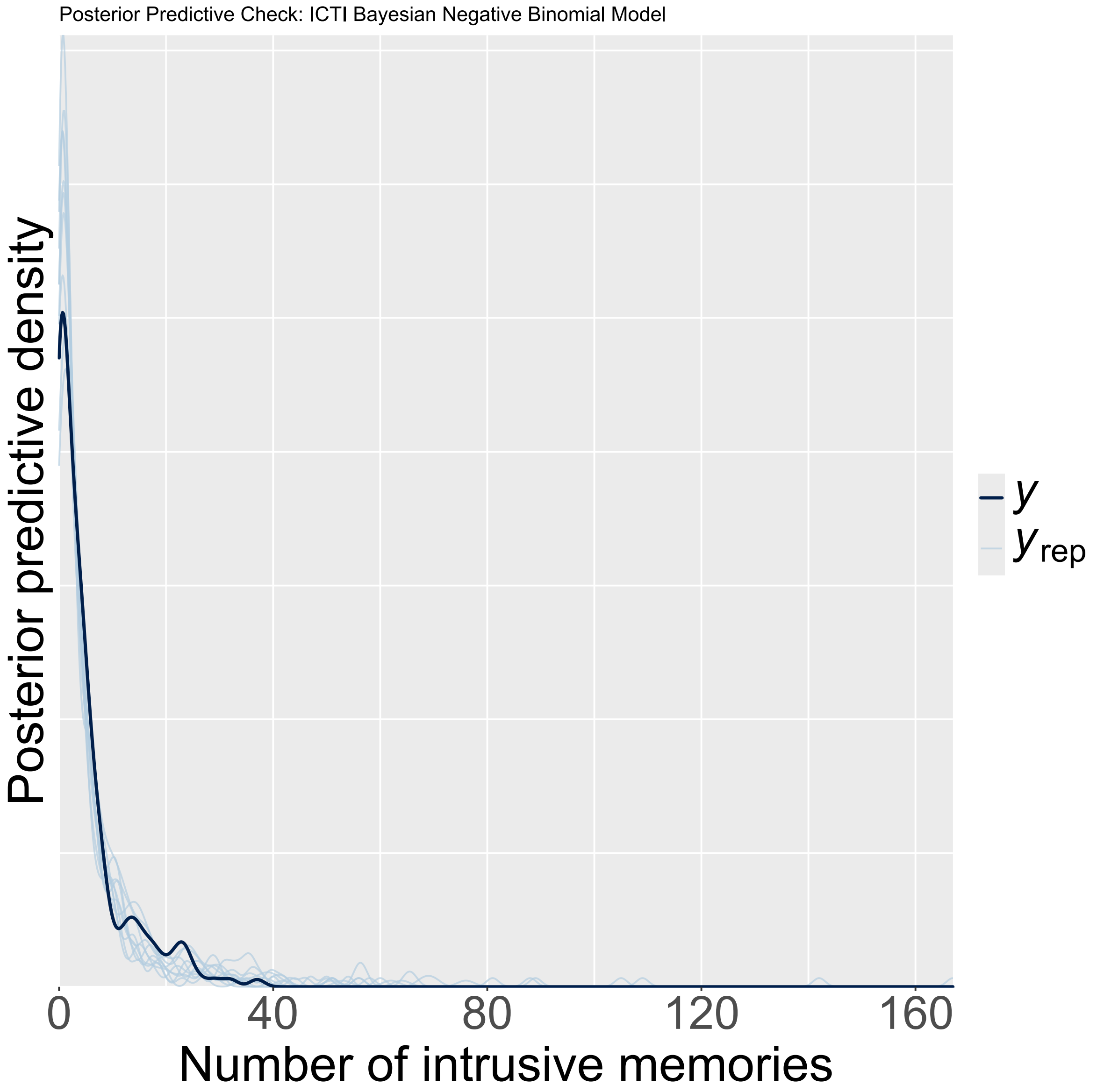

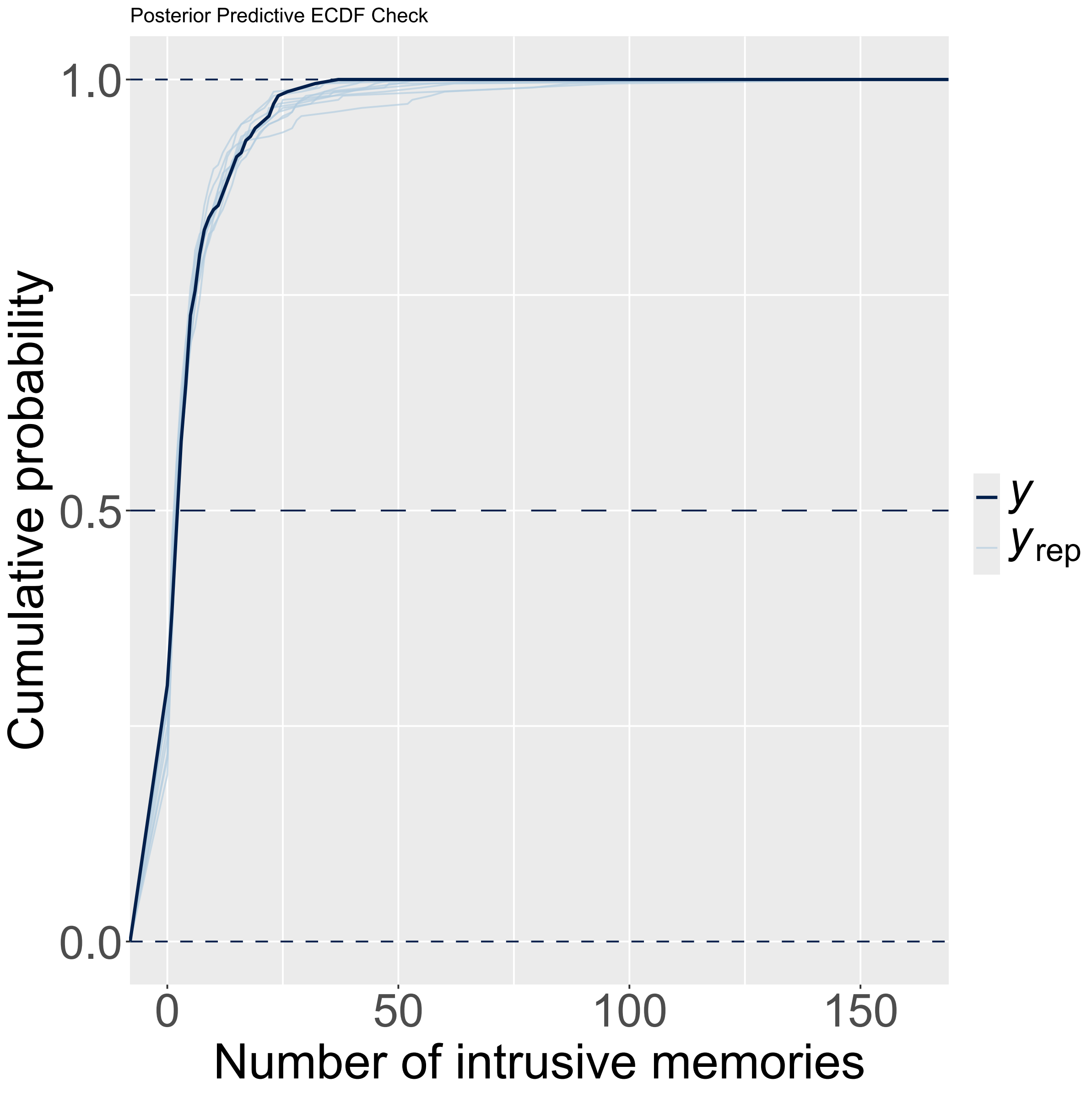
Posterior Predictive ECDF Check
1.0
0.5
0.0
Cumulative probability
0
50
100
150
Number of intrusive memories
y
y rep

Figure S8. Secondary outcomes: Intervention efficacy on IMs sustained over time, Posterior density plots with varying priors

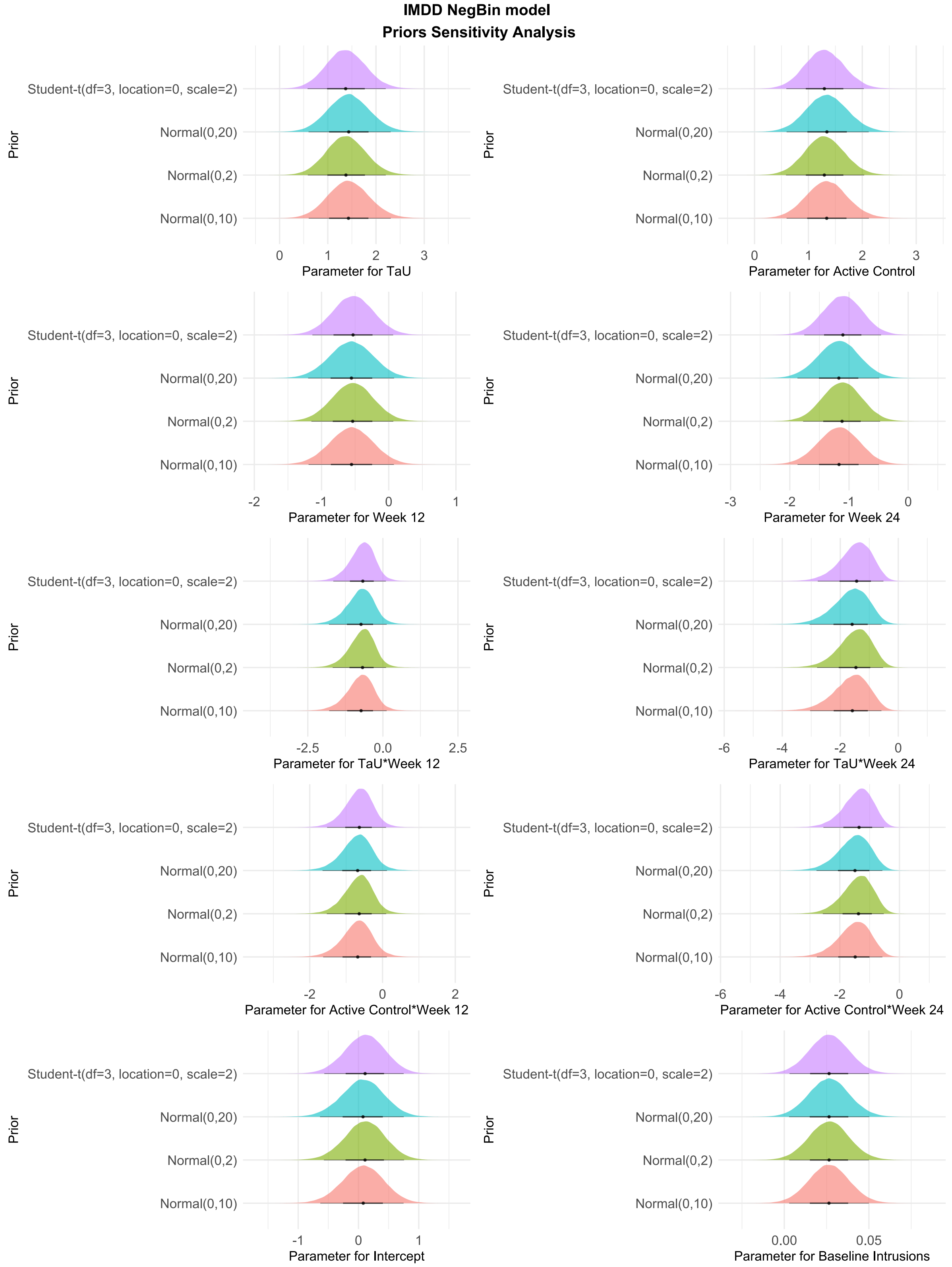

Table S6. Descriptive statistics for secondary outcomes

	Baseline				Week 4				Week 12				Week 24			
**Variable**	**All**	**TAU**	**AC**	**ICTI**	**All**	**TAU**	**AC**	**ICTI**	**All**	**TAU**	**AC**	**ICTI**	**All**	**TAU**	**AC**	**ICTI**
**N,**	99	20	39	40	72	18	29	25	64	19	24	21	68	18	24	26
****PTSD Symptoms****																
**PCL-5 Total Symptom Severity Score, all participants**																
N	99	20	39	40	69	17	27	25	64	19	24	21	67	17	24	26
Mean (SD)	32.99 (16.91)	34.95 (14.12)	32.64 (17.14)	32.35 (18.23)	23.14 (15.47)	30.94 (14.18)	26.85 (17.10)	13.84 (8.86)	18.08 (15.14)	23.79 (15.70)	19.88 (16.18)	10.86 (10.49)	14.43 (12.79)	21.29 (15.83)	16.08 (11.89)	8.42 (8.32)
Median (IQR)	32.00 (21.50 - 44.50)	29.50 (25.75 - 46.00)	34.00 (20.50 - 42.00)	32.50 (15.75 - 45.25)	24.00 (11.00 - 30.00)	30.00 (24.00 - 38.00)	27.00 (15.50 - 37.50)	12.00 (8.00 - 19.00)	13.00 (5.00 - 27.00)	20.00 (11.50 - 38.00)	15.00 (5.00 - 35.25)	6.00 (4.00 - 18.00)	12.00 (5.00 - 22.50)	16.00 (11.00 - 34.00)	15.50 (6.75 - 24.25)	6.50 (0.25 - 13.50)
Min - Max	1 - 75	10 - 62	1 - 75	3 - 67	1 - 66	8 - 65	1 - 66	1 - 33	0 - 54	3 - 54	0 - 51	0 - 32	0 - 51	3 - 51	0 - 45	0 - 27
**PCL-5 Total Symptom Severity Score, probable PTSD at baseline**																
N	48	8	20	20	31	7	14	10	26	7	10	9	25	6	9	10
Mean (SD)	47.23 (10.38)	50.00 (7.78)	46.20 (11.17)	47.15 (10.72)	32.19 (15.92)	42.29 (12.46)	36.93 (14.51)	18.50 (10.69)	25.96 (15.56)	33.71 (16.98)	27.80 (16.27)	17.89 (10.62)	18.80 (14.54)	28.67 (18.85)	21.11 (13.28)	10.80 (8.24)
Median (IQR)	45.00 (38.00 - 56.00)	50.50 (44.25 - 54.75)	42.00 (38.00 - 51.00)	46.50 (36.75 - 56.00)	29.00 (25.00 - 41.00)	39.00 (37.50 - 46.00)	30.00 (27.00 - 45.50)	19.50 (12.50 - 26.50)	26.50 (12.25 - 37.50)	38.00 (23.50 - 45.00)	31.50 (12.75 - 38.25)	18.00 (12.00 - 26.00)	16.00 (8.00 - 25.00)	31.50 (15.25 - 41.75)	24.00 (13.00 - 25.00)	10.50 (6.50 - 15.00)
Min - Max	33 - 75	39 - 62	34 - 75	33 - 67	1 - 66	25 - 65	15 - 66	1 - 33	1 - 54	7 - 54	5 - 51	1 - 32	0 - 51	3 - 51	0 - 45	0 - 27
**PCL-5 Total Symptom Severity Score, no PTSD at baseline**																
N	51	12	19	20	38	10	13	15	38	12	14	12	42	11	15	16
Mean (SD)	19.59 (9.01)	24.92 (5.58)	18.37 (8.31)	17.55 (10.32)	15.76 (10.48)	23.00 (9.17)	16.00 (12.62)	10.73 (5.95)	12.68 (12.38)	18.00 (12.12)	14.21 (14.03)	5.58 (6.87)	11.83 (11.01)	17.27 (13.15)	13.07 (10.27)	6.94 (8.28)
Median (IQR)	22.00 (11.50 - 27.00)	26.50 (24.25 - 28.25)	20.00 (15.00 - 24.00)	15.50 (8.75 - 27.25)	13.50 (8.00 - 23.50)	25.00 (15.25 - 29.50)	16.00 (5.00 - 22.00)	9.00 (7.50 - 13.00)	7.50 (4.25 - 16.50)	13.00 (10.75 - 24.00)	10.00 (4.25 - 21.75)	5.00 (1.75 - 6.00)	10.00 (3.25 - 17.75)	12.00 (9.00 - 19.50)	11.00 (5.00 - 22.00)	4.00 (0.00 - 12.50)
Min - Max	1 - 32	10 - 30	1 - 29	3 - 32	1 - 38	8 - 36	1 - 38	1 - 25	0 - 46	3 - 42	0 - 46	0 - 26	0 - 48	5 - 48	0 - 31	0 - 24
**PCL-5 Reexperiencing subscore**																
N	99	20	39	40	69	17	27	25	64	19	24	21	67	17	24	26
Mean (SD)	8.72 (4.58)	9.05 (4.49)	8.18 (4.55)	9.08 (4.73)	5.61 (4.23)	7.00 (4.81)	6.74 (4.31)	3.44 (2.74)	4.31 (4.28)	5.26 (4.43)	5.13 (4.56)	2.52 (3.33)	3.28 (3.41)	4.18 (4.19)	4.13 (3.54)	1.92 (2.19)
Median (IQR)	8.00 (5.00 - 12.00)	7.00 (6.00 - 12.50)	8.00 (5.00 - 11.00)	9.50 (5.75 - 13.00)	4.00 (3.00 - 8.00)	7.00 (3.00 - 8.00)	7.00 (4.00 - 9.00)	3.00 (1.00 - 5.00)	3.00 (1.00 - 7.25)	5.00 (1.00 - 8.50)	4.00 (1.75 - 9.00)	1.00 (0.00 - 4.00)	2.00 (1.00 - 5.00)	3.00 (1.00 - 5.00)	3.00 (1.75 - 6.00)	1.50 (0.00 - 3.00)

	Baseline				Week 4				Week 12				Week 24			
**Variable**	**All**	**TAU**	**AC**	**ICTI**	**All**	**TAU**	**AC**	**ICTI**	**All**	**TAU**	**AC**	**ICTI**	**All**	**TAU**	**AC**	**ICTI**
Min - Max	0 - 20	3 - 18	1 - 20	0 - 18	0 - 17	0 - 17	0 - 17	0 - 9	0 - 15	0 - 15	0 - 15	0 - 11	0 - 15	0 - 14	0 - 15	0 - 7
**PCL-5 Avoidance subscore**																
N	99	20	39	40	69	17	27	25	64	19	24	21	67	17	24	26
Mean (SD)	4.37 (2.30)	4.85 (1.73)	4.18 (2.22)	4.33 (2.62)	2.93 (2.26)	3.59 (2.35)	3.33 (2.13)	2.04 (2.13)	2.52 (2.42)	3.32 (2.26)	3.08 (2.64)	1.14 (1.68)	2.15 (2.23)	2.94 (2.54)	2.50 (2.38)	1.31 (1.59)
Median (IQR)	4.00 (3.00 - 6.00)	5.00 (4.00 - 6.00)	4.00 (3.00 - 6.00)	5.00 (2.00 - 6.25)	3.00 (1.00 - 4.00)	3.00 (1.00 - 5.00)	3.00 (2.00 - 5.00)	2.00 (0.00 - 3.00)	2.00 (0.00 - 5.00)	3.00 (1.50 - 5.00)	3.00 (0.00 - 6.00)	1.00 (0.00 - 1.00)	2.00 (0.00 - 3.00)	2.00 (1.00 - 5.00)	2.00 (1.00 - 3.25)	0.50 (0.00 - 2.00)
Min - Max	0 - 8	2 - 8	0 - 8	0 - 8	0 - 8	1 - 8	0 - 7	0 - 8	0 - 7	0 - 7	0 - 7	0 - 6	0 - 8	0 - 8	0 - 8	0 - 5
**PCL-5 Negative alterations subscore**																
N	99	20	39	40	69	17	27	25	64	19	24	21	67	17	24	26
Mean (SD)	11.19 (6.84)	12.70 (6.38)	10.92 (6.57)	10.70 (7.37)	8.12 (6.31)	11.82 (6.46)	9.19 (6.87)	4.44 (2.96)	6.17 (5.84)	8.26 (6.41)	6.83 (6.40)	3.52 (3.34)	4.70 (4.81)	7.41 (6.22)	5.13 (4.52)	2.54 (2.76)
Median (IQR)	12.00 (5.00 - 17.00)	12.50 (9.00 - 16.50)	10.00 (6.50 - 16.50)	11.00 (4.75 - 17.00)	7.00 (3.00 - 11.00)	12.00 (7.00 - 15.00)	8.00 (4.00 - 13.00)	4.00 (2.00 - 6.00)	4.00 (1.00 - 10.00)	7.00 (4.00 - 10.50)	5.50 (1.00 - 12.25)	3.00 (1.00 - 4.00)	3.00 (0.50 - 7.00)	6.00 (3.00 - 10.00)	5.00 (1.00 - 8.25)	1.50 (0.00 - 5.00)
Min - Max	0 - 25	1 - 24	0 - 23	0 - 25	0 - 24	2 - 24	0 - 23	0 - 10	0 - 23	1 - 23	0 - 21	0 - 11	0 - 21	0 - 21	0 - 16	0 - 8
**PCL-5 Hyper arousal subscore**																
N	99	20	39	40	69	17	27	25	64	19	24	21	67	17	24	26
Mean (SD)	8.71 (5.48)	8.35 (4.56)	9.36 (5.60)	8.25 (5.84)	6.49 (4.85)	8.53 (4.50)	7.59 (5.46)	3.92 (3.12)	5.08 (4.62)	6.95 (5.42)	4.83 (4.04)	3.67 (4.08)	4.30 (4.02)	6.76 (5.17)	4.33 (3.31)	2.65 (2.92)
Median (IQR)	8.00 (4.00 - 13.00)	6.50 (5.00 - 11.25)	9.00 (6.00 - 12.00)	7.00 (3.00 - 13.25)	7.00 (2.00 - 9.00)	8.00 (7.00 - 11.00)	8.00 (2.00 - 10.50)	3.00 (1.00 - 6.00)	3.00 (2.00 - 8.00)	6.00 (3.00 - 9.50)	3.50 (2.00 - 7.25)	2.00 (1.00 - 4.00)	3.00 (1.00 - 6.50)	5.00 (3.00 - 11.00)	4.00 (1.75 - 7.00)	2.00 (0.00 - 5.00)
Min - Max	0 - 24	2 - 19	0 - 24	0 - 19	0 - 20	0 - 18	0 - 20	0 - 10	0 - 19	0 - 19	0 - 14	0 - 14	0 - 14	0 - 14	0 - 12	0 - 11
****Other Clinical Outcomes****																
**Insomnia Symptoms, SCI-2 Total Score**																
N	99	20	39	40	68	17	27	24	64	19	24	21	66	17	24	25
Mean (SD)	3.04 (2.44)	2.60 (2.30)	3.28 (2.60)	3.03 (2.38)	3.66 (2.69)	2.82 (2.81)	3.93 (2.93)	3.96 (2.27)	4.55 (2.53)	3.74 (2.58)	4.83 (2.46)	4.95 (2.52)	4.58 (2.71)	3.71 (2.87)	4.42 (2.72)	5.32 (2.48)
Median (IQR)	2.00 (1.00 - 5.00)	2.00 (0.75 - 5.00)	3.00 (1.00 - 5.00)	2.00 (1.75 - 5.00)	3.00 (1.00 - 6.00)	3.00 (0.00 - 4.00)	4.00 (1.00 - 7.00)	3.50 (2.00 - 6.00)	5.00 (2.00 - 7.00)	4.00 (1.50 - 6.00)	6.00 (2.00 - 7.00)	6.00 (4.00 - 7.00)	5.00 (2.00 - 7.00)	3.00 (1.00 - 6.00)	5.00 (2.00 - 6.25)	6.00 (4.00 - 7.00)
Min - Max	0 - 8	0 - 6	0 - 8	0 - 8	0 - 8	0 - 8	0 - 8	0 - 8	0 - 8	0 - 8	1 - 8	0 - 8	0 - 8	0 - 8	0 - 8	0 - 8
**Anxiety Symptoms, GAD-2 Total Score**																

Variable	Baseline				Week 4				Week 12				Week 24			
	All	TAU	AC	ICTI	All	TAU	AC	ICTI	All	TAU	AC	ICTI	All	TAU	AC	ICTI
N	99	20	39	40	68	17	27	24	64	19	24	21	66	17	24	25
Mean (SD)	2.72 (1.90)	2.90 (2.17)	2.44 (1.76)	2.90 (1.91)	2.13 (1.61)	2.53 (1.70)	2.33 (1.75)	1.63 (1.28)	1.97 (1.77)	2.68 (2.03)	1.96 (1.78)	1.33 (1.28)	1.52 (1.42)	2.06 (1.75)	1.50 (1.38)	1.16 (1.11)
Median (IQR)	2.00 (1.50 - 4.00)	2.00 (1.00 - 5.00)	2.00 (2.00 - 3.00)	2.50 (1.75 - 4.00)	2.00 (1.00 - 3.00)	2.00 (1.00 - 3.00)	2.00 (1.00 - 3.50)	2.00 (1.00 - 2.00)	2.00 (0.75 - 2.00)	2.00 (1.50 - 4.00)	2.00 (0.75 - 2.00)	1.00 (0.00 - 2.00)	1.00 (0.00 - 2.00)	2.00 (1.00 - 3.00)	1.50 (0.75 - 2.00)	1.00 (0.00 - 2.00)
Min - Max	0 - 6	0 - 6	0 - 6	0 - 6	0 - 6	0 - 6	0 - 6	0 - 5	0 - 6	0 - 6	0 - 6	0 - 5	0 - 6	0 - 6	0 - 6	0 - 4
**Depression Symptoms, PHQ-2 Total Score**																
N	99	20	39	40	68	17	27	24	64	19	24	21	66	17	24	25
Mean (SD)	2.13 (1.59)	2.25 (1.97)	2.10 (1.59)	2.10 (1.43)	1.49 (1.55)	2.18 (2.21)	1.63 (1.36)	0.83 (0.82)	1.38 (1.52)	1.79 (1.65)	1.38 (1.74)	1.00 (1.00)	1.05 (1.26)	1.47 (1.50)	1.04 (1.27)	0.76 (1.01)
Median (IQR)	2.00 (1.00 - 3.00)	2.00 (0.75 - 2.50)	2.00 (1.00 - 3.00)	2.00 (1.00 - 3.00)	1.00 (0.00 - 2.00)	2.00 (0.00 - 4.00)	2.00 (0.00 - 2.00)	1.00 (0.00 - 1.25)	1.00 (0.00 - 2.00)	2.00 (0.00 - 3.00)	1.00 (0.00 - 2.00)	1.00 (0.00 - 2.00)	1.00 (0.00 - 2.00)	1.00 (0.00 - 2.00)	0.50 (0.00 - 2.00)	0.00 (0.00 - 1.00)
Min - Max	0 - 6	0 - 6	0 - 6	0 - 6	0 - 6	0 - 6	0 - 5	0 - 2	0 - 6	0 - 5	0 - 6	0 - 3	0 - 5	0 - 5	0 - 5	0 - 4
****Functional outcomes, quality of life and occupational measures****																
**General Functioning, WHODAS 12-item Total Score**																
N	99	20	39	40	67	17	26	24	64	19	24	21	65	16	24	25
Mean (SD)	21.09 (15.44)	15.63 (10.70)	21.37 (14.88)	23.55 (17.48)	16.64 (13.75)	16.42 (10.62)	18.27 (15.06)	15.02 (14.55)	15.60 (14.98)	18.09 (16.74)	13.29 (11.42)	15.97 (17.09)	14.97 (15.41)	18.36 (15.63)	14.50 (16.57)	13.25 (14.37)
Median (IQR)	16.67 (10.42 - 29.17)	16.67 (4.17 - 23.44)	18.75 (10.42 - 30.21)	17.71 (10.42 - 32.29)	12.50 (6.25 - 25.00)	16.67 (6.25 - 25.00)	15.63 (6.25 - 25.00)	9.38 (4.17 - 20.31)	10.42 (4.17 - 23.44)	14.59 (4.17 - 26.05)	10.42 (5.73 - 19.27)	8.34 (4.17 - 22.92)	10.42 (2.09 - 25.00)	17.71 (3.65 - 26.04)	7.30 (1.57 - 21.88)	8.34 (0.00 - 22.92)
Min - Max	0 - 71	2 - 38	0 - 71	0 - 60	0 - 56	0 - 35	0 - 56	0 - 46	0 - 56	0 - 56	0 - 40	0 - 50	0 - 63	0 - 54	0 - 63	0 - 42
**General Functioning, EQ-5D-5L**																
**Mobility, n (%)**																
I have no problems in walking about	74 (74.7%)	19 (95.0%)	24 (61.5%)	31 (77.5%)	54 (80.6%)	15 (88.2%)	21 (80.8%)	18 (75.0%)	54 (84.4%)	16 (84.2%)	21 (87.5%)	17 (81.0%)	50 (76.9%)	14 (87.5%)	19 (79.2%)	17 (68.0%)
I have slight problems in walking about	18 (18.2%)	1 (5.0%)	10 (25.6%)	7 (17.5%)	9 (13.4%)	2 (11.8%)	4 (15.4%)	3 (12.5%)	6 (9.4%)	2 (10.5%)	3 (12.5%)	1 (4.8%)	12 (18.5%)	2 (12.5%)	5 (20.8%)	5 (20.0%)
I have moderate problems in walking about	5 (5.1%)	0 (0.0%)	4 (10.3%)	1 (2.5%)	4 (6.0%)	0 (0.0%)	1 (3.8%)	3 (12.5%)	3 (4.7%)	1 (5.3%)	0 (0.0%)	2 (9.5%)	3 (4.6%)	0 (0.0%)	0 (0.0%)	3 (12.0%)
I have severe problems in walking about	2 (2.0%)	0 (0.0%)	1 (2.6%)	1 (2.5%)	0 (0.0%)	0 (0.0%)	0 (0.0%)	0 (0.0%)	1 (1.6%)	0 (0.0%)	0 (0.0%)	1 (4.8%)	0 (0.0%)	0 (0.0%)	0 (0.0%)	0 (0.0%)
I am unable to walk about	0 (0.0%)	0 (0.0%)	0 (0.0%)	0 (0.0%)	0 (0.0%)	0 (0.0%)	0 (0.0%)	0 (0.0%)	0 (0.0%)	0 (0.0%)	0 (0.0%)	0 (0.0%)	0 (0.0%)	0 (0.0%)	0 (0.0%)	0 (0.0%)

	Baseline				Week 4				Week 12				Week 24			
Variable	All	TAU	AC	ICTI	All	TAU	AC	ICTI	All	TAU	AC	ICTI	All	TAU	AC	ICTI
**Self-care, n (%)**																
I have no problems washing or dressing myself	85 (85.9%)	18 (90.0%)	34 (87.2%)	33 (82.5%)	56 (83.6%)	15 (88.2%)	21 (80.8%)	20 (83.3%)	58 (90.6%)	17 (89.5%)	24 (100.0%)	17 (81.0%)	57 (87.7%)	14 (87.5%)	22 (91.7%)	21 (84.0%)
I have slight problems washing or dressing myself	8 (8.1%)	1 (5.0%)	4 (10.3%)	3 (7.5%)	9 (13.4%)	1 (5.9%)	5 (19.2%)	3 (12.5%)	4 (6.3%)	1 (5.3%)	0 (0.0%)	3 (14.3%)	7 (10.8%)	1 (6.3%)	2 (8.3%)	4 (16.0%)
I have moderate problems washing or dressing myself	4 (4.0%)	1 (5.0%)	0 (0.0%)	3 (7.5%)	2 (3.0%)	1 (5.9%)	0 (0.0%)	1 (4.2%)	0 (0.0%)	0 (0.0%)	0 (0.0%)	0 (0.0%)	1 (1.5%)	1 (6.3%)	0 (0.0%)	0 (0.0%)
I have severe problems washing or dressing myself	2 (2.0%)	0 (0.0%)	1 (2.6%)	1 (2.5%)	0 (0.0%)	0 (0.0%)	0 (0.0%)	0 (0.0%)	2 (3.1%)	1 (5.3%)	0 (0.0%)	1 (4.8%)	0 (0.0%)	0 (0.0%)	0 (0.0%)	0 (0.0%)
I am unable to wash or dress myself	0 (0.0%)	0 (0.0%)	0 (0.0%)	0 (0.0%)	0 (0.0%)	0 (0.0%)	0 (0.0%)	0 (0.0%)	0 (0.0%)	0 (0.0%)	0 (0.0%)	0 (0.0%)	0 (0.0%)	0 (0.0%)	0 (0.0%)	0 (0.0%)
**Usual activities, n (%)**																
I have no problems doing my usual activities	53 (53.5%)	13 (65.0%)	19 (48.7%)	21 (52.5%)	45 (67.2%)	10 (58.8%)	19 (73.1%)	16 (66.7%)	47 (73.4%)	15 (78.9%)	19 (79.2%)	13 (61.9%)	42 (64.6%)	10 (62.5%)	16 (66.7%)	16 (64.0%)
I have slight problems doing my usual activities	30 (30.3%)	7 (35.0%)	12 (30.8%)	11 (27.5%)	12 (17.9%)	7 (41.2%)	3 (11.5%)	2 (8.3%)	12 (18.8%)	3 (15.8%)	4 (16.7%)	5 (23.8%)	15 (23.1%)	4 (25.0%)	6 (25.0%)	5 (20.0%)
I have moderate problems doing my usual activities	12 (12.1%)	0 (0.0%)	7 (17.9%)	5 (12.5%)	9 (13.4%)	0 (0.0%)	3 (11.5%)	6 (25.0%)	5 (7.8%)	1 (5.3%)	1 (4.2%)	3 (14.3%)	7 (10.8%)	2 (12.5%)	1 (4.2%)	4 (16.0%)
I have severe problems doing my usual activities	4 (4.0%)	0 (0.0%)	1 (2.6%)	3 (7.5%)	0 (0.0%)	0 (0.0%)	0 (0.0%)	0 (0.0%)	0 (0.0%)	0 (0.0%)	0 (0.0%)	0 (0.0%)	1 (1.5%)	0 (0.0%)	1 (4.2%)	0 (0.0%)
I am unable to do my usual activities	0 (0.0%)	0 (0.0%)	0 (0.0%)	0 (0.0%)	1 (1.5%)	0 (0.0%)	1 (3.8%)	0 (0.0%)	0 (0.0%)	0 (0.0%)	0 (0.0%)	0 (0.0%)	0 (0.0%)	0 (0.0%)	0 (0.0%)	0 (0.0%)
**Pain/discomfort, n (%)**																
I have no pain or discomfort	37 (37.4%)	9 (45.0%)	16 (41.0%)	12 (30.0%)	27 (40.3%)	7 (41.2%)	9 (34.6%)	11 (45.8%)	26 (40.6%)	9 (47.4%)	8 (33.3%)	9 (42.9%)	30 (46.2%)	9 (56.3%)	10 (41.7%)	11 (44.0%)
I have slight pain or discomfort	36 (36.4%)	8 (40.0%)	11 (28.2%)	17 (42.5%)	24 (35.8%)	7 (41.2%)	12 (46.2%)	5 (20.8%)	24 (37.5%)	8 (42.1%)	11 (45.8%)	5 (23.8%)	18 (27.7%)	4 (25.0%)	8 (33.3%)	6 (24.0%)
I have moderate pain or discomfort	17 (17.2%)	3 (15.0%)	8 (20.5%)	6 (15.0%)	8 (11.9%)	3 (17.6%)	3 (11.5%)	2 (8.3%)	11 (17.2%)	2 (10.5%)	4 (16.7%)	5 (23.8%)	13 (20.0%)	3 (18.8%)	5 (20.8%)	5 (20.0%)
I have severe pain or discomfort	4 (4.0%)	0 (0.0%)	1 (2.6%)	3 (7.5%)	7 (10.4%)	0 (0.0%)	1 (3.8%)	6 (25.0%)	3 (4.7%)	0 (0.0%)	1 (4.2%)	2 (9.5%)	3 (4.6%)	0 (0.0%)	1 (4.2%)	2 (8.0%)
I have extreme pain or discomfort	5 (5.1%)	0 (0.0%)	3 (7.7%)	2 (5.0%)	1 (1.5%)	0 (0.0%)	1 (3.8%)	0 (0.0%)	0 (0.0%)	0 (0.0%)	0 (0.0%)	0 (0.0%)	1 (1.5%)	0 (0.0%)	0 (0.0%)	1 (4.0%)
**Anxiety/depression, n (%)**																
I am not anxious or depressed	16 (16.2%)	3 (15.0%)	7 (17.9%)	6 (15.0%)	18 (26.9%)	2 (11.8%)	8 (30.8%)	8 (33.3%)	17 (26.6%)	4 (21.1%)	7 (29.2%)	6 (28.6%)	22 (33.8%)	3 (18.8%)	10 (41.7%)	9 (36.0%)
I am slightly anxious or depressed	40 (40.4%)	8 (40.0%)	17 (43.6%)	15 (37.5%)	28 (41.8%)	6 (35.3%)	10 (38.5%)	12 (50.0%)	30 (46.9%)	8 (42.1%)	11 (45.8%)	11 (52.4%)	28 (43.1%)	7 (43.8%)	9 (37.5%)	12 (48.0%)

	Baseline				Week 4				Week 12				Week 24			
**Variable**	**All**	**TAU**	**AC**	**ICTI**	**All**	**TAU**	**AC**	**ICTI**	**All**	**TAU**	**AC**	**ICTI**	**All**	**TAU**	**AC**	**ICTI**
I am moderately anxious or depressed	30 (30.3%)	5 (25.0%)	11 (28.2%)	14 (35.0%)	17 (25.4%)	8 (47.1%)	5 (19.2%)	4 (16.7%)	16 (25.0%)	6 (31.6%)	6 (25.0%)	4 (19.0%)	11 (16.9%)	4 (25.0%)	4 (16.7%)	3 (12.0%)
I am severely anxious or depressed	11 (11.1%)	3 (15.0%)	4 (10.3%)	4 (10.0%)	4 (6.0%)	1 (5.9%)	3 (11.5%)	0 (0.0%)	1 (1.6%)	1 (5.3%)	0 (0.0%)	0 (0.0%)	4 (6.2%)	2 (12.5%)	1 (4.2%)	1 (4.0%)
I am extremely anxious or depressed	2 (2.0%)	1 (5.0%)	0 (0.0%)	1 (2.5%)	0 (0.0%)	0 (0.0%)	0 (0.0%)	0 (0.0%)	0 (0.0%)	0 (0.0%)	0 (0.0%)	0 (0.0%)	0 (0.0%)	0 (0.0%)	0 (0.0%)	0 (0.0%)
**Overall health (VAS) score, n (%)**																
N	99	20	39	40	66	16	26	24	64	19	24	21	64	16	23	25
Mean (SD)	69.41 (16.72)	70.80 (14.11)	71.79 (16.81)	66.40 (17.72)	72.27 (18.35)	74.53 (16.42)	70.08 (20.10)	73.04 (18.18)	74.45 (17.98)	75.21 (17.49)	75.17 (19.76)	72.95 (17.05)	74.00 (18.98)	71.63 (17.63)	75.75 (20.69)	73.84 (18.67)
Median (IQR)	70.00 (60.00 - 81.00)	70.00 (65.00 - 80.00)	71.00 (61.00 - 86.50)	70.00 (51.75 - 80.25)	76.00 (61.50 - 85.00)	75.00 (63.00 - 85.00)	72.50 (60.75 - 83.50)	79.00 (61.75 - 86.00)	79.50 (63.50 - 90.00)	80.00 (63.50 - 90.00)	77.50 (69.00 - 90.00)	80.00 (61.00 - 85.00)	79.00 (60.00 - 90.00)	73.00 (60.00 - 82.50)	80.50 (64.25 - 90.50)	80.00 (60.00 - 87.00)
Min - Max	30 - 97	41 - 97	39 - 96	30 - 91	20 - 100	40 - 100	20 - 98	39 - 95	28 - 99	40 - 97	28 - 99	36 - 95	10 - 100	40 - 98	10 - 100	30 - 95
**Intention to Leave the Job, ITL , Total Score**																
N	99	20	39	40	67	17	26	24	64	19	24	21	66	17	24	25
Mean (SD)	9.76 (3.88)	10.55 (3.83)	9.54 (3.97)	9.58 (3.87)	9.58 (4.58)	9.71 (4.51)	8.58 (4.99)	10.58 (4.11)	10.63 (4.38)	11.32 (3.99)	10.04 (4.95)	10.67 (4.13)	10.12 (4.45)	10.29 (4.73)	9.63 (4.94)	10.48 (3.87)
Median (IQR)	10.00 (6.50 - 13.00)	10.00 (7.00 - 14.25)	10.00 (7.00 - 12.50)	10.00 (6.00 - 13.00)	11.00 (5.00 - 14.00)	11.00 (7.00 - 14.00)	7.50 (3.00 - 14.00)	12.50 (8.50 - 14.00)	12.00 (7.00 - 14.25)	13.00 (9.50 - 15.00)	12.00 (4.75 - 14.00)	12.00 (7.00 - 14.00)	11.00 (6.25 - 14.75)	12.00 (7.00 - 15.00)	10.00 (5.25 - 15.00)	11.00 (7.00 - 14.00)
Min - Max	3 - 15	3 - 15	3 - 15	3 - 15	3 - 15	3 - 15	3 - 15	3 - 15	3 - 15	3 - 15	3 - 15	3 - 15	3 - 15	3 - 15	3 - 15	3 - 15
**Work Engagement, SWEBO Subscale**																
N	99	20	39	40	67	17	26	24	64	19	24	21	66	17	24	25
Mean (SD)	2.08 (0.57)	2.22 (0.63)	2.09 (0.62)	2.00 (0.47)	2.32 (0.55)	2.16 (0.46)	2.30 (0.62)	2.45 (0.50)	2.44 (0.59)	2.41 (0.62)	2.54 (0.66)	2.35 (0.47)	2.40 (0.67)	2.24 (0.76)	2.57 (0.83)	2.34 (0.37)
Median (IQR)	2.00 (1.70 - 2.40)	2.20 (1.60 - 2.65)	2.00 (1.70 - 2.55)	1.95 (1.70 - 2.30)	2.30 (1.90 - 2.65)	2.10 (1.80 - 2.60)	2.11 (1.90 - 2.68)	2.50 (2.28 - 2.90)	2.50 (2.00 - 2.80)	2.40 (1.95 - 2.75)	2.60 (2.08 - 3.00)	2.40 (2.00 - 2.60)	2.40 (2.00 - 2.90)	2.10 (1.70 - 3.00)	2.75 (2.00 - 3.13)	2.40 (2.10 - 2.60)
Min - Max	1 - 4	1 - 3	1 - 4	1 - 3	1 - 4	1 - 3	1 - 4	1 - 3	1 - 4	1 - 4	1 - 4	2 - 3	1 - 4	1 - 3	1 - 4	2 - 3
**Work Burnout, SWEBO Subscale**																
N	99	20	39	40	67	17	26	24	64	19	24	21	66	17	24	25
Mean (SD)	2.22 (0.63)	2.21 (0.64)	2.20 (0.68)	2.25 (0.58)	1.96 (0.55)	2.21 (0.55)	2.05 (0.63)	1.69 (0.33)	1.93 (0.60)	1.97 (0.60)	1.89 (0.71)	1.94 (0.45)	1.98 (0.65)	2.23 (0.70)	1.93 (0.79)	1.87 (0.41)

	Baseline				Week 4				Week 12				Week 24			
**Variable**	**All**	**TAU**	**AC**	**ICTI**	**All**	**TAU**	**AC**	**ICTI**	**All**	**TAU**	**AC**	**ICTI**	**All**	**TAU**	**AC**	**ICTI**
Median (IQR)	2.23 (1.67 - 2.67)	2.12 (1.67 - 2.48)	2.23 (1.67 - 2.67)	2.23 (1.75 - 2.78)	1.89 (1.56 - 2.23)	2.23 (2.00 - 2.34)	2.06 (1.56 - 2.51)	1.67 (1.45 - 1.92)	1.84 (1.45 - 2.34)	1.89 (1.56 - 2.45)	1.78 (1.34 - 2.12)	1.89 (1.56 - 2.23)	1.89 (1.56 - 2.34)	2.34 (1.56 - 2.78)	1.78 (1.42 - 2.12)	1.89 (1.67 - 2.23)
Min - Max	1 - 4	1 - 4	1 - 3	1 - 3	1 - 4	1 - 4	1 - 4	1 - 2	1 - 4	1 - 3	1 - 4	1 - 3	1 - 4	1 - 3	1 - 4	1 - 3
**Number of sick days taken during the past 4 weeks**																
N	99	20	39	40	67	17	26	24	64	19	24	21	66	17	24	25
Mean (SD)	1.98 (5.05)	1.65 (3.95)	2.79 (6.80)	1.35 (3.23)	1.66 (4.44)	0.06 (0.24)	1.31 (3.07)	3.17 (6.48)	1.56 (4.22)	1.32 (3.46)	1.21 (2.55)	2.19 (6.10)	1.06 (4.25)	0.59 (1.50)	2.33 (6.82)	0.16 (0.47)
Median (IQR)	0.00 (0.00 - 0.50)	0.00 (0.00 - 0.00)	0.00 (0.00 - 2.00)	0.00 (0.00 - 0.25)	0.00 (0.00 - 1.00)	0.00 (0.00 - 0.00)	0.00 (0.00 - 0.75)	0.50 (0.00 - 2.25)	0.00 (0.00 - 1.00)	0.00 (0.00 - 0.00)	0.00 (0.00 - 1.00)	0.00 (0.00 - 2.00)	0.00 (0.00 - 0.00)	0.00 (0.00 - 0.00)	0.00 (0.00 - 0.25)	0.00 (0.00 - 0.00)
Min - Max	0 - 28	0 - 14	0 - 28	0 - 14	0 - 28	0 - 1	0 - 12	0 - 28	0 - 28	0 - 14	0 - 10	0 - 28	0 - 28	0 - 6	0 - 28	0 - 2
****Impact of Intrusive Memories****																
**IMR Q1. Approximately how often did intrusive memories of the traumatic event pop into your mind?, n (%)**																
Never	0 (0.0%)	0 (0.0%)	0 (0.0%)	0 (0.0%)	5 (6.9%)	0 (0.0%)	1 (3.4%)	4 (16.0%)	20 (31.3%)	4 (21.1%)	5 (20.8%)	11 (52.4%)	19 (27.9%)	4 (22.2%)	1 (4.2%)	14 (53.8%)
Once	1 (1.0%)	1 (5.0%)	0 (0.0%)	0 (0.0%)	8 (11.1%)	2 (11.1%)	2 (6.9%)	4 (16.0%)	8 (12.5%)	5 (26.3%)	1 (4.2%)	2 (9.5%)	11 (16.2%)	1 (5.6%)	4 (16.7%)	6 (23.1%)
Twice	4 (4.0%)	1 (5.0%)	2 (5.1%)	1 (2.5%)	12 (16.7%)	2 (11.1%)	2 (6.9%)	8 (32.0%)	11 (17.2%)	4 (21.1%)	3 (12.5%)	4 (19.0%)	17 (25.0%)	7 (38.9%)	6 (25.0%)	4 (15.4%)
Every other day	31 (31.3%)	8 (40.0%)	12 (30.8%)	11 (27.5%)	22 (30.6%)	7 (38.9%)	12 (41.4%)	3 (12.0%)	11 (17.2%)	1 (5.3%)	7 (29.2%)	3 (14.3%)	15 (22.1%)	2 (11.1%)	11 (45.8%)	2 (7.7%)
Once a day	13 (13.1%)	4 (20.0%)	5 (12.8%)	4 (10.0%)	9 (12.5%)	3 (16.7%)	4 (13.8%)	2 (8.0%)	5 (7.8%)	3 (15.8%)	1 (4.2%)	1 (4.8%)	0 (0.0%)	0 (0.0%)	0 (0.0%)	0 (0.0%)
Several times a day	46 (46.5%)	3 (15.0%)	20 (51.3%)	23 (57.5%)	14 (19.4%)	3 (16.7%)	7 (24.1%)	4 (16.0%)	9 (14.1%)	2 (10.5%)	7 (29.2%)	0 (0.0%)	5 (7.4%)	4 (22.2%)	1 (4.2%)	0 (0.0%)
Many times a day	4 (4.0%)	3 (15.0%)	0 (0.0%)	1 (2.5%)	2 (2.8%)	1 (5.6%)	1 (3.4%)	0 (0.0%)	0 (0.0%)	0 (0.0%)	0 (0.0%)	0 (0.0%)	1 (1.5%)	0 (0.0%)	1 (4.2%)	0 (0.0%)
**IMR Q1a. How many times per day?***																
N	50	6	20	24	16	4	8	4	9	2	7	0	6	4	2	0
Mean (SD)	3.36 (2.10)	5.17 (2.48)	2.90 (1.12)	3.29 (2.44)	2.81 (1.11)	3.75 (1.71)	2.63 (0.74)	2.25 (0.50)	3.56 (1.51)	5.00 (1.41)	3.14 (1.35)	NA	3.50 (1.22)	3.25 (1.26)	4.00 (1.41)	NA

	Baseline				Week 4				Week 12				Week 24			
**Variable**	**All**	**TAU**	**AC**	**ICTI**	**All**	**TAU**	**AC**	**ICTI**	**All**	**TAU**	**AC**	**ICTI**	**All**	**TAU**	**AC**	**ICTI**
Median (IQR)	3.00 (2.00 - 4.00)	4.50 (4.00 - 5.00)	2.50 (2.00 - 3.25)	3.00 (2.00 - 3.25)	2.50 (2.00 - 3.00)	3.50 (2.75 - 4.50)	2.50 (2.00 - 3.00)	2.00 (2.00 - 2.25)	3.00 (3.00 - 4.00)	5.00 (4.50 - 5.50)	3.00 (2.50 - 3.00)	NA	3.00 (3.00 - 4.50)	3.00 (2.75 - 3.50)	4.00 (3.50 - 4.50)	NA
Min - Max	2 - 14	3 - 10	2 - 5	2 - 14	2 - 6	2 - 6	2 - 4	2 - 3	2 - 6	4 - 6	2 - 6	NA	2 - 5	2 - 5	3 - 5	NA
**IMR Q2. How distressing were your intrusive memories? (scale 0-10)**																
N	99	20	39	40	72	18	29	25	64	19	24	21	68	18	24	26
Mean (SD)	5.62 (1.75)	5.25 (1.62)	5.46 (1.50)	5.95 (2.00)	4.68 (2.19)	5.17 (1.79)	4.83 (1.98)	4.16 (2.61)	3.84 (3.02)	4.37 (3.00)	4.46 (2.81)	2.67 (3.06)	3.37 (2.79)	4.17 (3.11)	4.29 (2.26)	1.96 (2.51)
Median (IQR)	6.00 (4.50 - 7.00)	5.00 (4.75 - 6.25)	5.00 (4.00 - 6.50)	6.00 (5.00 - 7.00)	5.00 (3.75 - 6.00)	5.50 (4.00 - 6.75)	5.00 (4.00 - 6.00)	5.00 (3.00 - 6.00)	4.00 (0.00 - 6.00)	6.00 (2.00 - 7.00)	5.00 (3.00 - 6.00)	3.00 (0.00 - 5.00)	3.00 (0.00 - 5.25)	4.00 (2.25 - 6.75)	4.50 (3.00 - 6.00)	0.00 (0.00 - 3.75)
Min - Max	1 - 10	2 - 8	3 - 8	1 - 10	0 - 9	2 - 8	0 - 8	0 - 9	0 - 10	0 - 8	0 - 9	0 - 10	0 - 10	0 - 10	0 - 8	0 - 7
**IMR Q3. How much did they disrupt your concentration? (scale 0-10)**																
N	99	20	39	40	72	18	29	25	64	19	24	21	68	18	24	26
Mean (SD)	6.30 (2.33)	5.80 (2.42)	6.31 (2.31)	6.55 (2.32)	4.92 (2.43)	5.28 (2.14)	5.17 (2.45)	4.36 (2.60)	3.88 (3.19)	4.68 (3.23)	4.50 (3.02)	2.43 (2.98)	3.22 (2.99)	4.06 (3.57)	4.04 (2.39)	1.88 (2.66)
Median (IQR)	7.00 (5.00 - 8.00)	5.50 (4.00 - 8.00)	6.00 (5.00 - 8.00)	7.00 (5.75 - 8.00)	5.00 (4.00 - 7.00)	5.00 (4.00 - 6.00)	5.00 (4.00 - 7.00)	5.00 (3.00 - 6.00)	4.00 (0.00 - 6.25)	6.00 (1.50 - 6.50)	5.50 (2.00 - 7.00)	2.00 (0.00 - 4.00)	3.00 (0.00 - 6.00)	3.50 (0.50 - 6.75)	4.00 (2.00 - 6.00)	0.00 (0.00 - 4.00)
Min - Max	0 - 10	2 - 10	0 - 10	1 - 10	0 - 10	1 - 10	0 - 9	0 - 8	0 - 10	0 - 10	0 - 9	0 - 10	0 - 10	0 - 10	0 - 8	0 - 8
**IMR Q4. How much did they interfere with what you were doing? (scale 0-10)**																
N	99	20	39	40	72	18	29	25	64	19	24	21	68	18	24	26
Mean (SD)	5.58 (2.36)	4.65 (2.23)	5.44 (2.15)	6.18 (2.49)	4.29 (2.35)	4.44 (1.89)	4.59 (2.44)	3.84 (2.56)	3.25 (3.02)	4.05 (3.10)	3.67 (2.93)	2.05 (2.82)	2.72 (2.80)	3.22 (3.32)	3.63 (2.55)	1.54 (2.25)
Median (IQR)	6.00 (4.00 - 7.00)	5.00 (3.00 - 6.00)	6.00 (4.00 - 7.00)	6.00 (5.00 - 8.00)	4.00 (2.75 - 6.00)	4.50 (3.25 - 5.75)	5.00 (3.00 - 7.00)	4.00 (2.00 - 6.00)	3.00 (0.00 - 6.00)	4.00 (1.00 - 6.50)	3.00 (1.50 - 6.00)	0.00 (0.00 - 3.00)	2.00 (0.00 - 4.25)	2.50 (0.00 - 5.75)	4.00 (1.75 - 5.25)	0.00 (0.00 - 3.00)
Min - Max	0 - 10	1 - 9	0 - 10	1 - 10	0 - 9	1 - 9	0 - 9	0 - 8	0 - 10	0 - 9	0 - 9	0 - 10	0 - 10	0 - 10	0 - 8	0 - 7
**IMR Q4a. And for how long each time (approximately)?, n (%)**																
<1 min	43 (43.4%)	11 (55.0%)	12 (30.8%)	20 (50.0%)	36 (50.0%)	10 (55.6%)	10 (34.5%)	16 (64.0%)	43 (67.2%)	12 (63.2%)	13 (54.2%)	18 (85.7%)	41 (60.3%)	11 (61.1%)	9 (37.5%)	21 (80.8%)

	Baseline				Week 4				Week 12				Week 24			
**Variable**	**All**	**TAU**	**AC**	**ICTI**	**All**	**TAU**	**AC**	**ICTI**	**All**	**TAU**	**AC**	**ICTI**	**All**	**TAU**	**AC**	**ICTI**
1-5 mins	47 (47.5%)	8 (40.0%)	24 (61.5%)	15 (37.5%)	30 (41.7%)	8 (44.4%)	14 (48.3%)	8 (32.0%)	15 (23.4%)	5 (26.3%)	9 (37.5%)	1 (4.8%)	22 (32.4%)	6 (33.3%)	13 (54.2%)	3 (11.5%)
6-10 mins	9 (9.1%)	1 (5.0%)	3 (7.7%)	5 (12.5%)	4 (5.6%)	0 (0.0%)	3 (10.3%)	1 (4.0%)	6 (9.4%)	2 (10.5%)	2 (8.3%)	2 (9.5%)	4 (5.9%)	1 (5.6%)	1 (4.2%)	2 (7.7%)
11-30 mins	0 (0.0%)	0 (0.0%)	0 (0.0%)	0 (0.0%)	2 (2.8%)	0 (0.0%)	2 (6.9%)	0 (0.0%)	0 (0.0%)	0 (0.0%)	0 (0.0%)	0 (0.0%)	1 (1.5%)	0 (0.0%)	1 (4.2%)	0 (0.0%)
31-60 mins	0 (0.0%)	0 (0.0%)	0 (0.0%)	0 (0.0%)	0 (0.0%)	0 (0.0%)	0 (0.0%)	0 (0.0%)	0 (0.0%)	0 (0.0%)	0 (0.0%)	0 (0.0%)	0 (0.0%)	0 (0.0%)	0 (0.0%)	0 (0.0%)
>60 mins	0 (0.0%)	0 (0.0%)	0 (0.0%)	0 (0.0%)	0 (0.0%)	0 (0.0%)	0 (0.0%)	0 (0.0%)	0 (0.0%)	0 (0.0%)	0 (0.0%)	0 (0.0%)	0 (0.0%)	0 (0.0%)	0 (0.0%)	0 (0.0%)
**IMR Q5. How much did your intrusive memories affect your work functioning? (scale 0-10)**																
N	99	20	39	40	72	18	29	25	64	19	24	21	68	18	24	26
Mean (SD)	4.55 (2.60)	3.70 (2.62)	4.77 (2.45)	4.75 (2.72)	3.33 (2.72)	3.72 (2.59)	3.55 (2.75)	2.80 (2.81)	2.50 (3.19)	3.16 (3.73)	2.96 (3.14)	1.38 (2.50)	2.04 (2.80)	2.67 (3.33)	2.75 (3.01)	0.96 (1.78)
Median (IQR)	5.00 (3.00 - 6.50)	3.50 (1.75 - 5.25)	4.00 (3.00 - 6.00)	6.00 (2.00 - 7.00)	3.00 (1.00 - 6.00)	3.00 (2.00 - 6.00)	3.00 (2.00 - 5.00)	2.00 (0.00 - 5.00)	1.00 (0.00 - 5.00)	2.00 (0.00 - 6.00)	2.00 (0.00 - 6.00)	0.00 (0.00 - 2.00)	0.50 (0.00 - 3.00)	1.50 (0.00 - 4.00)	2.00 (0.00 - 4.25)	0.00 (0.00 - 1.75)
Min - Max	0 - 10	0 - 9	0 - 10	0 - 10	0 - 10	0 - 9	0 - 10	0 - 8	0 - 10	0 - 10	0 - 9	0 - 10	0 - 10	0 - 10	0 - 10	0 - 6
**IMR Q7. How much did your intrusive memories affect your functioning in other areas of your life (e.g. relationships with other people, parenting, social life, study, housework, voluntary work etc.) (scale 0-10)**																
N	99	20	39	40	72	18	29	25	64	19	24	21	68	18	24	26
Mean (SD)	5.04 (2.59)	4.80 (2.57)	4.72 (2.45)	5.48 (2.74)	3.76 (2.78)	4.17 (2.33)	4.21 (2.99)	2.96 (2.73)	2.72 (3.15)	3.79 (3.66)	2.96 (3.03)	1.48 (2.44)	1.84 (2.43)	2.22 (2.82)	2.50 (2.67)	0.96 (1.59)
Median (IQR)	5.00 (3.00 - 7.00)	5.00 (2.75 - 7.25)	4.00 (3.00 - 7.00)	5.50 (4.00 - 8.00)	3.00 (1.00 - 6.00)	4.50 (2.25 - 6.00)	4.00 (2.00 - 6.00)	2.00 (1.00 - 5.00)	1.00 (0.00 - 5.00)	3.00 (0.00 - 7.00)	2.00 (0.00 - 6.00)	0.00 (0.00 - 2.00)	1.00 (0.00 - 3.00)	1.00 (0.00 - 3.75)	2.00 (0.00 - 3.25)	0.00 (0.00 - 1.75)
Min - Max	0 - 10	1 - 8	0 - 10	0 - 10	0 - 10	0 - 8	0 - 10	0 - 9	0 - 10	0 - 10	0 - 9	0 - 8	0 - 10	0 - 9	0 - 10	0 - 5
**IMR Q9. On how many days did you work this week?**																
N	99	20	39	40	72	18	29	25	64	19	24	21	68	18	24	26
Mean (SD)	3.46 (1.81)	3.95 (1.47)	3.00 (1.91)	3.68 (1.80)	3.32 (1.83)	3.67 (1.50)	3.28 (2.15)	3.12 (1.67)	3.45 (1.65)	3.68 (1.53)	3.00 (1.62)	3.76 (1.76)	2.99 (1.97)	3.28 (2.08)	2.92 (1.93)	2.85 (1.99)
Median (IQR)	4.00 (2.50 - 5.00)	4.00 (3.00 - 5.00)	3.00 (1.50 - 4.50)	4.00 (3.00 - 5.00)	3.50 (2.00 - 5.00)	4.00 (3.00 - 5.00)	4.00 (2.00 - 5.00)	3.00 (3.00 - 5.00)	4.00 (3.00 - 5.00)	4.00 (3.00 - 5.00)	3.00 (2.75 - 4.00)	4.00 (3.00 - 5.00)	3.00 (1.00 - 5.00)	4.00 (1.25 - 5.00)	3.50 (1.50 - 4.25)	3.00 (1.25 - 4.00)

	Baseline				Week 4				Week 12				Week 24			
**Variable**	**All**	**TAU**	**AC**	**ICTI**	**All**	**TAU**	**AC**	**ICTI**	**All**	**TAU**	**AC**	**ICTI**	**All**	**TAU**	**AC**	**ICTI**
Min - Max	0 - 7	0 - 6	0 - 7	0 - 6	0 - 7	0 - 6	0 - 7	0 - 5	0 - 7	0 - 5	0 - 5	0 - 7	0 - 7	0 - 6	0 - 5	0 - 7
**IMR Q10. How many times did you do a night shift this week?**																
N	99	20	39	40	72	18	29	25	64	19	24	21	68	18	24	26
Mean (SD)	0.30 (0.96)	0.50 (1.00)	0.36 (1.29)	0.15 (0.43)	0.15 (0.57)	0.17 (0.71)	0.00 (0.00)	0.32 (0.75)	0.27 (0.80)	0.26 (0.81)	0.29 (0.86)	0.24 (0.77)	0.37 (1.04)	0.61 (1.46)	0.13 (0.61)	0.42 (0.99)
Median (IQR)	0.00 (0.00 - 0.00)	0.00 (0.00 - 0.25)	0.00 (0.00 - 0.00)	0.00 (0.00 - 0.00)	0.00 (0.00 - 0.00)	0.00 (0.00 - 0.00)	0.00 (0.00 - 0.00)	0.00 (0.00 - 0.00)	0.00 (0.00 - 0.00)	0.00 (0.00 - 0.00)	0.00 (0.00 - 0.00)	0.00 (0.00 - 0.00)	0.00 (0.00 - 0.00)	0.00 (0.00 - 0.00)	0.00 (0.00 - 0.00)	0.00 (0.00 - 0.00)
Min - Max	0 - 7	0 - 3	0 - 7	0 - 2	0 - 3	0 - 3	0 - 0	0 - 3	0 - 3	0 - 3	0 - 3	0 - 3	0 - 5	0 - 5	0 - 3	0 - 3

Note. Descriptive statistics of secondary outcome measures - excluding number of IMs recorded in the IM diary at weeks 12 and 24 (see Table S1) and adverse events (see Table S23) - are presented at baseline and follow-up (weeks 4, 12 and 24). In addition, descriptive statistics for post-hoc analysis of PCL-5 subgroups (i.e., participants with and without probable PTSD at baseline) and the four PTSD symptom clusters (re-experiencing, avoidance, negative alterations, and hyper-arousal) of the PCL-5 are presented.

*Conditional item, only presented to participant for a response of "Many times a day" or "Several times a day" to the previous item (IMR Q1 "Approximately how often did IMs of the traumatic event pop into your mind?"). If no participants selected these response options, item IMR Q1a is not applicable.

Abbreviations. SD = standard deviation; IQR = interquartile range; ICTI = Imagery-Competing Task Intervention; AC = active control; TAU = treatment-as-usual; PCL-5 = PTSD Checklist for DSM-5 (20-item version)[3], SCI-2 = sleep condition indicator (2-item version)[4]; GAD-2 = Generalised Anxiety Disorder Assessment (2-item version)[5]; PHQ-2 = Patient Health Questionnaire (2-item version)[6]; SWEBO = Scale of Work Engagement and Burnout[7]; WHODAS 2.0 = World Health Organization Disability Assessment Schedule 12-item version[8]; EQ-5D-5L = European Quality of Life Five Dimension Five Level Scale[9]; VAS = Visual Analogue Scale; ITL = Intention to Leave the Job Scale[10]; IMR = intrusive memory rating scale[11]; NA = not applicable.

Table S7. Secondary outcomes: PTSD symptom severity at all time-points. Bayesian results of between groups comparisons

Timepoint	Ref. Group	Ref. N	Comp. Group	Comp. N	Ref. Mean (SD)	Comp. Mean (SD)	Comp. vs Ref. Estimate	Lower 95% CrI	Upper 95% CrI	BF (ICTI < Comp)	BF (ICTI > Comp)	Pr (ICTI ≤ Comp. – Δ)
Baseline	ICTI	26	AC	30	28.692 (17.368)	32.167 (18.287)						
Baseline	ICTI	26	TAU	19	28.692 (17.368)	33.526 (12.946)						
Week 4	ICTI	25	AC	27	13.840 (8.863)	26.852 (17.099)	9.884	4.003	15.693	1545.6667	0.0006	0.95
Week 4	ICTI	25	TAU	17	13.840 (8.863)	30.941 (14.184)	12.625	6.121	19.095	7732.3333	0.0001	0.99
Week 12	ICTI	21	AC	24	10.857 (10.489)	19.875 (16.177)	7.683	1.399	13.960	117.0061	0.0085	0.80
Week 12	ICTI	21	TAU	19	10.857 (10.489)	23.789 (15.701)	9.623	2.717	16.409	304.2632	0.0033	0.91
Week 24	ICTI	26	AC	24	8.423 (8.324)	16.083 (11.891)	7.463	1.353	13.560	118.5876	0.0084	0.79
Week 24	ICTI	26	TAU	17	8.423 (8.324)	21.294 (15.830)	8.966	2.088	15.727	176.3700	0.0057	0.87

Note. Secondary Bayesian analysis of PTSD symptom scores measured using the PCL-5 (20-item version) at weeks 4, 12 and 24 was modelled via a linear regression model with a normal distribution.

Bayes factors (BF) quantifying the evidence for an alternative hypothesis (H_1) over the null hypothesis (H0) are presented to illustrate evidence of the ICTI (reference group) having greater or lower PCL-5 total score relative to the AC and TAU arms (comparison groups). The estimate shows the expected difference in the model between the ICTI and comparator arms. Positive estimates > 0 show model estimated PTSD symptom severity scores were greater in the comparator arms relative to the ICTI arm. Means (SD) are presented at each time-point for the analysis population included in the model. Pr(ICTI≤Comp. - Δ) is the estimated posterior probability of at least five fewer IMs in ICTI than the Comp Group, where Δ = 5 representing a clinically important difference in PCL-5 total score.

Abbreviations: PCL-5 = PTSD Checklist for DSM-5 (20-item version)*;* ICTI = Imagery-Competing Task Intervention; AC = active control; TAU = treatment-as-usual; Ref. = reference [group]; Comp. = comparator [group]; SD = standard deviation; CrI = credible interval; BF = Bayes factor.

Figure S9. Secondary outcomes: PTSD symptom severity at all time-points. Sensitivity analysis with varying priors.

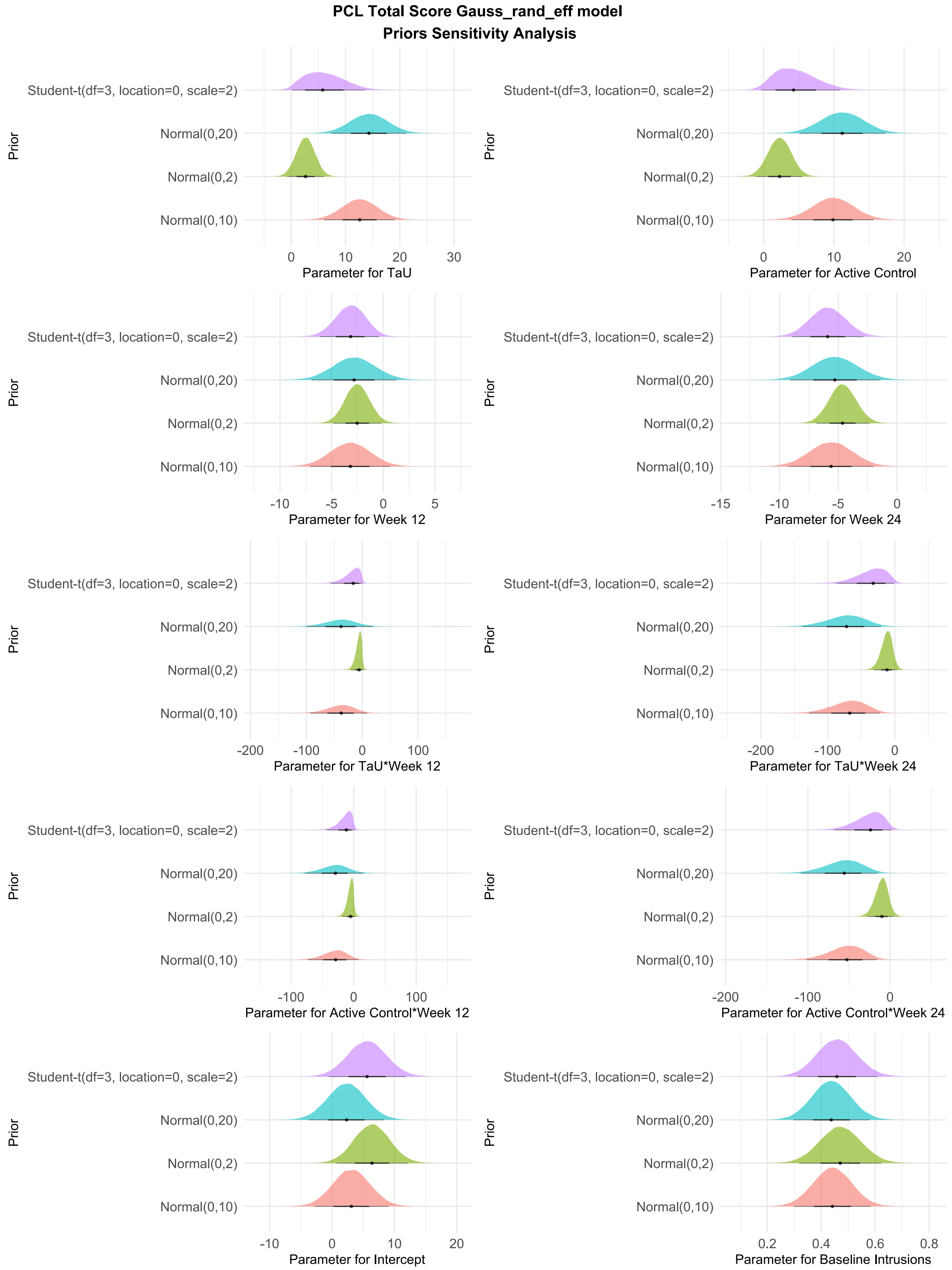

Figure S10A. Post-hoc analysis: PCL-5 subgroup sensitivity analysis with priors

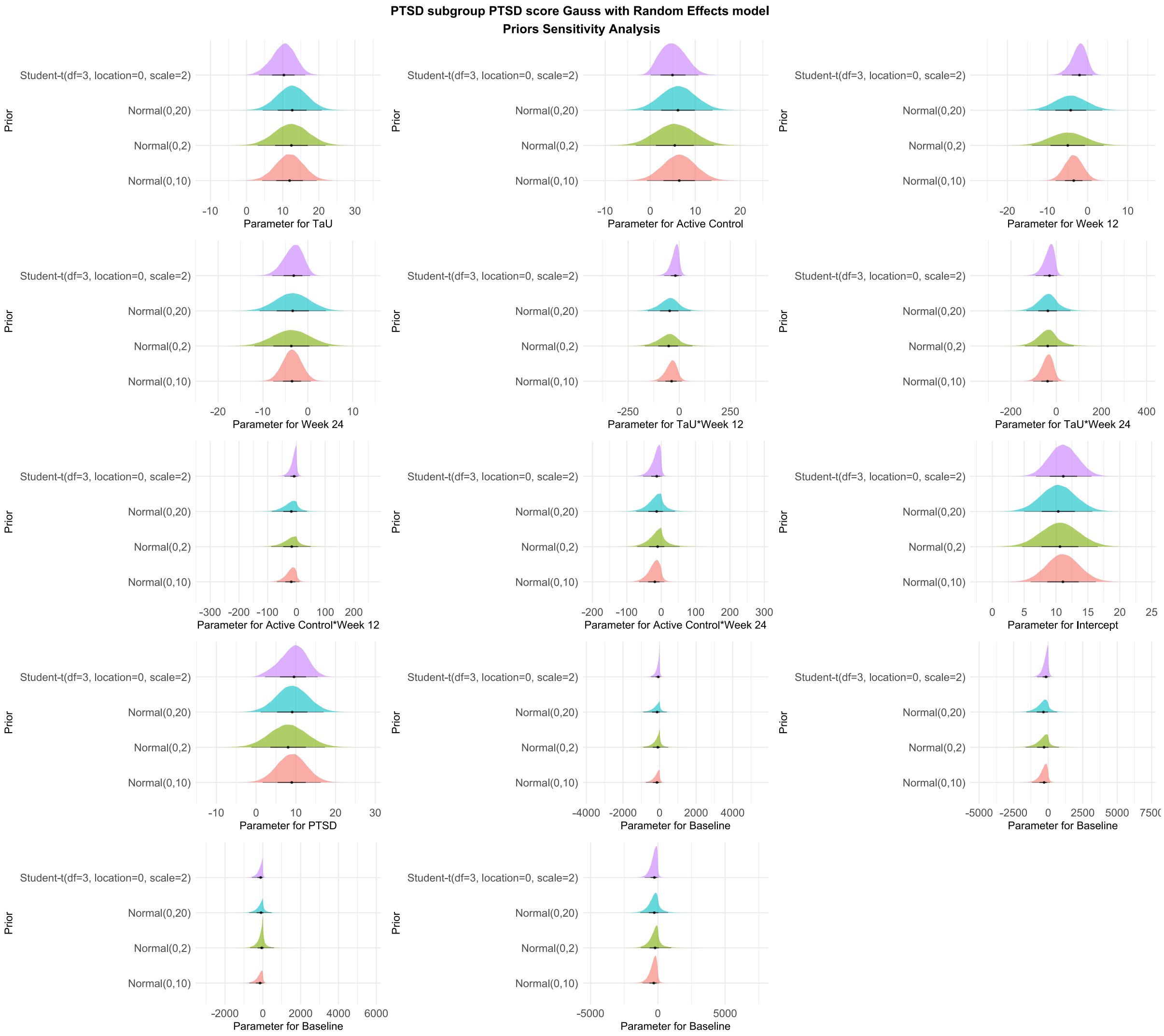

Figure S10B. Post-hoc analysis: PTSD Symptom Severity (PCL-5 total) Across Time-Points per Arm and subgroup (with [baseline PCL-5 ≥33] or without [baseline PCL-5 <33] probable PTSD).

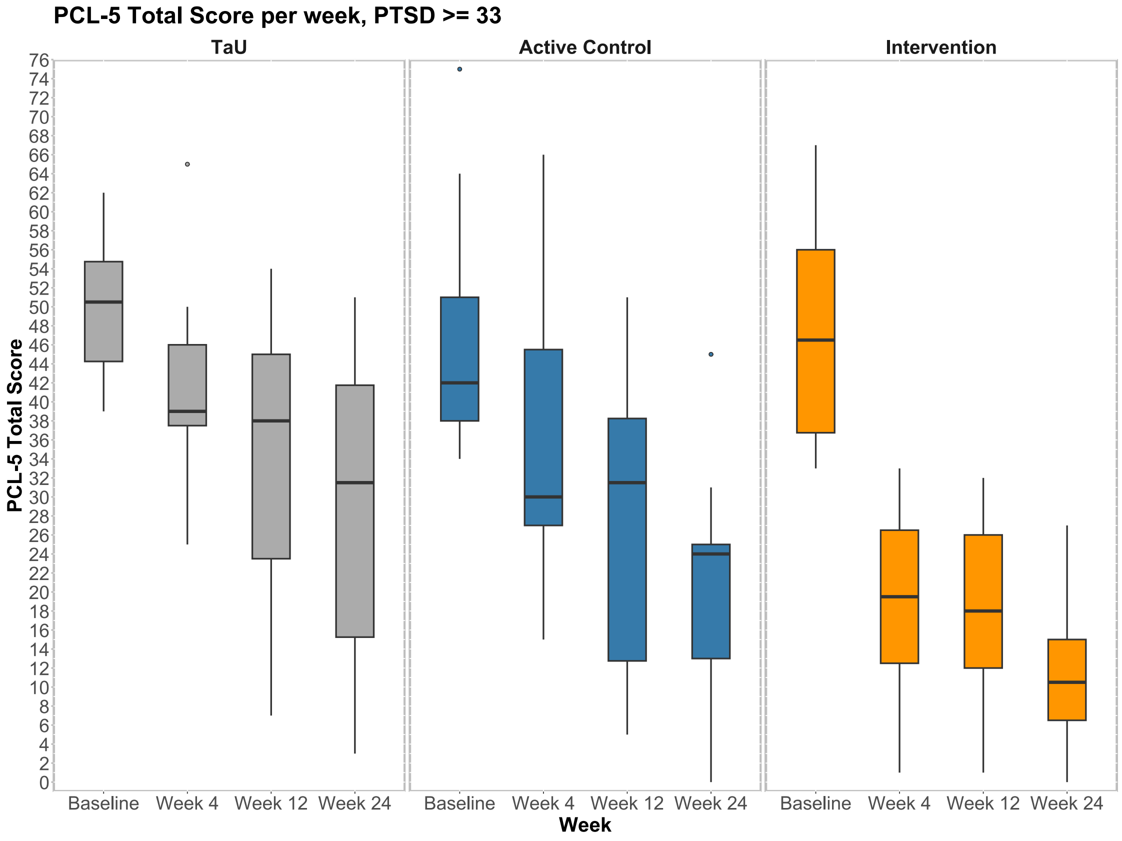

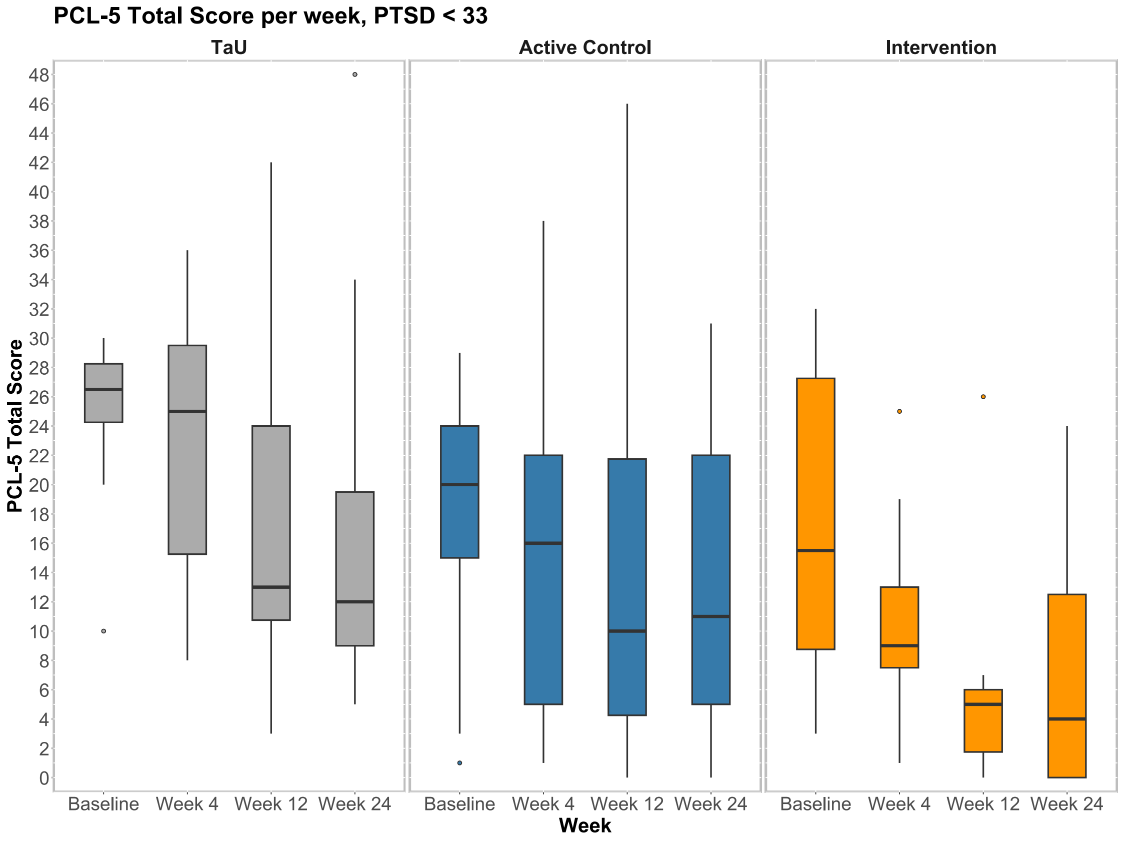

Table S8. Post-hoc analysis. Benefits of ICTI on PTSD symptom severity for those meeting criteria for PTSD.

For post-hoc analysis, subgroups were created according to baseline PCL-5 total scores. Following criteria outlined by Weathers et al. (2013), PCL-5 total scores ≥33 at baseline were considered indicative of probable PTSD. Supplementary Tables 8a & 8b present post-hoc Bayesian analysis of PTSD symptom scores at weeks 4, 12 and 24 for sub-groups of participants without and with probable PTSD at baseline respectively. Analyses employed a Bayesian linear mixed model, with identity link function, with arm, week, and subgroup with fixed effects, including all interactions and subject as a random effect. Baseline scores were not included in the model as they were used to determine sub-group allocation.

Table S8A. Benefits of ICTI on PTSD symptom severity for subgroup without probable PTSD at baseline (<33 PCL-5). Bayesian results of between groups comparisons.

Timepoint	Ref. Group	Ref. N	Comp. Group	Comp. N	Ref. Mean (SD)	Comp. Mean (SD)	Comp. vs Ref. Estimate	Lower 95% CrI	Upper 95% CrI	BF (ICTI < Comp)	BF (ICTI > Comp)
Week 4	ICTI	15	AC	13	10.733 (5.946)	16.000 (12.623)	6.446	-0.743	13.611	24.4721	0.0409
Week 4	ICTI	15	TAU	10	10.733 (5.946)	23.000 (9.165)	11.900	4.313	19.399	710.6564	0.0014
Week 12	ICTI	12	AC	14	5.583 (6.868)	14.214 (14.034)	6.293	-1.659	14.210	15.7364	0.0635
Week 12	ICTI	12	TAU	12	5.583 (6.868)	18.000 (12.121)	9.977	1.661	18.234	105.4220	0.0095
Week 24	ICTI	16	AC	15	6.938 (8.282)	13.067 (10.271)	5.472	-2.180	13.069	11.4210	0.0876
Week 24	ICTI	16	TAU	11	6.938 (8.282)	17.273 (13.146)	8.523	0.357	16.712	47.4342	0.0211

Note. Bayes factors (BF) quantifying the evidence for an alternative hypothesis (H_1) over a null hypothesis (H_0) are presented to illustrate evidence in favour of positive and negative treatment effects of ICTI for PTSD symptom severity at all follow-up time-points (weeks 4, 12 and 24) for the subgroup without probable PTSD at baseline (PCL-5 total score at baseline <33). The ICTI arm is treated as the reference group with the AC and TAU arms treated as the comparators. The Estimate shows the expected difference in the model between the ICTI and respective comparator arm. Positive estimates > 0 show model estimated PTSD symptom severity scores were greater in the comparator arms relative to the ICTI. Means (SD) for the subgroup with PCL-5 total score <33 at baseline analysed in the Bayesian model are presented at each time-point.

Abbreviations: PTSD = post-traumatic stress disorder; PCL-5 = PTSD Checklist for DSM-5 (20-item version); ICTI = Imagery-Competing Task Intervention; AC = active control; TAU = treatment-as-usual; Ref. = reference [group]; Comp. = comparator [group]; SD = standard deviation; CrI = credible interval; BF = Bayes factor.

Table S8B. Benefits of ICTI on PTSD symptom severity for subgroup with probable PTSD at baseline (≥33 PCL-5). Bayesian results of between groups comparisons.

Timepoint	Ref. Group	Ref. N	Comp. Group	Comp. N	Ref. Mean (SD)	Comp. Mean (SD)	Comp. vs Ref. Estimate	Lower 95% CrI	Upper 95% CrI	BF (ICTI < Comp)	BF (ICTI > Comp)
Week 4	ICTI	10	AC	14	18.500 (10.690)	36.929 (14.510)	23.751	15.688	31.710	2973.3590	0.0003
Week 4	ICTI	10	TAU	7	18.500 (10.690)	42.286 (12.459)	29.138	19.031	39.004	28999.000	<0.0001
Week 12	ICTI	9	AC	10	17.889 (10.624)	27.800 (16.267)	18.298	8.557	27.939	36.9209	0.0271
Week 12	ICTI	9	TAU	7	17.889 (10.624)	33.714 (16.978)	23.800	12.509	34.935	246.8632	0.0041
Week 24	ICTI	10	AC	9	10.800 (8.244)	21.111 (13.280)	18.968	9.129	28.688	51.4887	0.0194
Week 24	ICTI	10	TAU	6	10.800 (8.244)	28.667 (18.854)	24.321	12.862	35.639	294.1654	0.0034

Note. Bayes factors (BF) quantifying the evidence for an alternative hypothesis (H_1) over the null hypothesis (H0) are presented to illustrate evidence in favour of positive and negative treatment effects of the ICTI for PTSD symptom severity at all follow-up time-points (weeks 4, 12 and 24) for the subgroup with probable PTSD at baseline (PCL-5 total score at baseline ≥33). The ICTI arm is treated as the reference group with the AC and TAU arms treated as the comparators. The estimate shows the expected difference in the model between the ICTI and respective comparator arm. Positive estimates > 0 show model estimated PTSD symptom severity scores were greater in the comparator arms relative to the ICTI. Means (SD) for the subgroup with PCL-5 total score ≥33 at baseline analysed in the Bayesian model are presented at each time-point.

Abbreviations: PTSD = post-traumatic stress disorder; PCL-5 = PTSD Checklist for DSM-5 (20-item version); ICTI = Imagery-Competing Task Intervention; AC = active control; TAU = treatment-as-usual; Ref. = reference [group]; Comp. = comparator [group]; SD = standard deviation; CrI = credible interval; BF = Bayes factor.

Table S8C. Post-hoc analysis. Benefits of ICTI on PTSD symptom severity overall and within PTSD subgroups and arms with estimated Cohen's d effect size.

Comparison	Week	Cohen's d	Lower 95% CI	Upper 95% CI
**Overall (whole sample)**				
ICTI vs AC	Week 4	0.94	0.36	1.53
ICTI vs TAU	Week 4	1.51	0.80	2.23
ICTI vs AC	Week 12	0.65	0.03	1.27
ICTI vs TAU	Week 12	0.98	0.30	1.66
ICTI vs AC	Week 24	0.75	0.16	1.34
ICTI vs TAU	Week 24	1.09	0.41	1.76
**With probable PTSD at baseline (PCL-5 total score ≥33)**				
ICTI vs AC	Week 4	1.41	0.45	2.37
ICTI vs TAU	Week 4	2.08	0.78	3.38
ICTI vs AC	Week 12	0.71	-0.29	1.71
ICTI vs TAU	Week 12	1.15	-0.01	2.32
ICTI vs AC	Week 24	0.95	-0.08	1.97
ICTI vs TAU	Week 24	1.37	0.14	2.59
**Without probable PTSD at baseline (PCL-5 total score <33)**				
ICTI vs AC	Week 4	0.55	-0.25	1.34
ICTI vs TAU	Week 4	1.66	0.69	2.64
ICTI vs AC	Week 12	0.76	-0.08	1.60
ICTI vs TAU	Week 12	1.26	0.33	2.19
ICTI vs AC	Week 24	0.66	-0.10	1.41
ICTI vs TAU	Week 24	0.98	0.13	1.84

Note. Cohen's d estimated difference in PCL-5 total scores between study arms and sub-groups (comparator minus ICTI) of participants with and without probable PTSD at Weeks 4, 12 and 24 for the whole sample (overall). We also calculated the estimated Cohen's d effect size PCL-5 total scores for the subgroups of participants with and without probable PTSD at baseline (PCL-5 Total Score ≥33) as a post-hoc analysis.

Abbreviations. ICTI = Imagery-Competing Task Intervention arm; AC = active control arm; TAU = treatment-as-usual; PTSD = post-traumatic stress disorder; CI = confidence interval.

Table S9. Post-hoc analysis. Benefits of ICTI on PTSD symptom severity for symptom clusters of PTSD. Bayesian results of between groups comparisons.

The PCL-5 consists of 4 subscales (avoidance, hyper-arousal, negative alterations, and re-experiencing) aligning with the four DSM-5 symptom clusters of PTSD. For each subscale, we conducted post-hoc Bayesian analysis using a linear regression model with a normal distribution based on a similar framework to the analysis of the PCL total score as detailed above.

Table S9A. Benefits of ICTI on PTSD symptom severity for symptom clusters of PTSD: *Avoidance.*

Timepoint	Ref. Group	Ref. N	Comp. Group	Comp. N	Ref. Mean (SD)	Comp. Mean (SD)	Comp. vs Ref. Estimate	Lower 95% CrI	Upper 95% CrI	BF (ICTI < Comp)	BF (ICTI > Comp)
Baseline	ICTI	26	AC	30	4.000 (2.638)	4.233 (2.388)					
Baseline	ICTI	26	TAU	19	4.000 (2.638)	4.684 (1.600)					
Week 4	ICTI	25	AC	27	2.040 (2.131)	3.333 (2.130)	1.195	0.139	2.252	74.4717	0.0134
Week 4	ICTI	25	TAU	17	2.040 (2.131)	3.588 (2.347)	1.304	0.101	2.510	59.3224	0.0169
Week 12	ICTI	21	AC	24	1.143 (1.682)	3.083 (2.636)	1.974	0.846	3.093	2415.6667	0.0004
Week 12	ICTI	21	TAU	19	1.143 (1.682)	3.316 (2.262)	1.954	0.743	3.163	1302.3708	0.0008
Week 24	ICTI	26	AC	24	1.308 (1.594)	2.500 (2.377)	1.323	0.247	2.405	125.9147	0.0079
Week 24	ICTI	26	TAU	17	1.308 (1.594)	2.941 (2.536)	1.357	0.160	2.554	75.1155	0.0133

Table S9B. Benefits of ICTI on PTSD symptom severity for symptom clusters of PTSD: *Re-experiencing.*

Timepoint	Ref. Group	Ref. N	Comp. Group	Comp. N	Ref. Mean (SD)	Comp. Mean (SD)	Comp. vs Ref. Estimate	Lower 95% CrI	Upper 95% CrI	BF (ICTI < Comp)	BF (ICTI > Comp)
Baseline	ICTI	26	AC	30	8.038 (4.521)	8.400 (4.839)					
Baseline	ICTI	26	TAU	19	8.038 (4.521)	8.579 (4.073)					
Week 4	ICTI	25	AC	27	3.440 (2.740)	6.741 (4.311)	3.146	1.283	5.027	1704.8824	0.0006
Week 4	ICTI	25	TAU	17	3.440 (2.740)	7.000 (4.809)	3.254	1.140	5.359	804.5556	0.0012
Week 12	ICTI	21	AC	24	2.524 (3.326)	5.125 (4.562)	2.559	0.581	4.543	187.3117	0.0053
Week 12	ICTI	21	TAU	19	2.524 (3.326)	5.263 (4.433)	2.454	0.316	4.586	76.8523	0.0130
Week 24	ICTI	26	AC	24	1.923 (2.189)	4.125 (3.542)	2.377	0.466	4.295	132.6406	0.0075
Week 24	ICTI	26	TAU	17	1.923 (2.189)	4.176 (4.187)	1.804	-0.323	3.899	19.7774	0.0506

Table S9C. Benefits of ICTI on PTSD symptom severity for symptom clusters of PTSD: *Hyper-arousal*

Timepoin t	Ref. Group	Ref. N	Comp. Group	Comp. N	Ref. Mean (SD)	Comp. Mean (SD)	Comp. vs Ref. Estimate	Lower 95% CrI	Upper 95% CrI	BF (ICTI < Comp)	BF (ICTI > Comp)
Baseline	ICTI	26	AC	30	7.462 (5.750)	8.900 (5.821)					
Baseline	ICTI	26	TAU	19	7.462 (5.750)	8.158 (4.598)					
Week 4	ICTI	25	AC	27	3.920 (3.121)	7.593 (5.458)	2.969	1.093	4.838	742.5897	0.0013
Week 4	ICTI	25	TAU	17	3.920 (3.121)	8.529 (4.502)	4.420	2.317	6.527	23199.0000	<0.0001
Week 12	ICTI	21	AC	24	3.667 (4.078)	4.833 (4.040)	0.852	-1.127	2.826	4.0761	0.2453
Week 12	ICTI	21	TAU	19	3.667 (4.078)	6.947 (5.421)	2.966	0.851	5.098	281.9268	0.0035
Week 24	ICTI	26	AC	24	2.654 (2.925)	4.333 (3.306)	1.746	-0.167	3.659	26.5469	0.0377
Week 24	ICTI	26	TAU	17	2.654 (2.925)	6.765 (5.166)	3.436	1.329	5.556	1273.7253	0.0008

Table S9D. Benefits of ICTI on PTSD symptom severity for symptom clusters of PTSD: ***Negative alterations***

Timepoint	Ref. Group	Ref. N	Comp. Group	Comp. N	Ref. Mean (SD)	Comp. Mean (SD)	Comp. vs Ref. Estimate	Lower 95% CrI	Upper 95% CrI	BF (ICTI < Comp)	BF (ICTI > Comp)
Baseline	ICTI	26	AC	30	9.192 (6.940)	10.633 (6.960)					
Baseline	ICTI	26	TAU	19	9.192 (6.940)	12.105 (5.962)					
Week 4	ICTI	25	AC	27	4.440 (2.959)	9.185 (6.873)	4.172	1.676	6.654	1525.3158	0.0007
Week 4	ICTI	25	TAU	17	4.440 (2.959)	11.824 (6.464)	5.818	2.980	8.643	57999.0000	<0.0001
Week 12	ICTI	21	AC	24	3.524 (3.341)	6.833 (6.397)	3.203	0.572	5.815	115.4659	0.0087
Week 12	ICTI	21	TAU	19	3.524 (3.341)	8.263 (6.410)	3.473	0.602	6.331	108.7446	0.0092
Week 24	ICTI	26	AC	24	2.538 (2.760)	5.125 (4.523)	2.874	0.338	5.401	73.2163	0.0137
Week 24	ICTI	26	TAU	17	2.538 (2.760)	7.412 (6.215)	3.635	0.795	6.495	151.2310	0.0066

Note. Bayes factors (BF) quantifying the evidence for an alternative hypothesis (H_1) over the null hypothesis (H_0) are presented to illustrate evidence in favour of positive and negative treatment effects of the ICTI for PTSD symptom cluster scores at all follow-up time-points (weeks 4, 12 and 24). The ICTI arm is treated as the reference group with the AC and TAU arms treated as the comparators. The estimate shows the expected difference in the model between the ICTI and respective comparator arm. Positive estimates > 0 show model estimated symptom cluster severity scores were greater in the comparator arms relative to the ICTI. Means (SD) are presented at each time-point for each symptom clusters.

Abbreviations: PTSD = post-traumatic stress disorder; PCL-5 = PTSD Checklist for DSM-5 (20-item version); ICTI = Imagery-Competing Task Intervention; AC = active control; TAU = treatment-as-usual; Ref. = reference [group]; Comp. = comparator [group]; SD = standard deviation; CrI = credible interval; BF = Bayes factor.

Table S10. Secondary outcomes: Other clinical outcomes, insomnia (SCI-2). Bayesian results of between groups comparisons.

Timepoint	Ref. Group	Ref. N	Comp. Group	Comp. N	Ref. Mean (SD)	Comp. Mean (SD)	Comp. vs Ref. Estimate	Lower 95% CrI	Upper 95% CrI	BF (ICTI < Comp)	BF (ICTI > Comp)
Baseline	ICTI	25	AC	30	2.920 (2.431)	3.433 (2.661)					
Baseline	ICTI	25	TAU	19	2.920 (2.431)	2.526 (2.342)					
Week 4	ICTI	24	AC	27	3.958 (2.274)	3.926 (2.934)	-0.707	-2.306	0.876	0.2308	4.3321
Week 4	ICTI	24	TAU	17	3.958 (2.274)	2.824 (2.811)	-1.623	-3.514	0.195	0.0427	23.4108
Week 12	ICTI	21	AC	24	4.952 (2.519)	4.833 (2.461)	-1.148	-2.851	0.512	0.0975	10.2523
Week 12	ICTI	21	TAU	19	4.952 (2.519)	3.737 (2.579)	-1.382	-3.186	0.372	0.0648	15.4236
Week 24	ICTI	25	AC	24	5.320 (2.479)	4.417 (2.717)	-1.649	-3.309	-0.031	0.0235	42.5435
Week 24	ICTI	25	TAU	17	5.320 (2.479)	3.706 (2.867)	-2.003	-3.849	-0.242	0.0132	75.9231

Note. Secondary Bayesian analysis of insomnia symptom scores measured using the SCI-2 (2-item version) at weeks 4, 12 and 24 was modelled via a cumulative regression model with logit link function for ordinal outcomes. The scale is scored such that lower total scores are indicative of more severe insomnia symptom severity.

Bayes factors (BF) quantifying the evidence for an alternative hypothesis (H_1) over the null hypothesis (H_0) are presented to illustrate evidence in favour of positive and negative treatment effects of the ICTI for SCI-2 total scores at all follow-up time-points (weeks 4, 12 and 24). The ICTI arm is treated as the reference group with the AC and TAU arms treated as the comparators. The Estimate shows the expected difference between the groups in the model. Negative estimates < 0 show model estimated insomnia severity scores were greater (i.e. worse insomnia) in the comparator arms relative to the ICTI. Means (SD) are presented at each time-point for the analysis population included in the model.

Abbreviations: SCI-2 = Sleep Condition Indicator (2-item version); ICTI = Imagery-Competing Task Intervention; AC = active control; TAU = treatment-as-usual; Ref. = reference [group]; Comp. = comparator [group]; SD = standard deviation; CrI = credible interval; BF = Bayes factor.

Table S11. Secondary outcomes: Other clinical outcomes, anxiety (GAD-2). Bayesian results of between groups comparisons.

Timepoint	Ref. Group	Ref. N	Comp. Group	Comp. N	Ref. Mean (SD)	Comp. Mean (SD)	Comp. vs Ref. Estimate	Lower 95% CrI	Upper 95% CrI	BF (ICTI < Comp)	BF (ICTI > Comp)
Baseline	ICTI	25	AC	30	2.520 (1.828)	2.533 (1.852)					
Baseline	ICTI	25	TAU	19	2.520 (1.828)	2.737 (2.104)					
Week 4	ICTI	24	AC	27	1.625 (1.279)	2.333 (1.754)	1.219	-0.411	2.870	13.3511	0.0749
Week 4	ICTI	24	TAU	17	1.625 (1.279)	2.529 (1.700)	1.859	0.117	3.669	53.4090	0.0187
Week 12	ICTI	21	AC	24	1.333 (1.278)	1.958 (1.781)	0.948	-0.786	2.714	6.0624	0.1650
Week 12	ICTI	21	TAU	19	1.333 (1.278)	2.684 (2.029)	2.353	0.516	4.262	161.2378	0.0062
Week 24	ICTI	25	AC	24	1.160 (1.106)	1.500 (1.383)	0.734	-0.947	2.440	4.1793	0.2393
Week 24	ICTI	25	TAU	17	1.160 (1.106)	2.059 (1.749)	1.571	-0.249	3.446	21.0574	0.0475

Note. Secondary Bayesian analysis of anxiety symptom scores measured using the GAD-2 (2-item version) at weeks 4, 12 and 24 was modelled via a cumulative regression model with logit link function for ordinal outcomes.

Bayes factors (BF) quantifying the evidence for an alternative hypothesis (H_1) over the null hypothesis (H_0) are presented to illustrate evidence in favour of positive and negative treatment effects of the ICTI for GAD-2 total scores at all follow-up time-points (weeks 4, 12 and 24). The ICTI arm is treated as the reference group with the AC and TAU arms treated as the comparators. The estimate shows the expected difference between the groups in the model. Positive estimates > 0 show model estimated anxiety severity scores were greater in the comparator arms relative to the ICTI. Means (SD) are presented at each time-point for the analysis population included in the model.

Abbreviations: GAD-2 = Generalised Anxiety Disorder Assessment (2-item version); ICTI = Imagery-Competing Task Intervention; AC = active control; TAU = treatment-as-usual; Ref. = reference [group]; Comp. = comparator [group]; SD = standard deviation; CrI = credible interval; BF = Bayes factor.

Table S12. Secondary outcomes: Other clinical outcomes, depression (PHQ-2). Bayesian results of between groups comparisons.

Timepoint	Ref. Group	Ref. N	Comp. Group	Comp. N	Ref. Mean (SD)	Comp. Mean (SD)	Comp. vs Ref. Estimate	Lower 95% CrI	Upper 95% CrI	BF (ICTI < Comp)	BF (ICTI > Comp)
Baseline	ICTI	25	AC	30	1.840 (1.344)	1.733 (1.413)					
Baseline	ICTI	25	TAU	19	1.840 (1.344)	2.053 (1.810)					
Week 4	ICTI	24	AC	27	0.833 (0.816)	1.630 (1.363)	2.044	0.262	3.903	80.1189	0.0125
Week 4	ICTI	24	TAU	17	0.833 (0.816)	2.176 (2.215)	2.347	0.302	4.469	77.6441	0.0129
Week 12	ICTI	21	AC	24	1.000 (1.000)	1.375 (1.740)	0.750	-1.151	2.674	3.5859	0.2789
Week 12	ICTI	21	TAU	19	1.000 (1.000)	1.789 (1.653)	1.079	-0.994	3.172	5.5838	0.1791
Week 24	ICTI	25	AC	24	0.760 (1.012)	1.042 (1.268)	0.663	-1.230	2.582	3.0988	0.3227
Week 24	ICTI	25	TAU	17	0.760 (1.012)	1.471 (1.505)	1.135	-0.964	3.269	6.0150	0.1663

Note. Secondary Bayesian analysis of depression symptom scores measured using the PHQ-2 (2-item version) at weeks 4, 12 and 24 was modelled via a cumulative regression model with logit link function for ordinal outcomes.

Bayes factors (BF) quantifying the evidence for an alternative hypothesis (H_1) over the null hypothesis (H_0) are presented to illustrate evidence in favour of positive and negative treatment effects of the ICTI for PHQ-2 total scores at all follow-up time-points (weeks 4, 12 and 24). The ICTI arm is treated as the reference group with the AC and TAU arms treated as the comparators. The estimate shows the expected difference between the groups in the model. Positive estimates > 0 show model estimated depression severity scores were greater in the comparator arms relative to the ICTI. Means (SD) are presented at each time-point for the analysis population included in the model.

Abbreviations: PHQ-2 = Patient Health Questionnaire (2-item version); ICTI = Imagery-Competing Task Intervention; AC = active control; TAU = treatment-as-usual; Ref. = reference [group]; Comp. = comparator [group]; SD = standard deviation; CrI = credible interval; BF = Bayes factor.

Table S13. Secondary outcomes: General functioning (WHODAS 12-item total score). Bayesian results of between groups comparisons.

Timepoint	Ref. Group	Ref. N	Comp. Group	Comp. N	Ref. Mean (SD)	Comp. Mean (SD)	Comp. vs Ref. Estimate	Lower 95% CrI	Upper 95% CrI	BF (ICTI < Comp)	BF (ICTI > Comp)
Baseline	ICTI	25	AC	30	22.670 (18.412)	19.725 (13.059)					
Baseline	ICTI	25	TAU	19	22.670 (18.412)	15.135 (10.753)					
Week 4	ICTI	24	AC	26	15.020 (14.551)	18.272 (15.061)	6.261	1.278	11.276	138.0887	0.0072
Week 4	ICTI	24	TAU	17	15.020 (14.551)	16.424 (10.620)	8.241	2.630	13.837	507.7719	0.0020
Week 12	ICTI	21	AC	24	15.974 (17.095)	13.285 (11.424)	1.736	-3.682	7.109	2.8113	0.3557
Week 12	ICTI	21	TAU	19	15.974 (17.095)	18.094 (16.739)	8.489	2.587	14.343	401.7778	0.0025
Week 24	ICTI	25	AC	24	13.252 (14.369)	14.499 (16.570)	4.547	-0.699	9.792	21.0490	0.0475
Week 24	ICTI	25	TAU	16	13.252 (14.369)	18.363 (15.631)	10.064	4.148	15.972	2229.7692	0.0004

Note. Secondary Bayesian analysis of general functioning measured using the WHODAS 2.0 12-item total score at weeks 4, 12 and 24 was modelled with the use of an identity link function and a normal distribution. The scale is scored such that lower total scores are indicative of greater general functioning.

Bayes factors (BF) quantifying the evidence for an alternative hypothesis (H_1) over the null hypothesis (H_0) are presented to illustrate evidence in favour of positive and negative treatment effects of the ICTI for WHODAS 2.0 total scores at all follow-up time-points (weeks 4, 12 and 24). The ICTI arm is treated as the reference group with the AC and TAU arms treated as the comparators. The estimate shows the expected difference between the groups in the model. Positive estimates > 0 show model estimated scores of general functioning impairment were greater in the comparator arms relative to the ICTI. Means (SD) are presented at each time-point for the analysis population included in the model.

Abbreviations: PHQ-2 = Patient Health Questionnaire (2-item version); ICTI = Imagery-Competing Task Intervention; AC = active control; TAU = treatment-as-usual; Ref. = reference [group]; Comp. = comparator [group]; SD = standard deviation; CrI = credible interval; BF = Bayes factor.

Table S14. Secondary outcomes: Quality of Life (EQ-5D-5L). Bayesian results of between groups comparisons.

We present the results secondary analyses for each of the 5 categorical items in addition (scored from 1 = no problems, to 5 = extreme problems), and a visual analogue scale (VAS) item wherein participants self-assess their overall health on a scale ranging from 100 ('The best health you can imagine') to 0 ('The worst health you can imagine').

Secondary Bayesian analysis of EQ-5D-5L ordinal items at weeks 4, 12 and 24 were modelled with a logit link function in a cumulative regression model. Analysis of the EQ-5D-5L VAS score at weeks 4, 12 and 24 were modelled with linear regression with the use of an identity link function and a normal distribution.

Table S14A. Secondary outcomes: Quality of Life (EQ-5D-5L), Mobility item.

Timepoint	Ref. Group	Ref. N	Comp. Group	Comp. N	Ref. Mean (SD)	Comp. Mean (SD)	Comp. vs Ref. Estimate	Lower 95% CrI	Upper 95% CrI	BF (ICTI < Comp)	BF (ICTI > Comp)
Baseline	ICTI	25	AC	30	1.400 (0.764)	1.433 (0.679)					
Baseline	ICTI	25	TAU	19	1.400 (0.764)	1.053 (0.229)					
Week 4	ICTI	24	AC	26	1.375 (0.711)	1.231 (0.514)	-1.223	-5.853	3.131	0.4086	2.4477
Week 4	ICTI	24	TAU	17	1.375 (0.711)	1.118 (0.332)	0.337	-5.097	5.662	1.2501	0.7999
Week 12	ICTI	21	AC	24	1.381 (0.865)	1.125 (0.338)	-3.031	-8.166	1.669	0.1127	8.8757
Week 12	ICTI	21	TAU	19	1.381 (0.865)	1.211 (0.535)	0.478	-4.928	5.721	1.3820	0.7236
Week 24	ICTI	25	AC	24	1.440 (0.712)	1.208 (0.415)	-2.218	-6.883	2.152	0.1836	5.4462
Week 24	ICTI	25	TAU	16	1.440 (0.712)	1.125 (0.342)	0.649	-4.593	6.024	1.4832	0.6742

Table S14B. Secondary outcomes: Quality of Life (EQ-5D-5L), Self-care item.

Timepoint	Ref. Group	Ref. N	Comp. Group	Comp. N	Ref. Mean (SD)	Comp. Mean (SD)	Comp. vs Ref. Estimate	Lower 95% CrI	Upper 95% CrI	BF (ICTI < Comp)	BF (ICTI > Comp)
Baseline	ICTI	25	AC	30	1.480 (0.872)	1.100 (0.305)					
Baseline	ICTI	25	TAU	19	1.480 (0.872)	1.158 (0.501)					
Week 4	ICTI	24	AC	26	1.208 (0.509)	1.192 (0.402)	3.279	-0.101	7.154	33.9398	0.0295
Week 4	ICTI	24	TAU	17	1.208 (0.509)	1.176 (0.529)	1.370	-2.746	5.453	3.1233	0.3202
Week 12	ICTI	21	AC	24	1.286 (0.717)	1.000 (0.000)	-5.725	-19.163	2.641	0.1530	6.5379
Week 12	ICTI	21	TAU	19	1.286 (0.717)	1.211 (0.713)	1.315	-2.904	5.489	2.9389	0.3403
Week 24	ICTI	25	AC	24	1.160 (0.374)	1.083 (0.282)	1.956	-2.097	6.102	5.0679	0.1973
Week 24	ICTI	25	TAU	16	1.160 (0.374)	1.188 (0.544)	1.422	-3.008	5.638	3.1017	0.3224

Table S14C. Secondary outcomes: Quality of Life (EQ-5D-5L), Usual activities item.

Timepoint	Ref. Group	Ref. N	Comp. Group	Comp. N	Ref. Mean (SD)	Comp. Mean (SD)	Comp. vs Ref. Estimate	Lower 95% CrI	Upper 95% CrI	BF (ICTI < Comp)	BF (ICTI > Comp)
Baseline	ICTI	25	AC	30	1.840 (1.106)	1.700 (0.837)					
Baseline	ICTI	25	TAU	19	1.840 (1.106)	1.316 (0.478)					
Week 4	ICTI	24	AC	26	1.583 (0.881)	1.500 (0.990)	-1.077	-3.842	1.609	0.2731	3.6618
Week 4	ICTI	24	TAU	17	1.583 (0.881)	1.412 (0.507)	2.066	-0.728	5.048	12.6599	0.0790
Week 12	ICTI	21	AC	24	1.524 (0.750)	1.250 (0.532)	-1.174	-4.140	1.689	0.2667	3.7492
Week 12	ICTI	21	TAU	19	1.524 (0.750)	1.263 (0.562)	0.693	-2.376	3.790	2.0671	0.4838
Week 24	ICTI	25	AC	24	1.520 (0.770)	1.458 (0.779)	0.167	-2.549	2.841	1.2318	0.8118
Week 24	ICTI	25	TAU	16	1.520 (0.770)	1.500 (0.730)	2.789	-0.098	5.923	33.2992	0.0300

Table S14D. Secondary outcomes: Quality of Life (EQ-5D-5L), Pain-discomfort item.

Timepoint	Ref. Group	Ref. N	Comp. Group	Comp. N	Ref. Mean (SD)	Comp. Mean (SD)	Comp. vs Ref. Estimate	Lower 95% CrI	Upper 95% CrI	BF (ICTI < Comp)	BF (ICTI > Comp)
Baseline	ICTI	25	AC	30	2.200 (1.323)	2.067 (1.172)					
Baseline	ICTI	25	TAU	19	2.200 (1.323)	1.737 (0.733)					
Week 4	ICTI	24	AC	26	2.125 (1.262)	1.962 (0.999)	0.046	-2.388	2.483	1.0554	0.9476
Week 4	ICTI	24	TAU	17	2.125 (1.262)	1.765 (0.752)	0.285	-2.497	3.047	1.4053	0.7116
Week 12	ICTI	21	AC	24	2.000 (1.049)	1.917 (0.830)	0.990	-1.543	3.593	3.5814	0.2792
Week 12	ICTI	21	TAU	19	2.000 (1.049)	1.632 (0.684)	0.281	-2.597	3.144	1.3816	0.7238
Week 24	ICTI	25	AC	24	2.040 (1.172)	1.875 (0.900)	-0.665	-3.175	1.794	0.4227	2.3658
Week 24	ICTI	25	TAU	16	2.040 (1.172)	1.625 (0.806)	0.285	-2.574	3.106	1.3965	0.7161

Table S14E. Secondary Outcomes: Quality of Life (EQ-5D-5L), Anxiety-Depression Item.

Timepoint	Ref. Group	Ref. N	Comp. Group	Comp. N	Ref. Mean (SD)	Comp. Mean (SD)	Comp. vs Ref. Estimate	Lower 95% CrI	Upper 95% CrI	BF (ICTI < Comp)	BF (ICTI > Comp)
Baseline	ICTI	25	AC	30	2.360 (0.952)	2.200 (0.925)					
Baseline	ICTI	25	TAU	19	2.360 (0.952)	2.421 (0.961)					
Week 4	ICTI	24	AC	26	1.833 (0.702)	2.115 (0.993)	1.784	0.073	3.587	48.5092	0.0206
Week 4	ICTI	24	TAU	17	1.833 (0.702)	2.471 (0.800)	2.793	0.909	4.789	579.0000	0.0017
Week 12	ICTI	21	AC	24	1.905 (0.700)	1.958 (0.751)	0.476	-1.303	2.247	2.3640	0.4230
Week 12	ICTI	21	TAU	19	1.905 (0.700)	2.211 (0.855)	1.107	-0.799	3.044	6.8815	0.1453
Week 24	ICTI	25	AC	24	1.840 (0.800)	1.833 (0.868)	0.612	-1.153	2.385	3.0696	0.3258
Week 24	ICTI	25	TAU	16	1.840 (0.800)	2.312 (0.946)	1.907	-0.018	3.905	37.3598	0.0268

Table S14F. Secondary outcomes: Quality of Life (EQ-5D-5L), Overall health (visual analogue scale) item.

Timepoint	Ref. Group	Ref. N	Comp. Group	Comp. N	Ref. Mean (SD)	Comp. Mean (SD)	Comp. vs Ref. Estimate	Lower 95% CrI	Upper 95% CrI	BF (ICTI < Comp)	BF (ICTI > Comp)
Baseline	ICTI	25	AC	30	67.280 (17.028)	71.700 (16.291)					
Baseline	ICTI	25	TAU	19	67.280 (17.028)	72.368 (12.575)					
Week 4	ICTI	24	AC	26	73.042 (18.179)	70.077 (20.096)	-6.249	-12.837	0.357	0.0326	30.6680
Week 4	ICTI	24	TAU	16	73.042 (18.179)	72.938 (15.541)	-2.157	-9.438	5.143	0.3884	2.5748
Week 12	ICTI	21	AC	24	72.952 (17.054)	75.167 (19.759)	-2.868	-10.440	4.667	0.2929	3.4137
Week 12	ICTI	21	TAU	19	72.952 (17.054)	75.211 (17.492)	-2.410	-10.448	5.585	0.3830	2.6108
Week 24	ICTI	25	AC	23	73.840 (18.674)	74.696 (20.488)	-2.632	-9.953	4.663	0.3150	3.1742
Week 24	ICTI	25	TAU	16	73.840 (18.674)	71.625 (17.633)	-4.971	-13.026	3.143	0.1289	7.7587

Note. Bayes factors (BF) quantifying the evidence for an alternative hypothesis (H_1) over the null hypothesis (H_0) are presented to illustrate evidence in favour of positive and negative treatment effects of the ICTI for EQ-5D-5L items at all follow-up time-points (weeks 4, 12 and 24). The ICTI arm is treated as the reference group with the AC and TAU arms treated as the comparators. The estimate shows the expected difference between the groups in the model. For the five ordinal items, positive estimates > 0 show model estimated scores of were greater in the comparator arms relative to the ICTI. For the VAS item, negative estimates < 0 show model estimated scores of were lower in the comparator arms relative to the ICTI. Means (SD) are presented at each time-point for the analysis population included in the model.

Abbreviations: EQ-5D-5L = European Quality of Life Five Dimension Five Level Scale; VAS = Visual Analogue Scale; ICTI = Imagery-Competing Task Intervention; AC = active control; TAU = treatment-as-usual; Ref. = reference [group]; Comp. = comparator [group]; SD = standard deviation; CrI = credible interval; BF = Bayes factor.

Table S15. Secondary outcomes: Occupational outcomes, work engagement (SWEBO Engagement Subscale). Bayesian results of between groups comparisons

The SWEBO consisted of 19-items assessing either work engagement or work burnout. The measure was analysed as two distinct scales, work engagement and work burnout, both calculated as a mean of relevant items. For the SWEBO work engagement and work burnout scales, higher scores are indicative of greater engagement in one's work and a greater degree of burnout, respectively.

Table S15A. Secondary outcomes: Occupational outcomes, SWEBO Work Engagement subscale.

Timepoint	Ref. Group	Ref. N	Comp. Group	Comp. N	Ref. Mean (SD)	Comp. Mean (SD)	Comp. vs Ref. Estimate	Lower 95% CrI	Upper 95% CrI	BF (ICTI < Comp)	BF (ICTI > Comp)
Baseline	ICTI	25	AC	30	2.097 (0.428)	2.193 (0.601)					
Baseline	ICTI	25	TAU	19	2.097 (0.428)	2.242 (0.635)					
Week 4	ICTI	24	AC	26	2.455 (0.505)	2.297 (0.619)	-0.261	-0.542	0.019	0.0352	28.4043
Week 4	ICTI	24	TAU	17	2.455 (0.505)	2.165 (0.464)	-0.397	-0.711	-0.085	0.0070	143.2786
Week 12	ICTI	21	AC	24	2.353 (0.467)	2.542 (0.662)	0.062	-0.231	0.356	1.9472	0.5136
Week 12	ICTI	21	TAU	19	2.353 (0.467)	2.411 (0.615)	-0.058	-0.370	0.257	0.5569	1.7956
Week 24	ICTI	25	AC	24	2.341 (0.368)	2.571 (0.832)	0.118	-0.164	0.401	3.8705	0.2584
Week 24	ICTI	25	TAU	17	2.341 (0.368)	2.242 (0.758)	-0.146	-0.458	0.166	0.2175	4.5982

Note. The SWEBO work engagement and SWEBO work burnout subscales were modelled with the use of an identity link function and a normal distribution.

Bayes Factors (BF) quantifying the evidence for an alternative hypothesis (H_1) over the null hypothesis (H_0) are presented to illustrate evidence in favour of positive and negative treatment effects of the ICTI at all follow-up time-points (weeks 4, 12 and 24). The ICTI arm is treated as the reference group with the AC and TAU arms treated as the comparators. The Estimate shows the expected difference between the groups in the model. For the Work Engagement scale, negative estimates < 0 show model estimated work engagement scores were lower in the comparator arms relative to the ICTI. For the work burnout scale, positive estimates > 0 show model estimated work burnout scores of were greater in the comparator arms relative to the ICTI. Means (SD) are presented at each time-point for the analysis population included in the model.

Abbreviations: SWEBO = Scale of Work Engagement and Burnout; ICTI = Imagery-Competing Task Intervention; AC = active control; TAU = treatment-as-usual; Ref. = reference [group]; Comp. = comparator [group]; SD = standard deviation; CrI = credible interval; BF = Bayes factor.

Table S15B. Secondary outcomes: Occupational outcomes, SWEBO work burnout subscale.

Timepoint	Ref. Group	Ref. N	Comp. Group	Comp. N	Ref. Mean (SD)	Comp. Mean (SD)	Comp. vs Ref. Estimate	Lower 95% CrI	Upper 95% CrI	BF (ICTI < Comp)	BF (ICTI > Comp)
Baseline	ICTI	25	AC	30	2.065 (0.548)	2.142 (0.662)					
Baseline	ICTI	25	TAU	19	2.065 (0.548)	2.133 (0.551)					
Week 4	ICTI	24	AC	26	1.689 (0.329)	2.052 (0.628)	0.334	0.063	0.604	124.8134	0.0080
Week 4	ICTI	24	TAU	17	1.689 (0.329)	2.207 (0.547)	0.502	0.198	0.804	1363.7059	0.0007
Week 12	ICTI	21	AC	24	1.942 (0.451)	1.885 (0.713)	-0.064	-0.344	0.217	0.4793	2.0863
Week 12	ICTI	21	TAU	19	1.942 (0.451)	1.969 (0.604)	0.023	-0.280	0.326	1.2766	0.7834
Week 24	ICTI	25	AC	24	1.870 (0.413)	1.926 (0.792)	0.088	-0.183	0.361	2.8109	0.3558
Week 24	ICTI	25	TAU	17	1.870 (0.413)	2.226 (0.696)	0.271	-0.030	0.573	24.8122	0.0403

Note. The SWEBO work engagement and SWEBO work burnout subscales were modelled with the use of an identity link function and a normal distribution.

Bayes Factors (BF) quantifying the evidence for an alternative hypothesis (H_1) over the null hypothesis (H_0) are presented to illustrate evidence in favour of positive and negative treatment effects of the ICTI at all follow-up time-points (weeks 4, 12 and 24). The ICTI arm is treated as the reference group with the AC and TAU arms treated as the comparators. The Estimate shows the expected difference between the groups in the model. For the Work Engagement scale, negative estimates < 0 show model estimated work engagement scores were lower in the comparator arms relative to the ICTI. For the work burnout scale, positive estimates > 0 show model estimated work burnout scores of were greater in the comparator arms relative to the ICTI. Means (SD) are presented at each time-point for the analysis population included in the model.

Abbreviations: SWEBO = Scale of Work Engagement and Burnout; ICTI = Imagery-Competing Task Intervention; AC = active control; TAU = treatment-as-usual; Ref. = reference [group]; Comp. = comparator [group]; SD = standard deviation; CrI = credible interval; BF = Bayes factor.

Table S16. Secondary outcomes: Occupational outcomes, Intention to leave job (ITL). Bayesian results of between groups comparisons.

Timepoint	Ref. Group	Ref. N	Comp. Group	Comp. N	Ref. Mean (SD)	Comp. Mean (SD)	Comp. vs Ref. Estimate	Lower 95% CrI	Upper 95% CrI	BF (ICTI < Comp)	BF (ICTI > Comp)
Baseline	ICTI	25	AC	30	10.080 (3.439)	9.800 (4.114)					
Baseline	ICTI	25	TAU	19	10.080 (3.439)	10.632 (3.919)					
Week 4	ICTI	24	AC	26	10.583 (4.106)	8.577 (4.989)	-1.616	-3.666	0.445	0.0663	15.0821
Week 4	ICTI	24	TAU	17	10.583 (4.106)	9.706 (4.511)	-1.600	-3.915	0.713	0.0953	10.4943
Week 12	ICTI	21	AC	24	10.667 (4.127)	10.042 (4.947)	-0.616	-2.767	1.525	0.4009	2.4943
Week 12	ICTI	21	TAU	19	10.667 (4.127)	11.316 (3.987)	0.205	-2.091	2.521	1.3179	0.7588
Week 24	ICTI	25	AC	24	10.480 (3.874)	9.625 (4.942)	-0.627	-2.719	1.445	0.3799	2.6326
Week 24	ICTI	25	TAU	17	10.480 (3.874)	10.294 (4.727)	-0.605	-2.907	1.711	0.4290	2.3312

Note. Secondary Bayesian analysis of intention to leave one's job, assessed using the three-item ITL questionnaire at weeks 4, 12 and 24, was modelled with the use of an identity link function and a normal distribution. The scale is scored such that lower scores are indicative of greater desire to leave one's job.

Bayes factors (BF) quantifying the evidence for an alternative hypothesis (H_1) over the null hypothesis (H_0) are presented to illustrate evidence in favour of positive and negative treatment effects of the ICTI at all follow-up time-points (weeks 4, 12 and 24). The ICTI arm is treated as the reference group with the AC and TAU arms treated as the comparators. The estimate shows the expected difference between the groups in the model. Negative estimates < 0 show model estimated scores of were lower in the comparator arms relative to the ICTI. Means (SD) are presented at each time-point for the analysis population included in the model.

Abbreviations: ICTI = Imagery-Competing Task Intervention; AC = active control; TAU = treatment-as-usual; Ref. = reference [group]; Comp. = comparator [group]; SD = standard deviation; CrI = credible interval; BF = Bayes factor.

Table S17. Secondary outcomes: Occupational outcomes, Sickness absence. Bayesian results of between groups comparisons

Timepoint	Ref. Group	Ref. N	Comp. Group	Comp. N	Ref. Mean (SD)	Comp. Mean (SD)	Comp. vs Ref. Estimate	Lower 95% CrI	Upper 95% CrI	BF (ICTI < Comp)	BF (ICTI > Comp)
Baseline	ICTI	25	AC	30	0.920 (2.581)	2.000 (5.681)					
Baseline	ICTI	25	TAU	19	0.920 (2.581)	1.737 (4.039)					
Week 4	ICTI	24	AC	26	3.167 (6.479)	1.308 (3.069)	-1.063	-2.901	0.747	0.1367	7.3142
Week 4	ICTI	24	TAU	17	3.167 (6.479)	0.059 (0.243)	-4.557	-7.991	-1.685	0.0009	1053.5455
Week 12	ICTI	21	AC	24	2.190 (6.104)	1.208 (2.553)	-0.593	-2.599	1.402	0.3788	2.6401
Week 12	ICTI	21	TAU	19	2.190 (6.104)	1.316 (3.465)	-0.858	-3.133	1.348	0.2813	3.5553
Week 24	ICTI	25	AC	24	0.160 (0.473)	2.333 (6.825)	2.492	0.283	4.763	71.5453	0.0140
Week 24	ICTI	25	TAU	17	0.160 (0.473)	0.588 (1.502)	1.539	-0.867	4.074	8.5458	0.1170

Note. A single-item measure assessed the number of days of sickness absence over the past 4-weeks(12). This item was modelled with a log link function in a negative binomial regression model.

Bayes factors (BF) quantifying the evidence for an alternative hypothesis (H_1) over the null hypothesis (H_0) are presented to illustrate evidence in favour of positive and negative treatment effects of the ICTI at all follow-up time-points (weeks 4, 12 and 24). The ICTI arm is treated as the reference group with the AC and TAU arms treated as the comparators. The estimate shows the expected difference between the logs of the groups. Positive estimates > 0 show model estimated days of sickness absence were greater in the comparator arms relative to the ICTI. Means (SD) are presented at each time-point for the analysis population included in the model.

Abbreviations: ICTI = Imagery-Competing Task Intervention; AC = active control; TAU = treatment-as-usual; Ref. = reference [group]; Comp. = comparator [group]; SD = standard deviation; CrI = credible interval; BF = Bayes factor.

Table S18. Secondary outcomes: Intrusive Memory Ratings (IMR). Bayesian results of between groups comparisons

The impact of IMs was assessed using for 7 items scored on an 11-point scale from 0 ("not at all") to 10 ("extremely") with higher scores indicating a greater degree of impact. Secondary Bayesian analysis of IMR items at weeks 4, 12 and 24 were modelled with a logit link function in a cumulative regression model.

Table S18A. IMR Q1. Approximately how often did IMs of the traumatic event pop into your mind?

Timepoint	Ref. Group	Ref. N	Comp. Group	Comp. N	Ref. Mean (SD)	Comp. Mean (SD)	Comp. vs Ref. Estimate	Lower 95% CrI	Upper 95% CrI	BF (ICTI < Comp)	BF (ICTI > Comp)
Baseline	ICTI	26	AC	30	4.231 (0.992)	4.100 (0.995)					
Baseline	ICTI	26	TAU	20	4.231 (0.992)	3.800 (1.361)					
Week 4	ICTI	25	AC	29	3.280 (1.646)	4.414 (1.402)	2.639	0.987	4.391	1083.1121	0.0009
Week 4	ICTI	25	TAU	18	3.280 (1.646)	4.333 (1.372)	3.138	1.153	5.245	1044.0450	0.0010
Week 12	ICTI	21	AC	24	2.095 (1.338)	3.792 (1.865)	4.331	2.459	6.325	Inf	<0.0001
Week 12	ICTI	21	TAU	19	2.095 (1.338)	3.000 (1.700)	2.669	0.593	4.831	161.6928	0.0062
Week 24	ICTI	26	AC	24	1.769 (0.992)	3.500 (1.285)	4.540	2.725	6.511	Inf	<0.0001
Week 24	ICTI	26	TAU	18	1.769 (0.992)	3.278 (1.776)	4.207	2.098	6.455	16570.4286	<0.0001

Table S18B. IMR Q2. How distressing were your IMs?

Timepoint	Ref. Group	Ref. N	Comp. Group	Comp. N	Ref. Mean (SD)	Comp. Mean (SD)	Comp. vs Ref. Estimate	Lower 95% CrI	Upper 95% CrI	BF (ICTI < Comp)	BF (ICTI > Comp)
Baseline	ICTI	26	AC	30	5.885 (2.286)	5.567 (1.478)					
Baseline	ICTI	26	TAU	20	5.885 (2.286)	5.250 (1.618)					
Week 4	ICTI	25	AC	29	5.160 (2.609)	5.828 (1.983)	1.142	-0.399	2.727	12.7816	0.0782
Week 4	ICTI	25	TAU	18	5.160 (2.609)	6.167 (1.790)	1.266	-0.462	3.039	12.4073	0.0806
Week 12	ICTI	21	AC	24	3.667 (3.055)	5.458 (2.813)	2.663	0.941	4.457	1034.7143	0.0010
Week 12	ICTI	21	TAU	19	3.667 (3.055)	5.368 (3.004)	2.367	0.517	4.263	148.4845	0.0067
Week 24	ICTI	26	AC	24	2.962 (2.506)	5.292 (2.255)	2.987	1.345	4.701	4639.0000	0.0002
Week 24	ICTI	26	TAU	18	2.962 (2.506)	5.167 (3.111)	2.729	0.896	4.634	533.5622	0.0019

Table S18C. IMR Q3. How much did they disrupt your concentration?

Timepoint	Ref. Group	Ref. N	Comp. Group	Comp. N	Ref. Mean (SD)	Comp. Mean (SD)	Comp. vs Ref. Estimate	Lower 95% CrI	Upper 95% CrI	BF (ICTI < Comp)	BF (ICTI > Comp)
Baseline	ICTI	26	AC	30	7.423 (2.419)	7.233 (2.515)					
Baseline	ICTI	26	TAU	20	7.423 (2.419)	6.800 (2.419)					
Week 4	ICTI	25	AC	29	5.360 (2.596)	6.172 (2.450)	1.588	-0.130	3.357	27.8917	0.0359
Week 4	ICTI	25	TAU	18	5.360 (2.596)	6.278 (2.137)	1.849	-0.117	3.889	29.7611	0.0336
Week 12	ICTI	21	AC	24	3.429 (2.976)	5.500 (3.022)	2.878	0.991	4.829	767.2119	0.0013
Week 12	ICTI	21	TAU	19	3.429 (2.976)	5.684 (3.233)	3.372	1.315	5.512	1545.6667	0.0006
Week 24	ICTI	26	AC	24	2.885 (2.658)	5.042 (2.386)	3.114	1.303	5.011	2415.6667	0.0004
Week 24	ICTI	26	TAU	18	2.885 (2.658)	5.056 (3.572)	3.494	1.427	5.637	1811.5000	0.0006

Table S18D. IMR Q4. How much did they interfere with what you were doing?

Timepoint	Ref. Group	Ref. N	Comp. Group	Comp. N	Ref. Mean (SD)	Comp. Mean (SD)	Comp. vs Ref. Estimate	Lower 95% CrI	Upper 95% CrI	BF (ICTI < Comp)	BF (ICTI > Comp)
Baseline	ICTI	26	AC	30	7.154 (2.477)	6.267 (2.212)					
Baseline	ICTI	26	TAU	20	7.154 (2.477)	5.650 (2.231)					
Week 4	ICTI	25	AC	29	4.840 (2.561)	5.586 (2.442)	1.745	0.074	3.476	47.9865	0.0208
Week 4	ICTI	25	TAU	18	4.840 (2.561)	5.444 (1.886)	1.393	-0.472	3.315	12.6889	0.0788
Week 12	ICTI	21	AC	24	3.048 (2.819)	4.667 (2.929)	2.908	1.046	4.823	934.4839	0.0011
Week 12	ICTI	21	TAU	19	3.048 (2.819)	5.053 (3.100)	3.435	1.425	5.532	1999.0000	0.0005
Week 24	ICTI	26	AC	24	2.538 (2.249)	4.625 (2.551)	3.411	1.599	5.316	6104.2632	0.0002
Week 24	ICTI	26	TAU	18	2.538 (2.249)	4.222 (3.318)	3.168	1.139	5.292	1007.6957	0.0010

Table S18E. IMR Q4a. And for how long each time (approximately)?

Timepoint	Ref. Group	Ref. N	Comp. Group	Comp. N	Ref. Mean (SD)	Comp. Mean (SD)	Comp. vs Ref. Estimate	Lower 95% CrI	Upper 95% CrI	BF (ICTI < Comp)	BF (ICTI > Comp)
Baseline	ICTI	26	AC	30	1.577 (0.703)	1.867 (0.571)					
Baseline	ICTI	26	TAU	20	1.577 (0.703)	1.500 (0.607)					
Week 4	ICTI	25	AC	29	1.400 (0.577)	1.897 (0.860)	1.809	0.059	3.691	46.1545	0.0217
Week 4	ICTI	25	TAU	18	1.400 (0.577)	1.444 (0.511)	0.503	-1.466	2.500	2.2584	0.4428
Week 12	ICTI	21	AC	24	1.238 (0.625)	1.542 (0.658)	1.344	-0.817	3.662	7.8712	0.1270
Week 12	ICTI	21	TAU	19	1.238 (0.625)	1.474 (0.697)	1.583	-0.680	3.992	10.5503	0.0948
Week 24	ICTI	26	AC	24	1.269 (0.604)	1.750 (0.737)	2.411	0.497	4.495	150.2386	0.0067
Week 24	ICTI	26	TAU	18	1.269 (0.604)	1.444 (0.616)	1.404	-0.693	3.617	9.3655	0.1068

Table S18F. IMR Q5. How much did your IMs affect your work functioning?

Timepoint	Ref. Group	Ref. N	Comp. Group	Comp. N	Ref. Mean (SD)	Comp. Mean (SD)	Comp. vs Ref. Estimate	Lower 95% CrI	Upper 95% CrI	BF (ICTI < Comp)	BF (ICTI > Comp)
Baseline	ICTI	26	AC	30	5.615 (2.684)	5.533 (2.460)					
Baseline	ICTI	26	TAU	20	5.615 (2.684)	4.700 (2.618)					
Week 4	ICTI	25	AC	29	3.800 (2.814)	4.552 (2.746)	0.010	-1.693	1.746	1.0081	0.9919
Week 4	ICTI	25	TAU	18	3.800 (2.814)	4.722 (2.585)	0.733	-0.996	2.514	3.9086	0.2558
Week 12	ICTI	21	AC	24	2.381 (2.500)	3.958 (3.141)	1.230	-0.679	3.214	8.6450	0.1157
Week 12	ICTI	21	TAU	19	2.381 (2.500)	4.158 (3.731)	1.737	-0.191	3.767	24.5845	0.0407
Week 24	ICTI	26	AC	24	1.962 (1.777)	3.750 (3.011)	1.980	0.101	3.955	50.1915	0.0199
Week 24	ICTI	26	TAU	18	1.962 (1.777)	3.667 (3.325)	1.850	-0.056	3.855	33.4828	0.0299

Table S18G. IMR Q7. How much did your IMs affect your functioning in other areas of your life (e.g. relationships with other people, parenting, social life, study, housework, voluntary work etc.)

Timepoint	Ref. Group	Ref. N	Comp. Group	Comp. N	Ref. Mean (SD)	Comp. Mean (SD)	Comp. vs Ref. Estimate	Lower 95% CrI	Upper 95% CrI	BF (ICTI < Comp)	BF (ICTI > Comp)
Baseline	ICTI	26	AC	30	6.038 (2.522)	5.567 (2.635)					
Baseline	ICTI	26	TAU	20	6.038 (2.522)	5.800 (2.567)					
Week 4	ICTI	25	AC	29	3.960 (2.731)	5.207 (2.993)	1.656	0.102	3.280	54.1331	0.0185
Week 4	ICTI	25	TAU	18	3.960 (2.731)	5.167 (2.333)	1.351	-0.395	3.129	14.5101	0.0689
Week 12	ICTI	21	AC	24	2.476 (2.442)	3.958 (3.029)	2.722	0.862	4.667	474.4098	0.0021
Week 12	ICTI	21	TAU	19	2.476 (2.442)	4.789 (3.660)	3.174	1.184	5.250	1246.3118	0.0008
Week 24	ICTI	26	AC	24	1.962 (1.587)	3.500 (2.670)	3.083	1.319	4.970	3313.2857	0.0003
Week 24	ICTI	26	TAU	18	1.962 (1.587)	3.222 (2.819)	2.354	0.437	4.341	125.3617	0.0080

Note. Bayes factors (BF) quantifying the evidence for an alternative hypothesis (H_1) over the null hypothesis (H_0) are presented to illustrate evidence in favour of positive and negative treatment effects of the ICTI at all follow-up time-points (weeks 4, 12 and 24). The ICTI arm is treated as the reference group with the AC and TAU arms treated as the comparators. The estimate shows the expected difference between the groups in the model. Positive estimates > 0 show model estimated scores of were greater in the comparator arms relative to the ICTI. Means (SD) are presented at each time-point for the analysis population included in the model.

Abbreviations: IMR = Intrusive Memory Rating Scale; ICTI = Imagery-Competing Task Intervention; AC = active control; TAU = treatment-as-usual; Ref. = reference [group]; Comp. = comparator [group]; SD = standard deviation; CrI = credible interval; BF = Bayes factor.

Table S19. Other outcomes. Intervention feedback questionnaire (IFQ). Descriptive summary statistics.

		ARM	
**Variable**	**All**	**AC**	**ICTI**
N	47	22	25
How easy did you find it to use the brief cognitive task?			
Mean (SD)	8.57 (2.29)	8.36 (2.90)	8.76 (1.61)
Median (IQR)	10.00 (8.00 - 10.00)	10.00 (8.25 - 10.00)	9.00 (8.00 - 10.00)
Min - Max	0 - 10	0 - 10	4 - 10
How helpful did you find the brief cognitive task?			
Mean (SD)	6.64 (2.82)	5.05 (2.98)	8.04 (1.77)
Median (IQR)	7.00 (5.00 - 9.00)	5.00 (2.25 - 7.00)	8.00 (7.00 - 10.00)
Min - Max	0 - 10	0 - 10	5 - 10
How burdensome did you find the brief cognitive task?			
Mean (SD)	6.32 (2.51)	6.05 (3.11)	6.56 (1.87)
Median (IQR)	7.00 (5.00 - 8.00)	7.00 (5.00 - 8.00)	7.00 (6.00 - 7.00)
Min - Max	0 - 10	0 - 10	2 - 10
How distressing did you find the brief cognitive task?			
Mean (SD)	8.77 (1.67)	9.36 (1.18)	8.24 (1.88)
Median (IQR)	10.00 (8.00 - 10.00)	10.00 (9.25 - 10.00)	8.00 (8.00 - 10.00)
Min - Max	2 - 10	6 - 10	2 - 10
Overall, how acceptable did you find the brief cognitive task?			
Mean (SD)	7.26 (2.62)	6.50 (2.79)	7.92 (2.33)
Median (IQR)	8.00 (5.00 - 9.50)	6.50 (5.00 - 9.00)	8.00 (7.00 - 10.00)
Min - Max	0 - 10	0 - 10	0 - 10
If you were having intrusive memories in the future, how willing would you be to use the brief cognitive task if it was offered to you as something that would help?			
Mean (SD)	7.04 (3.06)	5.86 (3.66)	8.08 (1.98)
Median (IQR)	8.00 (5.00 - 10.00)	7.50 (2.75 - 8.75)	8.00 (7.00 - 10.00)
Min - Max	0 - 10	0 - 10	5 - 10
If a colleague or friend was having intrusive memories, how confident would you be in recommending the brief cognitive task to them?			
Mean (SD)	6.57 (3.13)	5.32 (3.58)	7.68 (2.21)
Median (IQR)	7.00 (5.00 - 9.50)	6.00 (2.25 - 8.00)	8.00 (7.00 - 10.00)
Min - Max	0 - 10	0 - 10	2 - 10
How much do you feel that this brief cognitive task could be used within NHS Trusts/healthcare organisations to support staff who have experienced work-related traumatic events?			
Mean (SD)	6.85 (2.96)	5.55 (3.31)	8.00 (2.06)
Median (IQR)	7.00 (5.00 - 10.00)	5.50 (3.00 - 8.00)	8.00 (7.00 - 10.00)
Min - Max	0 - 10	0 - 10	3 - 10
Total score			
Mean (SD)	58.02 (16.48)	52.05 (19.17)	63.28 (11.71)
Median (IQR)	62.00 (46.50 - 70.00)	52.00 (42.25 - 67.75)	67.00 (59.00 - 72.00)
Min - Max	10 - 80	10 - 80	41 - 78

Note. The Feedback Questionnaire was completed four-weeks after receiving access to the brief online intervention task (ICTI or AC). Each quantitative item was assessed on an 11-point scale (range 0-10) with higher scores indicating more favourable opinions towards the respective task (e.g., "How easy did you find it to use the

brief cognitive task?”, 0 = *Not at all easy* and 10 = *very easy*; “How burdensome did you find the brief cognitive task?”, 0 = *Very burdensome* and 10 = *Not at all burdensome*). The total score is calculated as the sum of the eight quantitative items.

Abbreviations. SD = standard deviation; IQR = interquartile range; ICTI = Imagery-Competing Task Intervention; AC = active control.

Table S20: Other outcome measures. Feedback questionnaire. Bayesian results of between groups comparisons.

The eight quantitative items of the IFQ were modelled using a cumulative regression model with logit link function with outcomes modelled using a single fixed effect of 'arm'.

Table S20A. How easy did you find it to use the brief cognitive task?

Timepoint	Ref. Group	Ref. N	Comp. Group	Comp. N	Ref. Mean (SD)	Comp. Mean (SD)	Comp. vs Ref. Estimate	Lower 95% CrI	Upper 95% CrI	BF (ICTI < Comp)	BF (ICTI > Comp)
Baseline	ICTI	25	AC	22	8.760 (1.615)	8.364 (2.904)	0.327	-0.777	1.452	2.5562	0.3912

Table S20B. How helpful did you find the brief cognitive task?

Timepoint	Ref. Group	Ref. N	Comp. Group	Comp. N	Ref. Mean (SD)	Comp. Mean (SD)	Comp. vs Ref. Estimate	Lower 95% CrI	Upper 95% CrI	BF (ICTI < Comp)	BF (ICTI > Comp)
Baseline	ICTI	25	AC	22	8.040 (1.767)	5.045 (2.984)	-2.355	-3.615	-1.182	<0.0001	23199.0000

Table S20C. How burdensome did you find the brief cognitive task?

Timepoint	Ref. Group	Ref. N	Comp. Group	Comp. N	Ref. Mean (SD)	Comp. Mean (SD)	Comp. vs Ref. Estimate	Lower 95% CrI	Upper 95% CrI	BF (ICTI < Comp)	BF (ICTI > Comp)
Baseline	ICTI	25	AC	22	6.560 (1.873)	6.045 (3.109)	-0.091	-1.170	0.978	0.7689	1.3005

Table S20D. How distressing did you find the brief cognitive task?

Timepoint	Ref. Group	Ref. N	Comp. Group	Comp. N	Ref. Mean (SD)	Comp. Mean (SD)	Comp. vs Ref. Estimate	Lower 95% CrI	Upper 95% CrI	BF (ICTI < Comp)	BF (ICTI > Comp)
Baseline	ICTI	25	AC	22	8.240 (1.877)	9.364 (1.177)	1.580	0.405	2.828	265.6667	0.0038

Table S20E. Overall, how acceptable did you find the brief cognitive task?

Timepoint	Ref. Group	Ref. N	Comp. Group	Comp. N	Ref. Mean (SD)	Comp. Mean (SD)	Comp. vs Ref. Estimate	Lower 95% CrI	Upper 95% CrI	BF (ICTI < Comp)	BF (ICTI > Comp)
Baseline	ICTI	25	AC	22	7.920 (2.326)	6.500 (2.790)	-1.184	-2.305	-0.097	0.0166	60.1170

Table S20F. If you were having intrusive memories in the future, how willing would you be to use the brief cognitive task if it was offered to you as something that would help?

Timepoint	Ref. Group	Ref. N	Comp. Group	Comp. N	Ref. Mean (SD)	Comp. Mean (SD)	Comp. vs Ref. Estimate	Lower 95% CrI	Upper 95% CrI	BF (ICTI < Comp)	BF (ICTI > Comp)
Baseline	ICTI	25	AC	22	8.080 (1.977)	5.864 (3.655)	-1.254	-2.363	-0.175	0.0111	90.1233

Table S20G. If a colleague or friend was having intrusive memories, how confident would you be in recommending the brief cognitive task to them?

Timepoint	Ref. Group	Ref. N	Comp. Group	Comp. N	Ref. Mean (SD)	Comp. Mean (SD)	Comp. vs Ref. Estimate	Lower 95% CrI	Upper 95% CrI	BF (ICTI < Comp)	BF (ICTI > Comp)
Baseline	ICTI	25	AC	22	7.680 (2.212)	5.318 (3.578)	-1.467	-2.610	-0.368	0.0046	218.2817

Table S20H. How much do you feel that this brief cognitive task could be used within NHS Trusts/healthcare organisations to support staff who have experienced work-related traumatic events?

Timepoint	Ref. Group	Ref. N	Comp. Group	Comp. N	Ref. Mean (SD)	Comp. Mean (SD)	Comp. vs Ref. Estimate	Lower 95% CrI	Upper 95% CrI	BF (ICTI < Comp)	BF (ICTI > Comp)
Baseline	ICTI	25	AC	22	8.000 (2.062)	5.545 (3.306)	-1.660	-2.806	-0.557	0.0013	777.5235

Note. Bayes factors (BF) quantifying the evidence for an alternative hypothesis (H_1) over the null hypothesis (H_0) are presented to illustrate evidence in favour of positive and negative treatment effects of the ICTI at all follow-up time-points (weeks 4, 12 and 24). The ICTI arm is treated as the reference group with the AC arm treated as the comparator. The estimate shows the expected difference in the model between the ICTI and AC groups. Negative estimates > 0 show model estimated scores of were lower in the AC arm relative to the ICTI. Means (SD) are presented at each time-point for the analysis population included in the model.

Abbreviations: ICTI = Imagery-Competing Task Intervention; AC = active control; TAU = treatment-as-usual; Ref. = reference [group]; Comp. = comparator [group]; SD = standard deviation; CrI = credible interval; BF = Bayes factor; NHS = UK National Health Service.

Table S21: Other outcomes. ICTI/AC Self-guided usage. Descriptive summary statistics.

Variable	N	Mean (SD)	Median (Q1-Q3, IQR)	Sum
Number of times either intervention used (Guided session – Week 24, inclusive)				Sum of Task Uses
ICTI arm	34	6.91 (7.40)	6.00 (2.25-7.75, 5.50)	235
AC arm	35	5.57 (9.55)	2.00 (1.00-5.00, 4.00)	195
Duration of engagement in gameplay* or music-listening task (Minutes:Seconds)				
ICTI arm, guided session only	34	20:00 (02:17)	20:16 (20:10-20:23, 00:13)	34
ICTI arm, guided session - Week 24 (inclusive)	34	20:00 (03:00)	20:25 (20:11-21:11, 01:00)	235
AC arm, guided session only	35	19:19 (01:57)	20:00 (20:00-20:00, 00:00)	35
AC arm, guided session - Week 24 (inclusive)	35	17:38 (03:37)	20:00 (15:54-20:00, 04:06)	195
Number of unique memories listed (ICTI arm only)				Sum of Unique IMs Listed
Guided session	34	4.71 (2.43)	4.00 (3.00-6.00, 3.00)	160
Total (including memories added to list)	34	5.32 (2.81)	4.50 (4.00-6.75, 2.75)	181
Cumulative percentage of memories targeted (ICTI arm only), %				Sum of Unique IMs Targeted
Guided session - Week 24 (inclusive)	34	63.99 (29.37)	67.95 (33.33-96.43, 63.10)	113
Number of times ICTI completed per targeted intrusive memory				
Guided session - Week 24 (inclusive)	113	2.08 (2.31)	1.00 (1.00-2.00, 1.00)	

Note. The gameplay component of the ICTI task (i.e., the duration of time for which participants played the game Tetris® using mental rotation) was captured in minutes and seconds. Moreover, despite instructions to play for approximately 20-minutes, there was no limit on the length of time for which participants could play Tetris®. In contrast, the duration of the music-listening component of the AC task could not exceed the duration of the piece (approximately 20-minutes) and duration of music-listening was captured only in whole integer minutes.

Two participants did not adhere to the researcher guided session (n=1 ICTI, did not complete 20 uninterrupted minutes of TETRIS® gameplay using mental rotation due to technological issues; n=1 AC, did not complete to the music listening task for sufficient duration).

N values for the sample over which data are reported includes the total number of participants who attended their initial guided session.

In the ICTI guided-session, participants listed a median of 4.0 unique IMs (IQR=3.0-6.00) and used ICTI to target a median of 68% of these.

*Tetris® gameplay constitutes only one part of adherence to the ICTI and is illustrated due to comparability to AC task (see Supplementary Methods).

Abbreviations: ICTI = Imagery-Competing-Task-Intervention; AC = Active Control.

Table S22. Other outcomes. Changes to health and work. Descriptive summary statistics

	Week 4				Week 12				Week 24			
**Variable**	**All**	**TAU**	**AC**	**ICTI**	**All**	**TAU**	**AC**	**ICTI**	**All**	**TAU**	**AC**	**ICTI**
N	67	17	26	24	64	19	24	21	65	16	24	25
Have you experienced or witnessed any new work-related traumatic events? Remember, a traumatic event is defined as an event that involved actual or risk of death, serious injury, or sexual violence for you or someone else., n (%)												
Yes	21 (31.3%)	9 (52.9%)	6 (23.1%)	6 (25.0%)	19 (29.7%)	8 (42.1%)	3 (12.5%)	8 (38.1%)	17 (26.2%)	4 (25.0%)	8 (33.3%)	5 (20.0%)
No	46 (68.7%)	8 (47.1%)	20 (76.9%)	18 (75.0%)	45 (70.3%)	11 (57.9%)	21 (87.5%)	13 (61.9%)	48 (73.8%)	12 (75.0%)	16 (66.7%)	20 (80.0%)
A traumatic or tragic death of a patient, n (%)												
Yes	9 (42.9%)	5 (55.6%)	1 (16.7%)	3 (50.0%)	8 (42.1%)	4 (50.0%)	1 (33.3%)	3 (37.5%)	10 (58.8%)	3 (75.0%)	3 (37.5%)	4 (80.0%)
No	12 (57.1%)	4 (44.4%)	5 (83.3%)	3 (50.0%)	11 (57.9%)	4 (50.0%)	2 (66.7%)	5 (62.5%)	7 (41.2%)	1 (25.0%)	5 (62.5%)	1 (20.0%)
A severe or unsuccessful resuscitation, n (%)												
Yes	7 (33.3%)	3 (33.3%)	1 (16.7%)	3 (50.0%)	7 (36.8%)	4 (50.0%)	1 (33.3%)	2 (25.0%)	7 (41.2%)	2 (50.0%)	4 (50.0%)	1 (20.0%)
No	14 (66.7%)	6 (66.7%)	5 (83.3%)	3 (50.0%)	12 (63.2%)	4 (50.0%)	2 (66.7%)	6 (75.0%)	10 (58.8%)	2 (50.0%)	4 (50.0%)	4 (80.0%)
Witnessing events surrounding colleague who has fallen ill or died of COVID-19, n (%)												
Yes	0 (0.0%)	0 (0.0%)	0 (0.0%)	0 (0.0%)	1 (5.3%)	1 (12.5%)	0 (0.0%)	0 (0.0%)	1 (5.9%)	0 (0.0%)	1 (12.5%)	0 (0.0%)
No	21 (100.0%)	9 (100.0%)	6 (100.0%)	6 (100.0%)	18 (94.7%)	7 (87.5%)	3 (100.0%)	8 (100.0%)	16 (94.1%)	4 (100.0%)	7 (87.5%)	5 (100.0%)
Situation where the care of a patient failed or did not go as planned, n (%)												
Yes	7 (33.3%)	4 (44.4%)	2 (33.3%)	1 (16.7%)	13 (68.4%)	5 (62.5%)	1 (33.3%)	7 (87.5%)	10 (58.8%)	3 (75.0%)	4 (50.0%)	3 (60.0%)
No	14 (66.7%)	5 (55.6%)	4 (66.7%)	5 (83.3%)	6 (31.6%)	3 (37.5%)	2 (66.7%)	1 (12.5%)	7 (41.2%)	1 (25.0%)	4 (50.0%)	2 (40.0%)
Threats or violence against healthcare professionals, n (%)												
Yes	5 (23.8%)	1 (11.1%)	3 (50.0%)	1 (16.7%)	3 (15.8%)	1 (12.5%)	1 (33.3%)	1 (12.5%)	7 (41.2%)	1 (25.0%)	4 (50.0%)	2 (40.0%)
No	16 (76.2%)	8 (88.9%)	3 (50.0%)	5 (83.3%)	16 (84.2%)	7 (87.5%)	2 (66.7%)	7 (87.5%)	10 (58.8%)	3 (75.0%)	4 (50.0%)	3 (60.0%)

Variable	Week 4				Week 12				Week 24			
	All	TAU	AC	ICTI	All	TAU	AC	ICTI	All	TAU	AC	ICTI
Event involving sudden increased risk of COVID-19 infection, n (%)												
Yes	2 (9.5%)	1 (11.1%)	1 (16.7%)	0 (0.0%)	5 (26.3%)	3 (37.5%)	0 (0.0%)	2 (25.0%)	4 (23.5%)	1 (25.0%)	2 (25.0%)	1 (20.0%)
No	19 (90.5%)	8 (88.9%)	5 (83.3%)	6 (100.0%)	14 (73.7%)	5 (62.5%)	3 (100.0%)	6 (75.0%)	13 (76.5%)	3 (75.0%)	6 (75.0%)	4 (80.0%)
A traumatic or tragic event where a patient reminded you of yourself, a family member or friend, n (%)												
Yes	11 (52.4%)	3 (33.3%)	4 (66.7%)	4 (66.7%)	10 (52.6%)	2 (25.0%)	2 (66.7%)	6 (75.0%)	8 (47.1%)	2 (50.0%)	4 (50.0%)	2 (40.0%)
No	10 (47.6%)	6 (66.7%)	2 (33.3%)	2 (33.3%)	9 (47.4%)	6 (75.0%)	1 (33.3%)	2 (25.0%)	9 (52.9%)	2 (50.0%)	4 (50.0%)	3 (60.0%)
Event involving extremely distressed/grieving relatives of patients, n (%)												
Yes	8 (38.1%)	4 (44.4%)	2 (33.3%)	2 (33.3%)	6 (31.6%)	3 (37.5%)	1 (33.3%)	2 (25.0%)	3 (17.6%)	0 (0.0%)	1 (12.5%)	2 (40.0%)
No	13 (61.9%)	5 (55.6%)	4 (66.7%)	4 (66.7%)	13 (68.4%)	5 (62.5%)	2 (66.7%)	6 (75.0%)	14 (82.4%)	4 (100.0%)	7 (87.5%)	3 (60.0%)
Being faced with suicide / suicide attempt, n (%)												
Yes	6 (28.6%)	4 (44.4%)	1 (16.7%)	1 (16.7%)	8 (42.1%)	2 (25.0%)	0 (0.0%)	6 (75.0%)	6 (35.3%)	0 (0.0%)	4 (50.0%)	2 (40.0%)
No	15 (71.4%)	5 (55.6%)	5 (83.3%)	5 (83.3%)	11 (57.9%)	6 (75.0%)	3 (100.0%)	2 (25.0%)	11 (64.7%)	4 (100.0%)	4 (50.0%)	3 (60.0%)
Other, n (%)												
Yes	1 (4.8%)	0 (0.0%)	1 (16.7%)	0 (0.0%)	1 (5.3%)	1 (12.5%)	0 (0.0%)	0 (0.0%)	1 (5.9%)	1 (25.0%)	0 (0.0%)	0 (0.0%)
No	20 (95.2%)	9 (100.0%)	5 (83.3%)	6 (100.0%)	18 (94.7%)	7 (87.5%)	3 (100.0%)	8 (100.0%)	16 (94.1%)	3 (75.0%)	8 (100.0%)	5 (100.0%)
How many new work-related traumatic events have you experienced/witnessed?												
Mean (SD)	0.90 (1.75)	1.53 (2.67)	0.62 (1.20)	0.75 (1.36)	0.98 (1.96)	1.05 (1.93)	0.46 (1.14)	1.52 (2.58)	0.94 (2.19)	0.75 (1.39)	1.04 (2.01)	0.96 (2.76)
Median (IQR)	0.00 (0.00 - 1.00)	0.00 (0.00 - 2.00)	0.00 (0.00 - 1.00)	0.00 (0.00 - 1.25)	0.00 (0.00 - 1.00)	0.00 (0.00 - 1.50)	0.00 (0.00 - 0.00)	0.00 (0.00 - 3.00)	0.00 (0.00 - 1.00)	0.00 (0.00 - 1.00)	0.00 (0.00 - 1.25)	0.00 (0.00 - 0.00)
Min - Max	0 - 10	0 - 10	0 - 5	0 - 5	0 - 10	0 - 8	0 - 5	0 - 10	0 - 10	0 - 4	0 - 8	0 - 10
How many new traumatic events that were not work-related have you experienced/witnessed (e.g., serious accident, assault, injury or illness)?												

	Week 4				Week 12				Week 24			
**Variable**	**All**	**TAU**	**AC**	**ICTI**	**All**	**TAU**	**AC**	**ICTI**	**All**	**TAU**	**AC**	**ICTI**
Mean (SD)	0.12 (0.37)	0.06 (0.24)	0.15 (0.46)	0.13 (0.34)	0.34 (0.74)	0.37 (0.76)	0.17 (0.48)	0.52 (0.93)	0.20 (0.54)	0.13 (0.34)	0.29 (0.75)	0.16 (0.37)
Median (IQR)	0.00 (0.00 - 0.00)	0.00 (0.00 - 0.00)	0.00 (0.00 - 0.00)	0.00 (0.00 - 0.00)	0.00 (0.00 - 0.00)	0.00 (0.00 - 0.50)	0.00 (0.00 - 0.00)	0.00 (0.00 - 1.00)	0.00 (0.00 - 0.00)	0.00 (0.00 - 0.00)	0.00 (0.00 - 0.00)	0.00 (0.00 - 0.00)
Min - Max	0 - 2	0 - 1	0 - 2	0 - 1	0 - 3	0 - 3	0 - 2	0 - 3	0 - 3	0 - 1	0 - 3	0 - 1
Have you experienced any additional stressful life events e.g. relationship problems, financial problems, work problems, health problems, bereavements, etc.?, n (%)												
Yes	37 (55.2%)	8 (47.1%)	14 (53.8%)	15 (62.5%)	37 (57.8%)	11 (57.9%)	15 (62.5%)	11 (52.4%)	35 (53.8%)	10 (62.5%)	12 (50.0%)	13 (52.0%)
No	30 (44.8%)	9 (52.9%)	12 (46.2%)	9 (37.5%)	27 (42.2%)	8 (42.1%)	9 (37.5%)	10 (47.6%)	30 (46.2%)	6 (37.5%)	12 (50.0%)	12 (48.0%)
Have you received any new treatments, including other treatments in relation to the traumatic event e.g. medication or psychological treatment?, n (%)												
Yes	11 (16.4%)	4 (23.5%)	3 (11.5%)	4 (16.7%)	8 (12.5%)	3 (15.8%)	2 (8.3%)	3 (14.3%)	10 (15.4%)	5 (31.3%)	0 (0.0%)	5 (20.0%)
No	56 (83.6%)	13 (76.5%)	23 (88.5%)	20 (83.3%)	56 (87.5%)	16 (84.2%)	22 (91.7%)	18 (85.7%)	55 (84.6%)	11 (68.8%)	24 (100.0%)	20 (80.0%)
Have you had any untoward medical occurrences or other problems?, n (%)												
Yes	9 (13.4%)	0 (0.0%)	3 (11.5%)	6 (25.0%)	8 (12.5%)	3 (15.8%)	3 (12.5%)	2 (9.5%)	11 (16.9%)	5 (31.3%)	4 (16.7%)	2 (8.0%)
No	58 (86.6%)	17 (100.0%)	23 (88.5%)	18 (75.0%)	56 (87.5%)	16 (84.2%)	21 (87.5%)	19 (90.5%)	54 (83.1%)	11 (68.8%)	20 (83.3%)	23 (92.0%)
Do you work in the same job?, n (%)												
Yes	61 (91.0%)	14 (82.4%)	23 (88.5%)	24 (100.0%)	57 (89.1%)	18 (94.7%)	19 (79.2%)	20 (95.2%)	60 (92.3%)	15 (93.8%)	20 (83.3%)	25 (100.0%)
No	6 (9.0%)	3 (17.6%)	3 (11.5%)	0 (0.0%)	7 (10.9%)	1 (5.3%)	5 (20.8%)	1 (4.8%)	5 (7.7%)	1 (6.3%)	4 (16.7%)	0 (0.0%)
Do you work the same number of hours per week?, n (%)												
Yes	57 (85.1%)	15 (88.2%)	21 (80.8%)	21 (87.5%)	53 (82.8%)	18 (94.7%)	17 (70.8%)	18 (85.7%)	54 (83.1%)	16 (100.0%)	17 (70.8%)	21 (84.0%)
No	10 (14.9%)	2 (11.8%)	5 (19.2%)	3 (12.5%)	11 (17.2%)	1 (5.3%)	7 (29.2%)	3 (14.3%)	11 (16.9%)	0 (0.0%)	7 (29.2%)	4 (16.0%)

Notes. Participants reported changes to their health and work at weeks 4, 12, and 24. Items in this questionnaire covered the emergence of new traumatic events related or unrelated to work (presented in Table 1), additional life stressors, new treatments, and alterations in job roles or working hours.

All items consist of dichotomous (‘yes’/ ‘no’) responses. We present the frequency and proportion of ‘yes’ responses to items assessing changes to health and work status after baseline at weeks 4, 12 and 24.

Abbreviations: ICTI = Imagery-Competing Task Intervention; AC = active control; TAU = treatment-as-usual; n = number.

Table S23A. Other outcomes. Credibility and expectancy of the intervention. Bayesian results of between groups comparisons of individual items

Participants completed the Credibility and Expectancy Questionnaire[13] (CEQ) after being informed of treatment allocation. CEQ Items 1, 2, 3, and 5 were assessed on a 9-point scale (response range 1-9) while Items 4 and 6 were assessed on an 11-point scale (response range of 0-100%). The six items of the CEQ were modelled as a cumulative regression model with outcomes modelled using a single fixed effect of ‘arm’.

Table S23A.i. CEQ Item 1. At this point, how logical does the intervention offered to you seem?

Timepoint	Ref. Group	Ref. N	Comp. Group	Comp. N	Ref. Mean (SD)	Comp. Mean (SD)	Comp. vs Ref. Estimate	Lower 95% CrI	Upper 95% CrI	BF (ICTI < Comp)	BF (ICTI > Comp)
Baseline	ICTI	34	AC	35	5.941 (1.890)	6.800 (1.346)	0.910	0.065	1.767	58.5788	0.0171
Baseline	ICTI	34	TAU	19	5.941 (1.890)	4.421 (2.545)	-1.544	-2.681	-0.429	0.0031	327.6119

Table S23A.ii. CEQ Item 2. At this point, how successful do you think this intervention will be in reducing your IMs?

Timepoint	Ref. Group	Ref. N	Comp. Group	Comp. N	Ref. Mean (SD)	Comp. Mean (SD)	Comp. vs Ref. Estimate	Lower 95% CrI	Upper 95% CrI	BF (ICTI < Comp)	BF (ICTI > Comp)
Baseline	ICTI	34	AC	35	5.441 (1.941)	5.771 (1.457)	0.360	-0.504	1.228	3.8446	0.2601
Baseline	ICTI	34	TAU	19	5.441 (1.941)	2.895 (1.663)	-2.875	-4.117	-1.699	<0.0001	Inf

Table S23A.iii. CEQ Item 3. How confident would you be in recommending this intervention to a friend who experiences similar problems?

Timepoint	Ref. Group	Ref. N	Comp. Group	Comp. N	Ref. Mean (SD)	Comp. Mean (SD)	Comp. vs Ref. Estimate	Lower 95% CrI	Upper 95% CrI	BF (ICTI < Comp)	BF (ICTI > Comp)
Baseline	ICTI	34	AC	35	5.353 (2.228)	6.171 (2.007)	0.721	-0.132	1.589	19.5492	0.0512
Baseline	ICTI	34	TAU	19	5.353 (2.228)	2.842 (2.292)	-2.360	-3.566	-1.213	<0.0001	57999.0000

Table S23A.iv. CEQ Item 4. By the end of the intervention period (four weeks), how much improvement in your IMs do you think will occur?

Timepoint	Ref. Group	Ref. N	Comp. Group	Comp. N	Ref. Mean (SD)	Comp. Mean (SD)	Comp. vs Ref. Estimate	Lower 95% CrI	Upper 95% CrI	BF (ICTI < Comp)	BF (ICTI > Comp)
Baseline	ICTI	34	AC	35	5.000 (2.498)	5.800 (2.055)	0.546	-0.305	1.402	8.6990	0.1150
Baseline	ICTI	34	TAU	19	5.000 (2.498)	1.947 (2.592)	-3.117	-4.483	-1.845	<0.0001	Inf

Table S23A.v. CEQ Item 5. At this point, how much do you really feel that the intervention will help you to reduce your IMs?

Timepoint	Ref. Group	Ref. N	Comp. Group	Comp. N	Ref. Mean (SD)	Comp. Mean (SD)	Comp. vs Ref. Estimate	Lower 95% CrI	Upper 95% CrI	BF (ICTI < Comp)	BF (ICTI > Comp)
Baseline	ICTI	34	AC	35	5.353 (2.360)	5.857 (1.817)	0.274	-0.576	1.124	2.8043	0.3566
Baseline	ICTI	34	TAU	19	5.353 (2.360)	2.263 (1.727)	-3.059	-4.318	-1.857	<0.0001	Inf

Table S23A.vi. CEQ Item 6. By the end of the intervention period (four weeks), how much improvement in your IMs do you really feel will occur?

Timepoint	Ref. Group	Ref. N	Comp. Group	Comp. N	Ref. Mean (SD)	Comp. Mean (SD)	Comp. vs Ref. Estimate	Lower 95% CrI	Upper 95% CrI	BF (ICTI < Comp)	BF (ICTI > Comp)
Baseline	ICTI	34	AC	35	5.059 (2.964)	5.829 (2.281)	0.513	-0.341	1.374	7.3712	0.1357
Baseline	ICTI	34	TAU	19	5.059 (2.964)	1.526 (2.091)	-3.013	-4.318	-1.793	<0.0001	Inf

Note. Bayes factors (BF) quantifying the evidence for an alternative hypothesis (H_1) over the null hypothesis (H_0) are presented to illustrate evidence in favour of positive and negative effects of the ICTI for CEQ items. The ICTI arm is treated as the reference group with the AC and TAU arms treated as the comparators. The estimate shows the expected difference between the groups in the model. For the six ordinal items, positive estimates > 0 show model estimated scores of were greater in the comparator arms relative to the ICTI. Means (SD) are presented at each timepoint for the analysis population included in the model.

Abbreviations: CEQ = credibility and expectancy questionnaire; IM = intrusive memory; ICTI = Imagery-Competing Task Intervention; AC = active control; TAU = treatment-as-usual; Ref. = reference [group]; Comp. = comparator [group]; SD = standard deviation; CrI = credible interval; BF = Bayes factor.

Table S23B. Post-hoc Analysis. Total Score of Credibility and Expectancy (CEQ). Bayesian results of between groups comparisons.

Timepoint	Ref. Group	Ref. N	Comp. Group	Comp. N	Ref. Mean (SD)	Comp. Mean (SD)	Comp. vs Ref. Estimate	Lower 95% CrI	Upper 95% CrI	BF (ICTI < Comp)	BF (ICTI > Comp)
Baseline	ICTI	34	AC	35	32.135 (11.424)	35.903 (8.226)	3.944	-0.669	8.562	20.0986	0.0498
Baseline	ICTI	34	TAU	19	32.135 (11.424)	17.200 (10.281)	-13.900	-19.321	-8.428	<0.0001	Inf

Note. Participants completed the CEQ (credibility and expectancy questionnaire) after being informed of treatment allocation. The total score was calculated by converting Items 4 and 6 from an 11-point scale (response range of 0-100%) to the same 9-point scale (response range 1-9) as used for the other items and then summing all items. The CEQ total score was treated as a continuous variable and was analysed via a linear regression model with a normal distribution.

Bayes factors (BF) quantifying the evidence for an alternative hypothesis (H_1) over the null hypothesis (H_0) are presented to illustrate evidence in favour of positive and negative effects of the ICTI for CEQ total score. The ICTI arm is treated as the reference group with the AC and TAU arms treated as the comparators. The estimate shows the expected difference between the groups in the model. Positive estimates > 0 show model estimated scores of were greater in the comparator arms relative to the ICTI. Means (SD) are presented at each timepoint for the analysis population included in the model. While CEQ total score was omitted from the SAP in error, and for comparison with the GAINS-01 paper (Iyadurai et al, 2023) between-groups Bayesian analysis of the CEQ total score was performed here as a post-hoc analysis.

Abbreviations: CEQ = credibility and expectancy questionnaire; ICTI = Imagery-Competing Task Intervention; AC = active control; TAU = treatment-as-usual; Ref. = reference [group]; Comp. = comparator [group]; SD = standard deviation; CrI = credible interval; BF = Bayes factor.

Table S24. Adverse Events, Serious Adverse Events and Concomitant Treatments Started During the In-Study Period

Table S24A. Table of Adverse Events

		Frequency of Adverse Events															
		ICTI				AC				TAU				All Groups Combined			
**Category**	**Code/Short Description**	**Pre-Treat.**	**GS-Wk4**	**Wk4-12**	**Wk12-24**	**Pre-Treat.**	**GS-Wk4**	**Wk4-12**	**Wk12-24**	**Pre-Treat.**	**GS-Wk4**	**Wk4-12**	**Wk12-24**	**Pre-Treat.**	**GS-Wk4**	**Wk4-12**	**Wk12-24**
Respiratory illness	Bronchitis / chest infection / Cold				1			2	1							2	2
	Coronavirus	2			1	1	1	1	1					3	1	1	2
Pain related	Neck pain		1												1		
	Stress headaches		1												1		
	Nerve pain in leg								1								1
	Hip pain											1				1	
	Abdominal pain											1				1	
	Knee bursitis												1				1
	Joint pain						1								1		
	Post-operative pain following elective bilateral lumbar nerve root block procedure				1												1
	Lower back pain and paresthesia following (pre-existing) cervical myelopathy						1								1		
	Dysmenorrhea				1												1
Physical injury	Fractured foot											1				1	
	Labrum tear in hip			1												1	
	Fractured rib												1				1
	Knee injury												1				1
Skin related	Dermatitis			1												1	
	Rash (Lyme's disease)		1												1		
	'Flare up' of pre-existing dermatological condition						1		1*						1		
	Eczema		1												1		
Other illnesses	Hot sweats								1								1

		Frequency of Adverse Events															
		ICTI				AC				TAU				All Groups Combined			
	Minor infection		1												1		
	Unspecified illness					1								1			
	Shingles	1												1			
	Unspecified virus	1												1			
	Blocked tear duct				1												1
	Migraine					1								1			
	Iron and vitamin d deficiency causing excessive sleep and fatigue												1				1
	Low mood										1				1		
	Deterioration of long term condition (following hand surgery)	1												1			
	Deteriorating knee function due to previous bilteral achiles tendon ruptures			1												1	
	Worsening menopause symptoms								1								1
	Decline in pre-existing condition related to nerve damage				1												1
	Elevated triglycerides (related to pre-existing steroid induced type 2 diabetes)						1								1		
	Crohn’s disease symptoms												1				1
	Dehydration / low blood pressure												1				1
New diagnoses	Diagnosis of active Crohn’s disease			1												1	
	Diagnosis of ADHD				1												1
	Diagnosis of unspecified auto-immune disease				1												1
	Diagnosis of coronary artery disease							1								1	
Adverse Events potentially related to procedures	Difficulties recording intrusive memories in diary		1					1							1	1	
	Total all adverse events, by arm	5	6	4	8	3	5	5	5	0	1	3	6	8	12	12	19

Note., Adverse events were reported by participants during routine follow-up assessments at weeks 4, 12, and 24 by responding to a questionnaire item with a free-text response (“Have you received any new treatments, including other treatments in relation to the traumatic event e.g. medication or psychological treatment?”), or were reported during contact with researchers. Events are grouped by category and are presented according to the time-point during which they occurred.

In total, 51 adverse events were recorded and the percentage of participants in any arm reporting an adverse event was 37.37%. Over the in-study period 23 adverse events were recorded for 15 ICTI participants; 18 adverse events were recorded for 13 AC participants; and 10 adverse events were recorded for 9 TAU participants.

In the ICTI arm, 1 adverse event was related to either study procedures, potentially to the intervention or both (recording of intrusive memories increased prominence of IMs causing distress). Likewise, in the AC arm, 1 adverse event related to recording IMs (monitoring IMs causing frequency of IMs to increase) was deemed related to study procedures.

No statistical tests were planned or conducted to test for differences in the frequency of adverse events between study arms.

*One participant reported two instances of an adverse event (eczema) which were attributed to the same underlying condition and therefore were counted as a single adverse event.

Abbreviations: 'Pre-Treat.' = Prior to receiving allocated treatment (or equivalent in TAU); Post-treat' = After receiving allocated treatment (or equivalent in TAU); GS = Guided Session; ICTI = Imagery-Competing-Task-Intervention; AC = active control; TAU = treatment-as-usual.

Table S24B. Table of serious adverse events

	Frequency of Serious Adverse Events and Outcome			
**Details of SAE**	**ICTI**	**AC**	**TAU**	**Outcome of SAE**
Hospitalised due to endometriosis	1			Continued in the study per protocol
Hospitalised due to kidney infection	1			Continued in the study per protocol
Hospitalised due to pneumonia		1		Continued in the study per protocol
Hospitalised due to post-operative infection (hysteroscopy)		1		Continued in the study per protocol
Hospitalised due to septic shock	1			Withdrew from the study
Surgery for cervical myelopathy		1		Continued in the study per protocol
Total by arm	3	3	0	

Note. All serious AEs (SAEs; 3 in ICTI and 3 in AC) were unrelated to study or intervention procedures. No participant reported multiple SAEs

Abbreviations: SAE = serious adverse event; ICTI = Imagery-Competing Task Intervention; AC = active control; TAU = treatment-as-usual.

Table S24C. Table of Concomitant Treatments Started During the In-Study Period

	Frequency of New Concomitant Treatments			
**Short Description**	**ICTI**	**AC**	**TAU**	**Combined**
**Psychotropic Medication**				
Venlafaxine(medication dosage increase)			1	1
Sertraline (issues with mood)			1	1
Lisdexamfetamine (Temporary medication dosage increase)			1	1
Escitalopram (reason not specified)	1			1
**Psychotherapies/Counselling**				
Counselling (reason not given)		1		1
Accessing ‘private therapy’ (reason not specified).			1	1
Trauma-focused peer support (TRiM)			1	1
Mentalisation-based therapy (reason not specified)			1	1
EMDR Therapy (reason not specified)			1	1
**Other Treatments**				
Hormone Replacement Therapy (menopause)		1		1
Painkillers (endometriosis)	1			1
Hormones (endometriosis)	1			1
Physiotherapy (knee injury)			1	1
Physiotherapy (fractured foot)			1	1
GA, MUA and injection, and an MRI (hip pain)			1	1
‘Pain medication’ (fractured rib)			1	1
Metformin (type 2 diabetes)		1		1
Elective Bilateral lumbar nerve root block procedure	1			1
Nortriptyline (post-operative pain)	1			1
Antibiotics (minor infection)	1			1
Antibiotics (Lyme's disease)	1			1
Antibiotics (Eczema)	1			1
Colonoscopy (problems with stomach)	1			1
Surgical stent (blocked tear duct)	1			1
Biopsy (dehydration / low blood pressure)			1	1
Antibiotics for post-operative infection (hysteroscopy)		1		1
Atorvastatin increased to 20mg OD (mild coronary artery disease)		1		1
Aspirin 75mg OD (mild coronary artery disease)		1		1
Total by arm	10	6	12	28

Table S25A. Post-hoc analysis: Participant retention at primary outcome per arm. Bayesian logistic regression

Parameter	Estimate	Est.Error	l-95% CrI	u-95% CrI	Rhat	Bulk_ESS	Tail_ESS
Intercept	-8.88	4.66	-19.24	-1.43	1	51924	57259
Baseline Intrusive Memories	0.02	0.02	-0.02	0.06	1	127328	83225
Age	0.02	0.03	-0.03	0.07	1	124062	86020
Gender (man)	-0.44	0.95	-2.5	1.27	1	112311	66430
Gender (non-binary)	-7.04	6.36	-21.84	2.13	1	93906	63862
Gender (prefer not to answer)	-6.72	6.38	-21.58	2.52	1	90025	61553
Ethnicity (Chinese)	-3.65	7.8	-20.46	9.97	1	93745	77362
Ethnicity (Mixed)	-4.01	7.42	-20.25	8.93	1	82368	73519
Ethnicity (White)	6.67	4.46	-0.21	16.67	1	50554	54280
Ethnicity (Other)	7.02	4.63	-0.63	17.16	1	52167	54086
Hours worked per week	0	0.02	-0.05	0.04	1	116423	83289
Number of work-related traumatic events	0	0	-0.01	0.01	1	147560	78016
Number of not work-related traumatic events	0	0.05	-0.1	0.09	1	105102	82995

Note. Parameter estimates from a Bayesian logistic regression model predicting missingness of outcome data by selected baseline and demographic variables and study arm. BF → 0 for this model vs. the null model (intercept only).

Abbreviations. Est.Error = standard error of estimate; L-95% CI = lower bound of 95% credible interval; U-95% CI = upper bound of 95% credible interval; Rhat = convergence diagnostic; ESS = effective sample size.

Table S25B. Post-hoc analysis: Participant retention at primary outcome. Baseline characteristics for intention to treat (ITT) population split by data missingness at primary outcome, between groups.

	AC			ICTI		
**Variable**	**All**	**Missing**	**Present**	**All**	**Missing**	**Present**
**N,**	39	8	31	40	14	26
**Age, years**						
Mean (SD)	43.29 (10.92)	48.38 (8.05)	41.93 (11.30)	40.18 (10.44)	40.93 (8.88)	39.77 (11.34)
Unknown	1	0	1			
**Gender, n (%)**						
Man	6 (15.4%)	1 (12.5%)	5 (16.1%)	4 (10.0%)	1 (7.1%)	3 (11.5%)
Woman	33 (84.6%)	7 (87.5%)	26 (83.9%)	34 (85.0%)	13 (92.9%)	21 (80.8%)
Gender-variant/non-binary	0 (0.0%)	0 (0.0%)	0 (0.0%)	1 (2.5%)	0 (0.0%)	1 (3.8%)
Prefer not to answer	0 (0.0%)	0 (0.0%)	0 (0.0%)	1 (2.5%)	0 (0.0%)	1 (3.8%)
**Highest level of education, n (%)**						
Master's degree	4 (10.3%)	0 (0.0%)	4 (12.9%)	10 (25.0%)	4 (28.6%)	6 (23.1%)
Bachelor's degree or equivalent	31 (79.5%)	7 (87.5%)	24 (77.4%)	28 (70.0%)	8 (57.1%)	20 (76.9%)
Sixth form or equivalent (to age 18)	3 (7.7%)	1 (12.5%)	2 (6.5%)	2 (5.0%)	2 (14.3%)	0 (0.0%)
Secondary school (to age 16)	1 (2.6%)	0 (0.0%)	1 (3.2%)	0 (0.0%)	0 (0.0%)	0 (0.0%)
**Ethnicity, n (%)**						
Asian	2 (5.1%)	0 (0.0%)	2 (6.5%)	0 (0.0%)	0 (0.0%)	0 (0.0%)
Chinese	0 (0.0%)	0 (0.0%)	0 (0.0%)	1 (2.5%)	0 (0.0%)	1 (3.8%)
Mixed	1 (2.6%)	0 (0.0%)	1 (3.2%)	2 (5.0%)	0 (0.0%)	2 (7.7%)
White	34 (87.2%)	8 (100.0%)	26 (83.9%)	36 (90.0%)	13 (92.9%)	23 (88.5%)
Other	2 (5.1%)	0 (0.0%)	2 (6.5%)	1 (2.5%)	1 (7.1%)	0 (0.0%)
**Marital status, n (%)**						
Single	7 (17.9%)	2 (25.0%)	5 (16.1%)	9 (22.5%)	4 (28.6%)	5 (19.2%)
Living apart from partner	4 (10.3%)	0 (0.0%)	4 (12.9%)	3 (7.5%)	1 (7.1%)	2 (7.7%)
Married or cohabiting	21 (53.8%)	4 (50.0%)	17 (54.8%)	26 (65.0%)	8 (57.1%)	18 (69.2%)
Divorced or separated	6 (15.4%)	1 (12.5%)	5 (16.1%)	2 (5.0%)	1 (7.1%)	1 (3.8%)
Widowed	1 (2.6%)	1 (12.5%)	0 (0.0%)	0 (0.0%)	0 (0.0%)	0 (0.0%)
Other	0 (0.0%)	(0.0%)	(0.0%)	(0.0%)	(0.0%)	(0.0%)
**Hours working per week**						
Mean (SD)	31.72 (14.56)	34.13 (10.01)	31.10 (15.60)	33.81 (13.46)	33.57 (15.34)	33.94 (12.65)
**Time as a healthcare professional, years**						

Variable	AC			ICTI		
	All	Missing	Present	All	Missing	Present
Mean (SD)	17.69 (12.40)	17.63 (11.10)	17.71 (12.88)	15.58 (9.36)	17.29 (11.15)	14.65 (8.34)
**Employment status, n (%)**						
Working full time	27 (69.2%)	7 (87.5%)	20 (64.5%)	30 (75.0%)	11 (78.6%)	19 (73.1%)
Working part time	6 (15.4%)	1 (12.5%)	5 (16.1%)	5 (12.5%)	1 (7.1%)	4 (15.4%)
Sick leave	2 (5.1%)	0 (0.0%)	2 (6.5%)	1 (2.5%)	0 (0.0%)	1 (3.8%)
Student	3 (7.7%)	0 (0.0%)	3 (9.7%)	0 (0.0%)	0 (0.0%)	0 (0.0%)
Retired	1 (2.6%)	0 (0.0%)	1 (3.2%)	0 (0.0%)	0 (0.0%)	0 (0.0%)
Other	0 (0.0%)	0 (0.0%)	0 (0.0%)	4 (10.0%)	2 (14.3%)	2 (7.7%)
**Frequency of Intrusive Memories (Baseline)**						
Mean (SD)	13.03 (9.58)	15.25 (7.78)	12.45 (10.02)	15.70 (13.48)	19.43 (19.98)	13.69 (7.99)
Median (IQR)	9.00 (6.50 - 16.00)	14.50 (11.75 - 18.25)	9.00 (5.50 - 13.00)	11.50 (9.00 - 18.00)	13.50 (10.00 – 17.00)	11.50 (7.50 - 18.00)
Min - Max	3-37	4-29	3-37	4-84	5-84	4-32

Note. Mean (SD) and n (%) are presented with percentages calculated according to the number of participants with available data in the ITT population (n=99). Missing data for age for 1 participant in AC (‘present’ primary outcome data subgroup). Subgroup data are not presented for the TAU arm due to only one participant not providing week 4 data and consequently being individually identifiable.

ITT=Intention to treat; AC=Active Control; ICTI=Imagery-Competing Task Intervention; TAU=Treatment-As-Usual; SD=standard deviation; n=number.

Table S25C. Post-hoc analysis: Participant retention at primary outcome. Baseline characteristics for intention to treat (ITT) population split by data missingness at primary outcome, across groups.

	Present	Missing
**Variable**	**All**	**All**
**N,**	76	23
**ARM, n (%)**		
TAU	19 (25.0%)	1 (4.3%)
AC	31 (40.8%)	8 (34.8%)
ICTI	26 (34.2%)	14 (60.9%)
**Gender, n (%)**		
Gender-variant/non-binary	1 (1.3%)	
Man	10 (13.2%)	2 (8.7%)
Prefer not to answer	1 (1.3%)	
Woman	64 (84.2%)	21 (91.3%)
**Age, years**		
Mean (SD)	40.53 (10.52)	43.17 (9.22)
Unknown	1	
**Highest level of education, n (%)**		
Master's degree	17 (22.4%)	4 (17.4%)
Bachelor's degree or equivalent	56 (73.7%)	16 (69.6%)
Sixth form or equivalent (to age 18)	2 (2.6%)	3 (13.0%)
Secondary school (to age 16)	1 (1.3%)	0 (0.0%)
**Ethnicity, n (%)**		
Asian	3 (3.9%)	0 (0.0%)
Chinese	1 (1.3%)	0 (0.0%)
Mixed	3 (3.9%)	0 (0.0%)
White	67 (88.2%)	22 (95.7%)
Other	2 (2.6%)	1 (4.3%)
**Marital status, n (%)**		
Divorced or separated	9 (11.8%)	2 (8.7%)
Married or cohabiting	47 (61.8%)	13 (56.5%)
Living apart from partner	6 (7.9%)	1 (4.3%)
Single	14 (18.4%)	6 (26.1%)
Widowed	0 (0.0%)	1 (4.3%)
**Hours working per week**		
Mean (SD)	34.18 (13.10)	33.93 (13.10)
**Time as healthcare professional, years**		
Mean (SD)	16.20 (10.80)	17.13 (10.70)
**Employment status, n (%)**		
Working full time	55 (72.4%)	19 (82.6%)
Working part time	12 (15.8%)	2 (8.7%)
Student	3 (3.9%)	0 (0.0%)
Retired	1 (1.3%)	0 (0.0%)
Sick leave	3 (3.9%)	0 (0.0%)
Other	2 (2.6%)	2 (8.7%)
**Frequency of Intrusive Memories (Baseline)**		
Mean (SD)	13.38 (11.42)	17.39 (16.29)
Median (IQR)	9.00 (5.75 - 18.00)	14.00 (9.50 - 17.00)
Min - Max	3-75	4-84

Note. Mean (SD) or n (%) are presented with percentages calculated according to the number of participants with available data in the ITT population (n=99). Missing data for age for 1 participant in AC (‘present’ primary outcome data subgroup).

ITT=Intention to treat; AC=Active Control; ICTI=Imagery-Competing Task Intervention; TAU=Treatment-As-Usual; SD=standard deviation; n=number.

Table S26. Imputed IM daily diary missing data (weeks 4, 12 and 24)

Week	Arm	Submitted intrusive memory daily diary data	Intrusive memory daily diary data with imputation
Week 4	Active Control	0,2,0,0,NaN,NaN,NaN	0,2,0,0,0,0,0
Week 12	Active Control	0,0,1,0,0,NaN,NaN	0,0,1,0,0,0,0
Week 12	ICTI	0,0,0,NaN,NaN,NaN,NaN	0,0,0,0,0,0,0
Week 12	Active Control	1,2,0,2,NaN,NaN,NaN	1,2,0,2,0,0,0
Week 24	Treatment-As-Usual	1,2,3,1,1, NaN, NaN	1,2,3,1,1,1,0
Week 24	Active Control	NaN,NaN,NaN,NaN,0,0,2	1,0,0,0,0,0,2
Week 24	Treatment-As-Usual	NaN,NaN,NaN,NaN,NaN,NaN,0	2,0,0,0,0,0,0

Note. Here we present the seven instances where IM daily diary data were partially completed, detailing the data that was provided by participants, the data that was missing, and the results of the imputation. At weeks 4, 12, and 24, if participants completed at least one day of the IM daily diary then remaining missing days were imputed. As the IMDD data is collected sequentially over time, we used time series methods and an expectation maximisation algorithm to impute missing values. Initial missing values were imputed by taking expectations across a participant's available diary data. Using Poisson likelihood and correlated errors, we maximise over this 'full' data set to provide updated expected values for the missing data. Iteration is performed over these latter steps until convergence in values of missing data (to a predetermined threshold) was achieved. Missing data were not imputed for any other outcomes. IM daily diary data were imputed for one participant at week 4, 3 participants at week 12, and 3 participants at week 24.

IM daily diary data were presented as the number of intrusive memories reported by the participant per day, sequentially for the 7 days of the listed week, with days separated by a comma. NaN (Not a Number) indicates days where data were not reported.

Abbreviations are as above.

Figure S11. Primary outcome. Primary outcome histograms.

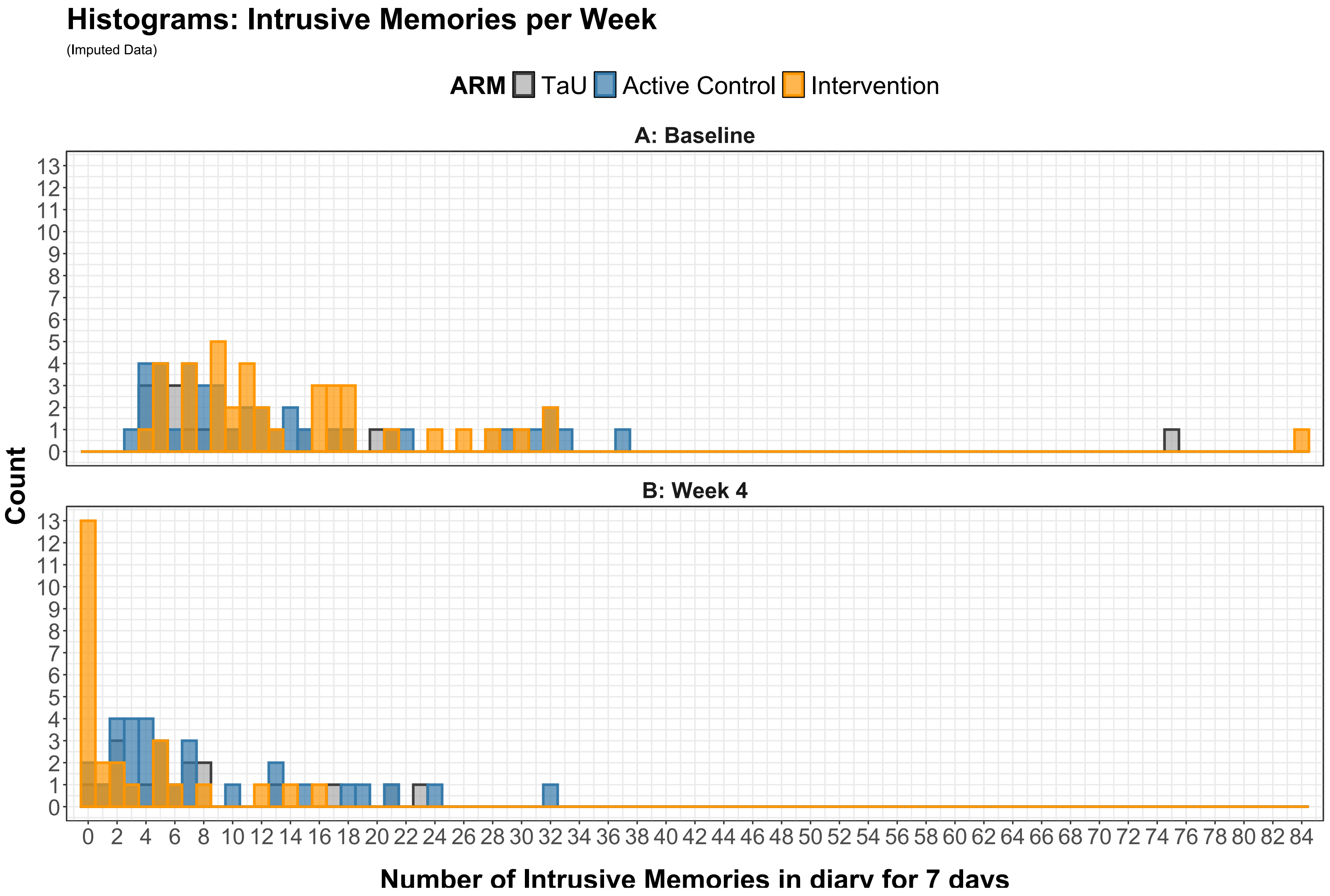

Supplementary Methods 1. Task Adherence for Imagery-Competing Task Intervention and Music-Listening Active Control Task

Adherence in the imagery-competing task intervention (ICTI) was defined as, during the initial guided session, completing all key components of the ICTI including: accurately identifying and briefly listing intrusive memories; choosing an intrusive memory to target; briefly bringing to mind the intrusive memory image before gameplay (so that the image is sufficiently clear, but not for so long that the participant becomes overly upset or distressed); engaging in sufficient uninterrupted Tetris® game play (c.20 min in total; range 15 - 25 min); and, during gameplay, actively using mental rotation.

The music-listening Active Control (AC) task was designed to match the imagery-competing task intervention in duration and structure and consisted of instructions, a BBC informational podcast about Mozart[14], multiple-choice questions, and listening to music for 20-minutes. Adherence in the AC task was defined as, during the initial guided session, completing all key task components including listening to the music (Mozart's String Duo No.1 K.423) for approximately 20-minutes without significant distractions which would prevent the participant from listening to the music.

Supplementary Methods 2. Training of Digital Navigators to Conduct the Guided Session

The researcher-guided sessions were delivered remotely by one person from a team of two research assistants (AM, ZI) who had a Masters level training in psychology but no clinical qualifications, and a clinical psychologist (LI). 78% of sessions were delivered by research assistants.

Prior to delivering guided sessions, researchers received training and corrective feedback in delivering the intervention and active control sessions protocols including adherence assessments. They were also trained in use of the digital IM diary in both ePRO® and i-spero® and how to interpret the graphs of incoming IM data in i-spero®.

Training on the ICTI and the IM diary was delivered by experienced clinical researchers (supervised by EAH). First, researchers received a four-session online training course covering the conceptual basis and research history of the intervention and how to identify and monitor the frequency of IMs. Thereafter, researchers were assessed on their competency at explaining the theoretical background and key components of the ICTI, and were required to meet the highest level of competency.

Second, trainee researchers were trained in use of the i-spero® digital platform to deliver the ICTI. This included being guided through the platform by the trainer, and the opportunity to become familiar with it as if a user.

Third, researchers and trainers switched roles, whereby the trainee guided the trainer through the researcher-guided session in the platform. Thus, for both ICTI and active control conditions, researchers completed role-play practice of sessions where they with discussion and feedback provided until meeting the top level of adherence. Trainees were also encouraged to role play with peers and colleagues.

Fourth, the first few RCT participant sessions (for both ICTI and AC groups) of the research assistants were observed in real-time by an experienced clinician (LI) who joined the session's video call. After the in vivo session, the research assistant was provided reflective/corrective feedback as necessary to ensure fidelity to protocols.

Fifth, occasionally in the trial, another researcher in the team joined on another's guided session (in vivo), to promote ongoing learning and reduce protocol drift.

Most sessions were audio recorded to aid protocol adherence.

Finally, supervision was ongoing throughout the trial to promote protocol adherence and to learn lessons from running the study. All staff had a weekly team meeting with EAH, as well as group supervision and real-time support as needed via telephone/video call with EAH/LI. Group supervision was fortnightly and included other researchers beyond the current study team running related studies.

Supplementary Methods 3. Modelling the total number of IMs recorded in week-4
We fitted a Bayesian Poisson linear mixed model (Observation Level Random Effects Poisson, ORLE) to analyse the primary outcome (IM count over multiple time-points)[15]. Baseline IM count and treatment assignment were fitted as fixed effects with a random intercept effect for participants. Bayesian hypothesis testing was performed using Bayes Factors[16].

For each participant (i):

- Y_i = total number of IMs in week-4
- $Baseline_i$ = baseline IM count
- Dichotomous treatment variables (AC_i and TAU_i) encode participant arm (i.e. AC has [AC=1, TAU=0], TAU [AC=0, TAU=1], ICTI [AC=0, TAU=0]
- $subj_i$ = dichotomous variable, equal to 1 if the observation is from the participant, and 0 if not
- γ_{oi} = random intercept for each participant i, accounting for participant-specific variations in the primary outcome
- The random-effects intercepts γ_{oi} follow a normal distribution with mean 0 and standard deviation σ_{γ_o}
- The model includes the unknown parameters α, β_1, β_2, β_3, σ_{γ_o}

$$Y_i \sim Poisson(\mu_i)$$

$$E(Y_i) = \mu_i$$

$$ln(\mu_i) = \alpha + Baseline_i\beta_1 + TAU_i\beta_2 + AC_i\beta_3 + \sum_{i=1}^{n} j_i\gamma_{0i}$$

$$\gamma_{0i} \sim N\left(0, \sigma^2_{\gamma_0}\right)$$

We tested alternative models for negative binomial or zero-inflated distributions if data were over dispersed or zero-inflated. Model fit was checked using residual plots, and selected based on root mean square error, Bayesian Information Criterion, Akaike Information Criterion, and Bayes Factor scores. Prior distributions for parameters were set as $N(0, 10^2)$, incorporating a realistic non-informative range centred on 0. Alternative priors were tested in sensitivity analyses to validate prior choices.

Bayes Factors were used to test hypotheses and compare the likelihood of one model relative to another. This allowed assessment of how the posterior distribution of the model has shifted relative to the prior when examining a parameter at a fixed point or range of values. The Bayesian framework allowed explicit specification of multiple models, in contrast to a frequentist approach which relies on a single model.

Intrusive Memory Daily Diary Measure. During the baseline week, and weeks 4 (Days 22-28), 12 (Days 78-84) and 24 (Days 162-168), all participants recorded the number of IMs experienced in a daily intrusive memory diary. This brief, daily measure has convergent validity with PTSD symptoms measured with the Impact of Events Intrusion subscale and is easy to complete, resulting in high adherence rates[17]. Moreover, symptom count data are easily interpretable by clinicians and participants, offering greater sensitivity compared to questionnaires with finite categories.

Priors and Model Specifications
Normal priors centred on zero with SD=10 were used for all fixed effect parameters, with alternative priors tested in sensitivity analyses. Results were mostly insensitive to prior changes (Supplementary Fig.S2).

Supplementary Methods 4. Imputation, Missing Data and Outliers

For IM weekly count outcomes, missing values were imputed for participants with partial completion (at least one day of IM diary). As these data were collected sequentially over time, we used time series methods[18] and an expectation maximisation algorithm[19] to impute missing values by taking expectations from available data and applying Poisson likelihood with correlated errors. Iterative updates continued until convergence to a pre-determined threshold was achieved. Put simply, we used available data to generate a model for each participant, which was then used to estimate missing values for unobserved days. This approach was consistent with the interim analyses undertaken in the previous GAINS-01 trial[20]. Table S26 presents details of imputed data. No imputation was performed for other outcomes.

We note that full Bayesian modelling of longitudinal data offers an alternative imputation approach. However, in the case of IM weekly count data the time series structures are too short to evaluate realistic correlation structures.

For IM weekly count outcomes, missing values were imputed for participants with partial completion ($\geq$1 day in IM diary), and a negative binomial linear mixed model was used with a log link function.

Supplementary Methods 5. Analysis of secondary, exploratory (not pre-specified) and mechanistic (not pre-specified) outcomes

Secondary Outcome Analyses
Secondary outcomes were modelled based on variable type, with all likelihood functions across models. . Participants who contributed no post-baseline observations for an outcome were defined as not assessable for that outcome. All models included baseline score, treatment arm, and week fitted as fixed effects, with a random intercept effect for participants, and interaction effects between treatment arm and week.

- Y_i - outcome score for subject *i* (i.e. total IMs)
- $Baseline_i$ – outcome score in the baseline week for subject *i*.
- Dichotomous treatment variables (AC, TAU) with values equal to 1 for participants in those arms and zero otherwise (ICTI is the reference group)
- Dichotomous week variables (t_{12}, t_{24}) with values equal to 1 for referring to weeks-12 and 24 respectively and zero otherwise (week-4 is the reference week)
- *subj* - dichotomous variable=1 if the observation is from the participant (subject), and =0 if not
- The model, where α, $\beta_{1\text{-}9}$,$\sigma_{\gamma 0}$ are unknown model parameters, is:

$$Y_{ij} \sim Dist(\mu_{ij})$$

$$E(Y_{ij}) \sim \mu_{ij}$$

$$Link(\mu_{ij}) = \alpha + Baseline_i\beta_1 + TAU_i\beta_2 + AC_i\beta_3 + t_{12}\beta_4 + t_{24}\beta_5 + TAU_it_{12}\beta_6$$

$$+TAU_it_{24}\beta_7 + AC_it_{12}\beta_8 + AC_it_{24}\beta_9 + \sum_{i=1}^{n} j_i\gamma_{0i}$$

$$\gamma_{0i} \sim N(0, \sigma_{\gamma_0}^2)$$

with the following priors:
$\alpha \sim t(3,2.5)$
$\beta i \sim N(0,10^2)$
$\sigma_{\gamma_0} \sim t(3,2.5)$

For count outcomes (e.g., number of IMs), a negative binomial linear mixed model was used with a log link function.

Continuous outcomes (e.g., PCL-5) and ordinal outcomes with more than 10 distinct groups were modelled, assuming a normal distribution with an identity link.

For ordinal outcomes (e.g., GAD-2, PHQ-2), we used a cumulative regression model with a logit link function where categorisation of the latent variable Y_i into K–1 ordered cut-off thresholds was used.

Priors and Model Specifications
Normal priors centred on zero with SD=10 were used for all fixed effect parameters, with alternative priors tested in sensitivity analyses. Results were mostly insensitive to prior changes. While some secondary outcome measures showed moderate sensitivity to the choice of priors on the parameter estimates, in all such cases, estimates derived using less restrictive priors (N~(0,10^2), N~(0,20^2)) were consistent, supporting the overall robustness of results. Therefore, we maintained normal priors as a reasonable non-informative choice. The SD random effect parameter σ_{γ_0} was restricted to be positive and takes a half student-t prior 3 degrees of freedom, scale 2.5.

Posterior probability estimation
The posterior probability of the number of IMs in ICTI being fewer than in AC and TAU was estimated at weeks -4, -12, and -24. For each arm, at each timepoint, over 100,000 draws of the number of IMs were generated from the secondary outcome analysis model using posterior epred in the R brms package (v 2.23.0). Then, differences between the arms were computed. The probability was calculated as the number of observations where the difference was less than zero over the total number of observations.

The posterior probability of a clinically meaningful difference in PCL-5 scores between ICTI and AC or TAU was estimated using a similar method. A clinically meaningful difference was defined as ≥5 points on the PCL-5,

based on Ehlers and colleagues[26]. The probability was calculated as the number of observations where the difference between the arms was greater than or equal to five over the total number of observations.

Exploratory outcome analyses (not pre-registered)
Analyses followed similar framework as secondary outcomes, using week and baseline data as applicable. Priors matched those used for secondary outcomes (OSF; https://osf.io/cs6hn/).

Probability of ICTI eliminating IMs (*post-hoc)*
Time series fitting Bayesian regression models were used to predict IM counts at week T as a function of IM counts at week T-t, using a Poisson mixed model. IM counts at the previous time-point and treatment assignment were fitted as fixed effects with a random intercept for participants. Prior distributions for all parameters were set as $N(0,10^2)$, incorporating a realistic non-informative range of values centred on 0. Draws from expected values of posterior predictive distributions were used to compute probability distributions of IM counts at different time-points across arms.

Mechanistic analysis of memory reactivation (*post-hoc*)
A Bayesian logistic regression (with normal priors on the unknown parameters) was used to assess how memory reactivation (vividness) and task (gameplay) influenced ICTI success (defined as zero IMs the following day). Vividness ratings (3 highest categories on 5 point scale: 1= No image at all; 2=Vague and dim; 3=Moderately clear and vivid; 4=Clear and reasonably vivid; 5= Perfectly clear and as vivid as normal vision), Tetris® scores (continuous variable), and participant ID (random effect), were used to model probability of zero IMs. Model analysis and outcomes were evaluated using posterior distributions and BFs comparing the full model (vividness and Tetris® score) against models with vividness or gameplay alone.

Missing data analysis
To explore potential differences between the arms in the missingness of the primary outcome we conducted Bayesian contingency table analyses using BayesFactor package (v0.9.12-4.7).

A Bayesian logistic regression model (with normal priors on the unknown parameters, except the intercept which was t(3,2.5)) was used to test if the following baseline variables predicted data missingness: age, gender, ethnicity, trauma count, number of baseline IMs, and weekly hours worked. This compared against a null model with only an intercept.

PTSD severity score subgroup analysis
Participants were grouped based on baseline PCL-5 score (<33 or ≥33). A continuous model with group, arm, and week as fixed effects and their interactions was fitted, including a random intercept per subject. Priors were as with secondary outcomes.

CEQ total score analysis
CEQ total score was calculated by rescaling questions 4 and 6 to values 1-9, then summing all scores. This was modelled using a normal distribution with arm as a fixed effect. Since the questionnaire was completed at a single time-point, week was not included as a fixed effect. All fixed effects had priors of $N(0,10^2)$.

Supplementary Methods 6. Further considerations in choosing intrusive memories as the treatment target

Our participants were NHS healthcare professionals exposed to work-related psychological trauma whilst caring for hospitalised patients during the COVID-19 pandemic. The escalated risk to healthcare staff of post-traumatic stress due to exposure to trauma at work was highlighted early in the pandemic.[21] This group developed debilitating symptoms of PTSD (posttraumatic stress disorder)[22] as a result, impairing individual functioning and impacting patient care, as was highlighted repeatedly at the UK Covid Inquiry.[23]

In setting up the GAINS studies, the lived experience of traumatised healthcare staff, and our work with the Intensive Care Society taught us that often and understandably:

- Doctors, nurses and other healthcare staff, neither wish to be given a diagnosis of PTSD nor take part in research that gives them a mental health diagnosis.
- Due to stigma, healthcare staff, can be reluctant to take up trauma treatment via psychiatry/psychology services.
- To access trauma treatment, healthcare staff would prefer to self-identify their own symptoms.
- Accessing services to treat work-related trauma is challenging for healthcare staff, with few services available to support staff who work mainly on a shift/rota basis and long waiting lists for the limited services that are available, with implications for the healthcare services in which people deliver care.
- Trauma exposure was not over but ongoing and people wanted to remain in or return to work. Brief and repeatable treatment options were needed.

Therefore, we designed the GAINS-02 study so that after traumatic exposure at work, healthcare staff could:

- Self-refer based on one core symptom of post-traumatic stress (intrusive memories are a distinct and recognisable hallmark symptom of PTSD, see below)
- Self-treat after one guided session
- Access our service directly via a digital platform, whenever suited them, without the need to meet a mental health clinician nor attend a mental health service.
- Complete the guided intervention session in one hour.
- Be able to the intervention even in a public space, if necessary, since it had the appearance of playing a game.

In addition to the rationale outlined in the Introduction, there were several other considerations in selecting intrusive memories (IMs) as our primary outcome measure including:

- Diary studies indicate convergent validity between IMs and other PTSD symptoms[17].
- The concept of intrusive memories of trauma is readily understood by people who have experienced trauma, making it straightforward to identify if they have them or not. IMs are commonly referred to as “flashbacks” in everyday language. In the study there were explained as “intrusive unwanted memories of traumatic events, that is, images that pop into the mind’s eye suddenly, such as seeing a patient’s face or an item of equipment (information sheet)” and “mental images from a traumatic event that pop suddenly into your mind when you don’t want them to” (study diary). IMs can be either very clear and vivid or less vivid and fleeting. They are typically distressing, affect one’s concentration and the ability to do everyday tasks, and can cause someone to lose touch with the here and now. IMs have been associated with the “worst moments” (hotspots) in a trauma.[24]
- Thus, study participants can readily self-assess whether they meet the study's entry criteria (3 or more IMs per week), rather than requiring a mental health specialist to assess them.
- It is straightforward to count IMs. Unlike when completing psychiatric symptom questionnaires, a count does not require further introspection e.g. a judgment about the symptom such as its emotionality / distress and is less time consuming.
- Symptom count data can offer greater sensitivity compared to symptom questionnaires with finite categories.

Supplementary Methods 7. Ethnicity data collection and analysis

As a Baseline assessment, participants self-identified their ethnicity by typing into a free text box and using the examples of the following categories: Asian, Black, Chinese, Mixed, White, and Other. Ethnicity was treated as a sociocultural descriptor. Data on self-reported ethnicity were used for descriptive purposes.

References for Secondary Outcome Measures

Posttraumatic Stress Disorder Checklist for DSM-5, 20-item (PCL-5): Weathers FW, Litz BT, Keane TM, Palmieri PA, Marx BP, Schnurr PP. The PTSD checklist for DSM–5 (PCL-5). https://www.ptsd.va.gov/professional/assessment/adult-sr/ptsd-checklist.asp (accessed Mar 31, 2025).
Sleep Condition Indicator, 2-item (SCI-02): Espie CA, Kyle SD, Hames P, Gardani M, Fleming L, Cape J. The sleep condition indicator: a clinical screening tool to evaluate insomnia disorder. *BMJ Open*. 2014; **4:** e004183.
Patient Health Questionnaire, 2-item (PHQ-2): Kroenke K, Spitzer RL, Williams JBW. The patient health questionnaire-2: validity of a two-item depression screener. *Medical Care.* 2003; **41:** 1284-92.
Generalised Anxiety Disorder, 2-item (GAD-2): Kroenke K, Spitzer RL, Williams JB, Monahan PO, Löwe B. Anxiety Disorders in Primary Care: prevalence, impairment, comorbidity, and detection. *Annals of Internal Medicine*. 2007; **146:** 317-25.
WHO Disability Assessment Schedule, 12-item (WHODAS): Axelsson E, Lindsäter E, Ljótsson B, Andersson E, Hedman-Lagerlöf E. The 12-item Self-Report World Health Organization Disability Assessment Schedule (WHODAS) 2.0 Administered Via the Internet to Individuals With Anxiety and Stress Disorders. *JMIR Ment Health.* 2017; **4:** e58.
EuroQol-5D, 5-Level (EQ-5D-5L): Herdman M, Gudex C, Lloyd A, Janssen MF, Kind P, Parkin D, et al. Development and preliminary testing of the new five-level version of EQ-5D (EQ-5D-5L). *Quality of Life Research*. 2011; **20:** 1727-36.
Scale of work engagement and burnout (SWEBO): Hultell D, Gustavsson JP. A psychometric evaluation of the Scale of Work Engagement and Burnout (SWEBO). *Work*. 2010; **37:** 261-74.
Intention to leave job (ITL): Cohen A. An examination of the relationship between work commitment and work outcomes among hospital nurses. *Scand. J. Manag*. 1998; **14:** 1-7.
Intrusive memory Questionnaire. Iyadurai L, Highfield J, Kanstrup M, et al. Reducing intrusive memories after trauma via an imagery-competing task intervention in COVID-19 intensive care staff: a randomised controlled trial. *Transl Psychiatry* 2023; **13:** 290–15.

Figure S12. Study Schematic and Follow-up Time Points.

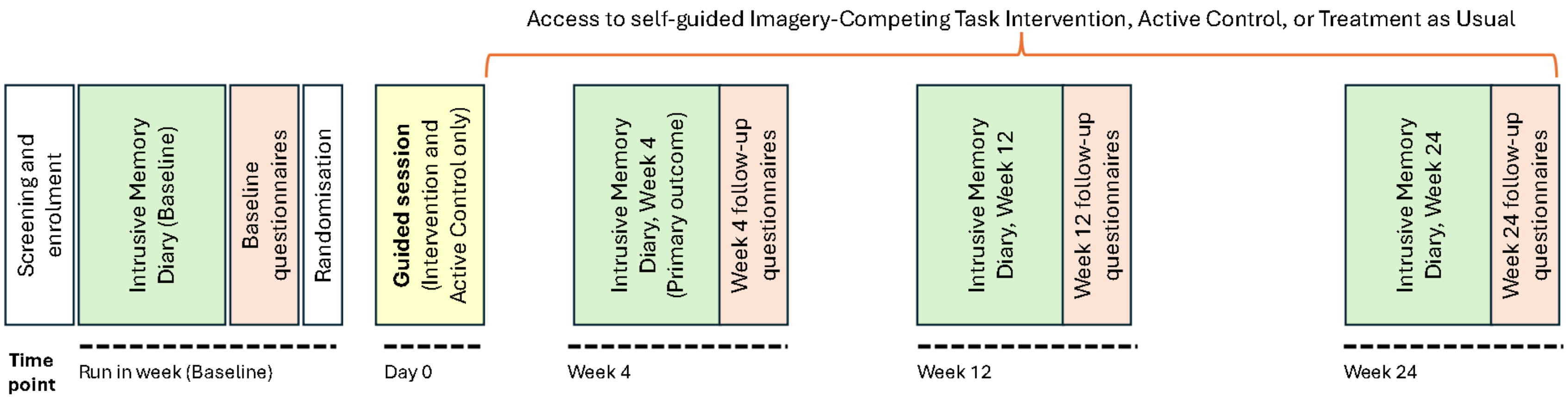

Note, *after screening and enrolment, participants recorded baseline intrusive memory (IM) counts in a daily diary during a run-in week, completed baseline questionnaires and were randomised to study arms. On Day 0, participants in the ICTI and Active Control arms attended the session guided by a researcher (digital navigator). Afterward, they accessed their self-guided tasks for 24-weeks. All arms could access treatment as usual during this period. At weeks 4 (primary outcome), 12 and 24, participants completed the intrusive memory diary for 7 days and answered follow-up questions. Expectancy of treatment effect was collected using the Credibility and Expectancy Questionnaire (CEQ) on Day 0, prior to the Guided session for ICT and active control participants, and the day after randomisation for treatment as usual participants.*

Figure S13. Number of intrusive memories over time per group.

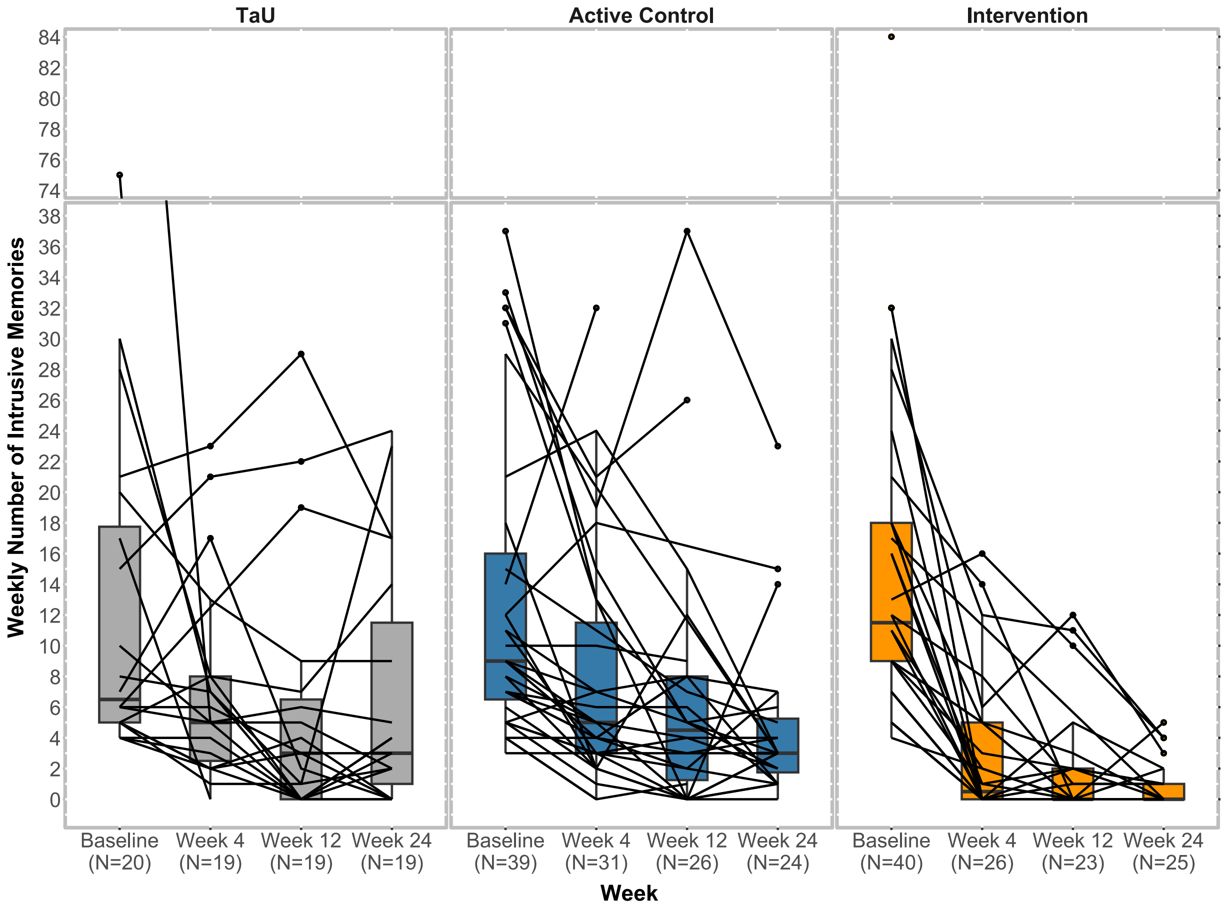

Note, *boxplots for number of intrusive memories at baseline, week 4, week 12, and week 24 for each group, showing the median (midline), the third and first quartile (upper and lower limits of box), and whiskers (1·5 × IQR). Outliers are represented by dots (>1·5 times the IQR above third quartile or below first quartile). All outliers are included in these boxplots.*

References

1. R Core Team. R: A language and environment for statistical computing (version 4.1.2) [Internet]. Vienna: R Foundation for Statistical Computing; 2018. Available from: https://www.R-project.org/.
2. Lee MD, Wagenmakers E-J. Bayesian cognitive modeling: A practical course. New York, NY, US: Cambridge University Press; 2013.
3. Weathers FW, Litz BT, Keane TM, Palmieri PA, Marx BP, Schnurr PP. The PTSD Checklist for DSM–5 (PCL-5). Scale available from www.ptsd.va.gov. 2013.
4. Espie CA, Kyle SD, Hames P, Gardani M, Fleming L, Cape J. The Sleep Condition Indicator: a clinical screening tool to evaluate insomnia disorder. BMJ Open. 2014;4(3):e004183.
5. Kroenke K, Spitzer RL, Williams JB, Monahan PO, Löwe B. Anxiety Disorders in Primary Care: Prevalence, Impairment, Comorbidity, and Detection. Annals of Internal Medicine. 2007;146(5):317-25.
6. Kroenke K, Spitzer RL, Williams JBW. The Patient Health Questionnaire-2: Validity of a Two-Item Depression Screener. Medical Care. 2003;41(11):1284-92.
7. Hultell D, Gustavsson JP. A psychometric evaluation of the Scale of Work Engagement and Burnout (SWEBO). Work. 2010;37:261-74.
8. Axelsson E, Lindsäter E, Ljótsson B, Andersson E, Hedman-Lagerlöf E. The 12-item Self-Report World Health Organization Disability Assessment Schedule (WHODAS) 2.0 Administered Via the Internet to Individuals With Anxiety and Stress Disorders. JMIR Ment Health. 2017;4(4):e58.
9. Herdman M, Gudex C, Lloyd A, Janssen MF, Kind P, Parkin D, et al. Development and preliminary testing of the new five-level version of EQ-5D (EQ-5D-5L). Quality of Life Research. 2011;20(10):1727-36.
10. Cohen A. An examination of the relationship between work commitment and work outcomes among hospital nurses. Scandinavian Journal of Management. 1998;14(1):1-17.
11. Iyadurai L, Highfield J, Kanstrup M, Markham A, Ramineni V, Guo B, et al. Reducing intrusive memories after trauma via an imagery-competing task intervention in COVID-19 intensive care staff: a randomised controlled trial. Translational psychiatry. 2023;13(1):290-15.
12. Revicki DA, Irwin D, Reblando J, Simon GE. The Accuracy of Self-Reported Disability Days. Medical Care. 1994;32(4):401-4.
13. Devilly GJ, Borkovec TD. Psychometric properties of the credibility/expectancy questionnaire. Journal of Behavior Therapy and Experimental Psychiatry. 2000;31(2):73-86.
14. BBC. What is it about Mozart? [Internet]. BBC Sounds: BBC; 2016 [cited 11th March 2025]. Podcast. Available from: https://www.bbc.co.uk/sounds/play/b078n3r2
15. Harrison XA. Using observation-level random effects to model overdispersion in count data in ecology and evolution. PeerJ. 2014;2.
16. Kass RE, Raftery AE. Bayes Factors. Journal of the American Statistical Association. 1995;90(430):773-95.
17. Singh L, Ahmed Pihlgren S, Holmes EA, Moulds ML. Using a daily diary for monitoring intrusive memories of trauma. International Journal of Methods in Psychiatric Research. 2023;32(1):e1936.
18. Chatfield C. The Analysis of Time Series: An Introduction. Sixth Edition ed. New York: Chapman and Hall/CRC; 2003.
19. Dempster AP, Laird NM, Rubin DB. Maximum Likelihood from Incomplete Data Via the EM Algorithm. Journal of the Royal Statistical Society: Series B (Methodological). 1977;39(1):1-22.
20. Ramineni V, Millroth P, Iyadurai L, Jaki T, Kingslake J, Highfield J, et al. Treating intrusive memories after trauma in healthcare workers: a Bayesian adaptive randomised trial developing an imagery-competing task intervention. Molecular psychiatry. 2023;28(7):2985-94.
21. Holmes EA, O'Connor RC, Perry VH, Tracey I, Wessely S, Arseneault L, Ballard C, Christensen H, Silver RC, Everall I, Ford T. Multidisciplinary research priorities for the COVID-19 pandemic: a call for action for mental health science. The lancet psychiatry. 2020;7(6):547-60.
22. American Psychiatric Association. Diagnostic and Statistical Manual of Mental Disorders. 5th ed. Washington, DC: American Psychiatric Association; 2013
23. Summers C, Suntharalingam G. Intensive care: the last line of defence. UK COVID-19 Inquiry (Module 3: The impact of the Covid-19 pandemic on healthcare systems in the UK); 2024 Jul. Report No.: INQ000474255. Available from: https://covid19.public-inquiry.uk/documents/inq000474255-expert-report-from-professor-charlotte-summers-and-dr-ganesh-suntharalingam-titled-intensive-care-the-last-line-of-defence-dated-22-07-2024/
24. Holmes EA, Grey N, Young KA. Intrusive images and "hotspots" of trauma memories in posttraumatic stress disorder: An exploratory investigation of emotions and cognitive themes. Journal of Behavior Therapy and Experimental Psychiatry. 2005;36(1):3-17.

25. NHS Digital. NHS Hospital and Community Health Services (HCHS): Summary statistics for HCHS staff in England [Internet]. 2022 [accessed 2023 Mar 21]. Available from: https://digital.nhs.uk/data-and-information/publications/statistical/nhs-workforce-statistics/december-2022

26. Ehlers A, Wild J, Warnock-Parkes E, et al. Therapist-assisted online psychological therapies differing in trauma focus for post-traumatic stress disorder (STOP-PTSD): A UK-based, single-blind, randomised controlled trial. *The Lancet Psychiatry*. 2023;**10**(8):608-22.
